# Nickel Toxicity in Plants

**DOI:** 10.3390/ijms27135942

**Published:** 2026-07-01

**Authors:** Ilya V. Seregin, Anna D. Kozhevnikova

**Affiliations:** K.A. Timiryazev Institute of Plant Physiology, Russian Academy of Sciences, Botanicheskaya St., 35, Moscow 127276, Russia; kozhevnikova.anna@gmail.com

**Keywords:** enzyme activity, mineral nutrition, nickel, photosynthesis, plant growth and development, respiration, seed germination, structure and permeability of membranes, water regime

## Abstract

Nickel (Ni) is a ubiquitous trace metal and an essential ultra micronutrient. It is a constituent of several enzymes and is currently known to be involved in a number of physiological processes in plants, including nitrogen metabolism, growth and seed germination. However, at elevated concentrations, Ni exerts multiple toxic effects on various physiological processes, which leads to impaired plant growth and morphogenesis, and reduced productivity. This review summarizes the current knowledge of the mechanisms of Ni toxicity, analyzing its effects on enzyme activity, membrane structure and functioning, antioxidant and glyoxalase systems, mineral nutrition, water regime, photosynthesis, plant growth and morphogenesis. Nickel effects on excluder plants, which accumulate Ni primarily in their roots, and hyperaccumulator plants, which accumulate Ni primarily in their shoots, are compared. Studying the mechanisms of Ni toxicity may substantially contribute to an integral modern approach to investigating plants under constantly changing environmental conditions. It is essential for understanding the full spectrum of physiological and biochemical reactions in plants that are involved in plant tolerance to Ni. Furthermore, understanding of the mechanisms of Ni growth inhibitory effects is important for the enhancement of agricultural techniques aimed at increasing crop productivity worldwide. It is also crucial for the development of approaches used in phytoremediation and phytomining technologies.

## 1. Introduction

Nickel (Ni) is one of the ubiquitous trace elements that can enter the biosphere through both natural processes and anthropogenic activities. Natural sources of Ni include volcanic activity, soil and rock weathering, and forest fires. Anthropogenic activities such as mining, smelting, welding, electroplating, production of alloys, combustion of fossil fuels, and disposal of industrial waste and sewage sludge, have dramatically increased environmental pollution with Ni [1,2]. Nickel is the 22nd most abundant element in the Earth’s crust [3] and one of 23 metal pollutants posing a serious threat to ecosystems and human health [1]. The total Ni content typically found in natural soil ranges from 10 to 1000 mg kg^−1^, whereas in contaminated soils it may reach 9000–26,000 mg kg^−1^ [3,4,5]. Globally, soil pollution with metals has a dramatic impact on crop productivity and remains a grave problem concerning food security and human health. Ultramafic or serpentine soils weathered from serpentinites typically contain from 500 to 10,000 mg kg^−1^ Ni and are also characterized by high cobalt (Co), and chromium (Cr) contents, low nitrogen (N), phosphorus (P), and potassium (K) contents, and low calcium (Ca)–magnesium (Mg) ratio, which makes them a challenging environment [6,7,8]. Ultramafic soils host metallophyte floras with a high number of endemic and highly specialized plant species that have developed mechanisms allowing them to grow in these stressful environments [9,10,11].

Most of plant species, even the ones growing on metalliferous soils, are so called ‘excluders’ characterized by reduced metal translocation to aboveground organs. The Ni content in their shoots usually does not exceed 5–15 μg Ni g^−1^ dry weight when they grow on uncontaminated soil and is around 25–50 μg Ni g^−1^ dry weight when they grow on ultrabasic rocks [12,13]. A smaller group of plants that accumulate specific metals or metalloids at extremely high concentrations (above certain threshold values) in their shoots and maintain lower metal concentrations in their roots are named ‘hyperaccumulators’ [14,15,16,17,18]. The hyperaccumulators of Ni are capable of accumulating more than 1000 μg Ni g^−1^ dry weight in shoots when growing in nature without visible symptoms of toxicity [12,16,17,18]. Currently, more than 500 Ni-hyperaccumulating species from 54 families have been identified globally, with the ultramafic regions of Cuba, New Caledonia, Turkey and Brazil being “hotspots” of their biodiversity [16,17,19,20,21,22,23]. Metal-hyperaccumulating plants are characterized by efficient mechanisms of metal transport and detoxification [14,24,25,26,27,28,29,30,31], and therefore, are used in various ‘green’ technologies aimed at metal extraction and remediation of metal-contaminated soils [32,33,34,35,36,37]. The mechanisms of Ni hyperaccumulation and related hypertolerance are currently being actively studied on the Ni hyperaccumulator species from genera Noccaea and Odontarrhena [22,26,31,38,39,40,41,42,43,44].

At low concentrations, Ni is beneficial for normal plant growth and development, and is considered an essential micronutrient [45,46] or ultra-micronutrient [47,48]. The first clear evidence of Ni essentiality for legumes was shown by Eskew et al. [49]. Later, Brown et al. [50] demonstrated the essentiality of Ni for non-legumes. Under Ni deficiency, *Hordeum vulgare* failed to produce viable grains due to the disruption of the maternal plant’s normal grain filling and maturation processes that occur following the formation of the grain embryo. The embryonic root developed poorly or stayed undeveloped [50]. In some species, Ni deficiency leads to interveinal chlorosis as well as appearance of deformed leaves, a symptom known as “mouse ear” disorder [51]. As an essential micronutrient, Ni is required for most plant species in minute quantities (0.01–10 μg g^−1^ dry weight) to complete their life cycle [13,52]. Although Ni is a minor element of the Earth’s crust, it has played a significant role in the evolution of life [53]. Nickel is a vital constituent of several metalloenzymes in bacteria and plants, e.g., urease, glyoxylase I, Ni-superoxide dismutase, etc. Thus, it contributes to many biological processes, including N metabolism, seed germination, plant growth and senescence, and also promotes plant survival under the influence of biotic and abiotic stress factors [3,54,55,56,57].

High (supraoptimal) Ni concentrations in the growth medium are toxic for plants [51], which was first noticed in 1893 by Hasselhoff [6]. Critical toxicity concentrations in crop species range between 10 µg Ni g^−1^ dry weight for Ni-sensitive species and 50 µg Ni g^−1^ dry weight for moderately tolerant ones [51], whereas for Ni hyperaccumulator species, they usually exceed 1000 μg Ni g^−1^ dry weight [16,17,18]. Therefore, it has been proposed to consider Ni a potentially toxic element [58]. The symptoms of Ni toxicity include growth inhibition, impaired development, including morphological, anatomical and ultrastructural changes, leaf chlorosis and necrosis, wilting, decrease in seed yield, etc. [1,2,3,47].

Upon entering the cells, Ni exerts multiple toxic effects on various physiological processes. The cellular and physiological mechanisms of its toxicity include the inhibition of enzyme activity; development of oxidative stress; inhibition of the uptake and translocation of macro- and micronutrients; changes in the permeability of membranes due to the modifications in their composition; reduction in the stomatal conductance and transpiration rate; decline in the rate of photosynthesis due to impaired electron transport, inhibited chlorophyll biosynthesis and activities of the Calvin cycle enzymes, and CO_2_ deficiency resulting from stomatal closure; impaired cell division due to chromosomal aberrations and disarrangement of the cytoskeleton; restricted cell elongation due to metal binding to cell wall material, etc. [1,2,3,47].

Several specific and nonspecific systems aimed at maintaining metal homeostasis and reducing the manifestation of its toxic effects on plants are activated in response to Ni-induced stress. They include the induction of the activity of antioxidant enzymes for the neutralization of reactive oxygen species; induction of biosynthesis of osmolytes, such as proline and polyamines; changes in the cell wall composition such as callose and suberin deposition to restrict metal translocation; biosynthesis of metal-chelating ligands, e.g., nicotianamine, histidine, organic acids, and efficient metal sequestration in metabolically inactive cellular compartments, e.g., the vacuole [1,2,3,47]. These systems are often more efficient in Ni-tolerant species, including Ni hyperaccumulators [14,17,26,31].

The current review summarizes and analyzes the extensive data available in the literature on the mechanisms of Ni toxicity, including its effects on enzyme activities, antioxidant and glyoxalase systems, structure and functioning of membranes, mineral nutrition, transpiration, water balance, photosynthesis, respiration, as well as plant growth and morphogenesis in Ni-hyperaccumulating and non-hyperaccumulating species.

## 2. The Effects of Ni on Enzyme Activities

Metals may influence all physiological processes, but the sensitivity of different processes to their effects varies greatly. One of the most important changes in cellular metabolism caused by heavy metals is the alteration of enzymatic activity. Directly interacting with enzymes, metal ions often inhibit their activity. Metal concentration causing a 50% inactivation of the majority of enzymes, named the enzyme inactivation constant (*K_i_*), differes depending on the metal: Ag^+^, Hg^+^, Cu^2+^ (10^−7^–10^−5^) > Cd^2+^ (10^−6^–3 × 10^−5^) > Zn^2+^ (10^−5^–10^−4^) > Pb^2+^ (10^−5^–2 × 10^−4^) > Ni^2+^ (10^−5^–6 × 10^−4^) > Co^2+^ (2 × 10^−4^–3 × 10^−4^) [59,60].

**Figure 1:**
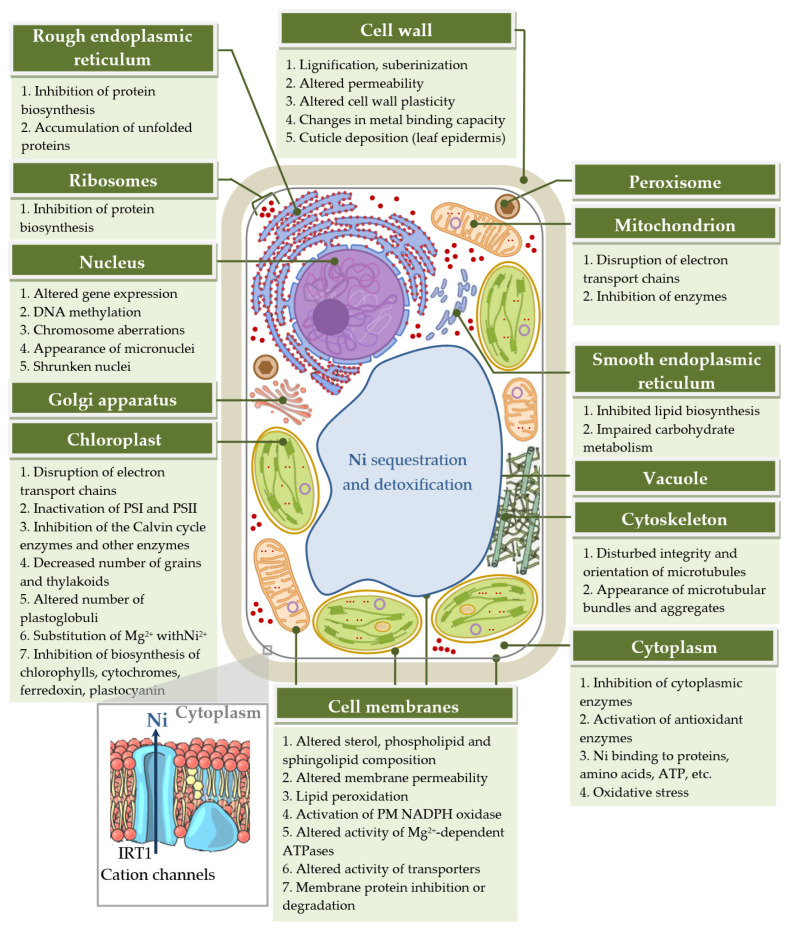
Toxic effects of Ni at the cellular level. Nickel enters the cell via nonselective iron-regulated transporter 1 (IRT1) or, possibly, via non-selective cation channels localized in the plasma membrane. Once in the cell, Ni exerts multiple toxic effects on various physiological processes.

In most cases, metal-induced enzyme inactivation is caused by metal interaction with O-, N- or S-containing groups therein. Metals may block the groups in the reaction center of the enzyme, as well as the groups responsible for stabilizing the quaternary structure of the protein, resulting in a change in the conformation of the enzyme, which is accompanied by a decrease in its activity (Figure 1). According to ligand-based classification of metals [61], Ni^2+^ is considered a borderline element that can form complexes with carboxyl and sulfhydryl groups. Thus, in plant cell, Ni^2+^ ions bind strongly to N, S and O atoms and show a high affinity for sulfhydryl and disulphide groups [6]. Possibly, the interaction of Ni with sulfhydryl groups is one of the mechanisms of its toxic effects on Mg^2+^-dependent ATPases of the plasma membrane in vitro, although the binding of Ni^2+^ ions to ATP is also possible, resulting in a decrease in the amount of substrate for ATPase [62]. In root cells of *Cucumis sativus*, the hydrolytic and the transporting activity of H^+^-ATPase in the plasma membrane were decreased at 10 and 100 µM Ni possibly due to the post-translational modification of its proteins [63]. However, the activity of the plasma membrane H^+^-ATPase was not inhibited in the roots [64] or even increased in the shoots [65] of *Oryza sativa* at 0.5 mM Ni (Table 1). Such opposite Ni effects on the activity of the plasma membrane H^+^-ATPase may be related to different tolerance of the species and/or plant organs, as well as different experimental conditions. The stimulatory effect of Ni on Mg^2+^-dependent ATPases in the plasma membrane of *Oryza sativa* shoot cells was suggested to result from Ni-induced changes in the sterol and phospholipid composition of the plasma membrane, which, in turn, led to a change in the ATPase activity [65]. Nickel can also inactivate enzymes if their active centers include histidine, since the affinity of Ni for N-containing ligands is higher than that for S-containing ones. This to some extent determines the specificity of Ni toxic effects.

Binding of different metal ions to sulfhydryl groups can lead to similar damage of the secondary structure of proteins, resulting in the changes in the activities of enzymes, and, consequently, impairment of metabolic processes [6]. Therefore, when analyzing metal effects, it is important to evaluate whether the observed effects are metal-specific or not. Moreover, metal toxic effects on the activity of enzymes in vitro is not always consistent with their effects studied at similar metal concentrations in vivo [121]. This may be attributed to the efficiency of cellular mechanisms of metal detoxification manifested in vivo, such as binding to different ligands and sequestration in the vacuoles, as well as to the physiological barriers restricting metal entry into the cytoplasm, such as, for example, metal binding to root mucilage compounds and cell wall material during the uptake.

General decrease in the enzyme activity may also be observed when the content of substrate in the cell is decreased. For example, the decrease in the activity of nitrate reductase in *Oryza sativa* exposed to 50–200 μM Ni was associated with a decrease in the nitrate content in leaves, where nitrate reduction took place [95]. Negative effects of Ni on nitrate reductase activity were also observed under Ni excess in *Brassica juncea* [66,67,91], *Beta vulgaris* [90], *Catharanthus roseus* [70], *Glycine max* [92], *Solanum lycopersicum* [72], *Triticum aestivum* [99,100], *Oryza sativa* [94,96,97], *Verbascum olympicum* [101], *Vigna radiata* [73,74] and *Zea mays* [103] (Table 1). Decreased availability of NO_3_^−^ to shoots could also diminish the transcript levels of *OsNR1* gene and reduce the stability of nitrate reductase mRNAs [94]. The decrease in the activity of nitrate reductase may also be connected to its degradation by reactive oxygen species (ROS), whose level rises as a result of Ni toxic effects [94,99], or with the decrease in the uptake of molybdenum (Mo), which is a cofactor of nitrate reductase [101].

The activities of nitrite reductase and glutamine synthetase, which largely depend on the amount of nitrates in the cytoplasm and the activity of nitrate reductase, often decreased in Ni-treated plants [90,94,95,96,99] (Table 1). In *Oryza sativa*, the activities of glutamate synthase, glutamate oxaloacetate transaminase and glutamate pyruvate transaminase also decreased, whereas the activities of glutamate dehydrogenase, alanine aminotransferase and aspartate aminotransferase increased in response to Ni [94,96]. This was followed by a downregulation of the expression level of *OsNiR*, *OsGS2*, *OsFd-GOGAT*, and *OsNADH-GOGAT*, whereas the expression of *OsGDH1* was upregulated [94]. Activities of key enzymes of N assimilation, i.e., nitrite reductase, glutamine synthetase, glutamate dehydrogenase (both aminating and deaminating), alanine aminotransferase and aspartate aminotransferase in the roots and shoots of Ni-treated seedlings of *Oryza sativa cvs.* Malviya-36 and Pant-12 growing in sand cultures depended on Ni concentration (200, 400 µM) and duration of treatment (5–20 days) [96]. In contrast to *Oryza sativa*, in the roots of *Triticum aestivum*, the activity of NADH-glutamate dehydrogenase decreased after 4–7 days of treatment with 100 µM Ni, which may be related to the accumulation of NH_4_^+^ in these organs [99]. The activities of alanine aminotransferase and aspartate aminotransferase in the roots of *Glycine max* decreased at 200 µM Ni [104]. At lower Ni doses, the activities of the enzymes of N metabolism usually increased [92,98,102] (Table 1).

Two metalloenzymes engaged in N metabolism have Ni in their active sites. The first one is urease, as first shown for *Canavalia ensiformis* and later for other plant species [47,55,122,123,124,125], and the second one is nickel–iron ([NiFe]) hydrogenase [126]. Urease hydrolyses urea into carbon dioxide and ammonia [47,55]. The latter is involved in various anabolic reactions, in particular, in the biosynthesis of glutamine from glutamic acid by glutamine synthetase [47]. The activity of urease increases in the presence of Ni [92,102,118,120] (Table 1). Hidden or latent Ni deficiency in plants reduces urease activity, impairs N metabolism and causes urea toxicity resulting in leaf tip necrosis and the chlorosis of old leaves [47,127]. On the contrary, the increase in urease activity appears to be a response to arginine degradation and the subsequent increase in the concentration of cytosolic urea [92,102,118,120] (Table 1). In legume–rhizobium symbiosis, [NiFe] hydrogenase catalyzes the oxidation of hydrogen into protons and electrons, playing an important role in the process of N fixation [128].

The sensitivity of enzymes to Ni effects in vivo may vary depending on the stage of plant development. For example, Ni inhibited the activity of the Calvin cycle enzymes (ribulose-1,5-bisphosphate carboxylase (oxygenase), 3-phosphoglycerate kinase, fructose-1,6-bisphosphatase, aldolase, NADP- and NAD-dependent phosphoglyceraldehyde dehydrogenases) in the leaves of *Cajanus cajan* when it was added to the nutrient solution at the early stage of vegetation (30 days after sowing seeds) (Table 1). However, at the same Ni concentrations tested, such effect was not observed when Ni was added to the nutrient solution at a later vegetative stage of plant development (70 days after sowing the seeds) [68]. The mechanism behind this phenomenon remains unexplored.

Thus, the activities of many enzymes are reduced in the presence of Ni, which largely determines its multiple effects on a wide range of cellular metabolic processes (Table 1, Figure 1). At the same time, the activities of some enzymes can increase in the presence of Ni, as it plays an important role in N metabolism, or this effect may also be associated with plant response to metal-induced oxidative stress. Further studies of Ni effects on enzyme activity in Ni-hyperaccumulating and non-accumulating species are necessary to elucidate whether the differences in metal tolerance and metal accumulation capacity are related to enzyme-specific responses to Ni in them.

## 3. The Effects of Ni on Antioxidant and Glyoxalase Systems

One of the main signs of metal toxic effects is ROS (O_2_^•−^, H_2_O_2_, OH^•^) production in cells, which leads to peroxidation of membrane lipids and protein degradation [92,116,129,130,131] (Figure 1). Although Ni is a non-redox-active metal, it can initiate oxidative stress in plants through ROS over-production (Table 2). The induction of ROS generation may be caused, in particular, by disrupting electron transport chains in chloroplasts and mitochondria through displacing Fe and Cu, thereby facilitating Fenton-like reactions [97,132] or by activating plasma-membrane NADPH oxidase [107,133]. In contrast to Ni, Fe can participate in direct electron transfer reactions and thus contribute to the generation of superoxide radicals (O_2_^•−^). In the Fenton reaction, Fe^2+^ is oxidized to Fe^3+^ by hydrogen peroxide, producing extremely reactive hydroxyl radicals. Thus, Ni-induced Fe accumulation can lead to oxidative stress [81]. In the roots and shoots of *Oryza sativa*, the generation of O_2_^•−^ and H_2_O_2_ increased with Ni concentration and duration of exposure [97,130,134]. A concentration-dependent increase in the content of H_2_O_2_ was typically observed in different species, e.g., in *Brassica juncea* at 50–150 μM Ni [115,116], *Gossypium hirsutum* at 50–100 μM Ni [135], *Triticum aestivum* at 100–200 μM Ni [136], and *Chenopodium quinoa* cultivars at 100–400 μM Ni [137]. Interestingly, at 150 and 300 μM Ni, a pronounced increase in H_2_O_2_ content in shoots was found in the non-accumulator *Aurinia saxatilis*, but not in the Ni hyperaccumulator *Odontarrhena inflata* [138] (Table 2).

The development of oxidative stress is largely caused by a metal-induced imbalance between the generation of toxic ROS and their scavenging through the antioxidative defense mechanisms. These mechanisms enroll an efficient system for ROS detoxification and scavenging involving the upregulation of antioxidant enzymes such as ascorbate peroxidase (APX), monodehydroascorbate reductase (MDHAR), dehydroascorbate reductase (DHAR), glutathione reductase (GR), glutathione peroxidase (GPX), glutathione S-transferase (GST), superoxide dismutase (SOD), peroxidase (POD), ascorbate oxidase (AO) and catalase (CAT) [105,111,113,116,134,171,177,178,179] (Table 3). SOD forms the key defense against O_2_^•−^, while APX, MDHAR, DHAR, and GR are crucial enzymatic components of the ascorbate–glutathione (AsA-GSH) cycle [113]. Nickel-induced increase in the levels of expression of *Fe-SOD* [106,117], *Cu*/*Zn-SOD* [78], *POD* [89,106,130,146], *APX* [78,106,130,146], *CAT* [89,117,130,146,180], *GR* [78,130], *GPX* [180], and *GST* [154] genes was often observed. However, a decrease in the expression levels of *SOD* genes was found in Ni-treated *Oryza sativa* and *Triticum aestivum* [130,180]. Interestingly, Ni treatment (50 µM) significantly increased the transcript abundance of *SOD*, *CAT*, *APX*, *GR*, *GST*, *MDHAR*, and *DHAR* genes compared to control plants, whereas the activities of only last two enzymes increased in the roots and leaves of *Solanum lycopersicum*, which markedly modulated the AsA-GSH pool [162]. The studies of Ni-induced changes in gene expression and activities of antioxidant enzymes indicated that Ni modulates the activities of antioxidant enzymes at both transcriptional and posttranscriptional levels [161].

The activity of enzymes may change depending on Ni concentration in the medium (Table 3) and duration of treatment [85,105,149,153,157,161,171,186,189,194]. At lower Ni concentrations, SOD activity in the leaves of *Triticum aestivum* and *Zea mays* increased, whereas at higher concentrations it decreased [194]. The inhibition of SOD activity might be related to Ni-induced deficiency of metals which are cofactors used by respective forms of this enzyme and/or to its inactivation by H_2_O_2_ or other ROS [129]. The activities of AO, CAT and POD in *Oryza sativa* seedlings were the highest at 50 μM NiCl_2_, but significantly decreased at higher Ni concentration. It is plausible that Ni did not directly affect the enzymes, as at the same concentrations, Ni did not significantly inhibit their activities in vitro [105]. The activity of GPX in the leaves of *Triticum aestivum* increased after 3 days of treatment with 100 μM Ni, but did not differ from that in control plants after 6 and 9 days of treatment [169]. The activity of CAT in the leaves of *Vigna radiata* increased at 10 μM Ni but decreased at 200 μM Ni after 4 days of treatment, whereas after 12 days of treatment, Ni inhibited CAT activity at both concentrations tested [171]. The decreased activity of CAT may result from enzyme deactivation due to direct interaction with metal ions or ROS, or from decreased biosynthesis of CAT protein or impaired protein assembly [134]. Overall, whether the activities of antioxidant enzymes are enhanced or suppressed in the presence of Ni may depend on Ni concentration, plant species, organ or tissue under study (Table 1). Intraspecific differences at the population or variety level may also be observed [97]. The decrease in the activities of antioxidant enzymes at high Ni levels leads to the enhancement of oxidative stress in plants.

The diverse response of the activities of different antioxidant enzymes may be observed even at the same Ni concentration in the medium (Table 3). For example, in seven-day-old *Zea mays* at 250 µM Ni, the activities of SOD, POD, DHAR, and GR were unaffected, whereas the activities of CAT, APX and MDHAR were stimulated [187]. At the same Ni concentration in the medium, the activities of SOD, MDHAR, DHAR, APX, GR, and GPX in the leaves of *Oryza sativa* increased, whereas the activities of GST and CAT decreased, albeit not significantly in case of CAT [111]. At 200 and 400 μM Ni, the activities of all isoforms of superoxide dismutase, i.e., Cu-Zn SOD, Mn-SOD and Fe-SOD, increased in the roots and shoots of *Oryza sativa* [134]. In different plant species grown under similar conditions of Ni-induced stress the activities of antioxidant enzymes may change to a different degree (Table 3), which reflects the differences in their Ni tolerance [195]. Differences at the population level were also identified [196].

Nickel effects on the activities of antioxidant enzymes may also differ in plant organs, which results from different Ni contents and constitutive levels of enzyme activity therein. For example, constitutive activities of SOD, APX, POD and GST were higher in the roots than in the shoots of *Triticum aestivum*. More prominent Ni-induced decrease in SOD activity was observed in the roots compared to the shoots, whereas the activity of CAT decreased in the shoots but did not change in the roots. The activities of APX, POD and GST increased several times in the shoots, but did not change significantly in the roots [136]. The activities of POD and SOD increased in the leaves, but decreased in the roots of Ni-treated *Amaranthus paniculatus*, whereas the activities of APX and CAT decreased in the roots but did not change in the leaves compared to those in control plants, which may be partially related to higher Ni accumulation in the roots of this species [86]. The activity of CAT decreased in the shoots, but increased in the roots of Ni-treated *Solanum nigrum*, while the activity of APX increased in the shoots, but did not change in the roots [167]. At 10 μM Ni, the activity of SOD decreased and the activity of POD increased in the shoots, whereas the activities of these enzymes in the roots were not altered. At higher Ni concentrations, similar changes in the activities of enzymes were observed both in roots and shoots of *Oryza sativa* [129]. In the roots as well as first and second fully developed leaves of Ni-treated (100 µM) *Zea mays*, the activity of SOD increased and the activity of CAT decreased, while the activity of POD elevated in roots and in first leaves, but declined in second fully developed leaves, whereas the activity of APX did not change in first leaves, but decreased in the other organs [175]. At a Ni concentration of 10 μM, the activities of SOD and CAT decreased, and the activity of GST increased in the first leaf of *Cucumis sativus*, whereas the activities of these enzymes in the second leaf did not change. A decrease in the activity of APX was observed only in the second leaf, while in the first leaf it increased, which may be related to uneven distribution of Ni in plant [132]. As the activities of antioxidant enzymes in response to Ni-induced oxidative stress change depending on metal concentration, plant species and organ, this significantly complicates making a direct comparison of the data obtained in different works.

Nickel effects on the activities of antioxidant enzymes may differ in hyperaccumulators and excluders, and the data are sometimes contradictory. When non-hyperaccumulator *Lobularia maritima* (*Alyssum maritimum*) was grown on Ni-amended nutrient solution, the activities of SOD, APX, GR increased, whereas in the hyperaccumulator *Odontarrhena argentea* (*Alyssum argenteum*), they were lower, and the activity of SOD was significantly inhibited [179]. Thus, high Ni tolerance in *Odontarrhena argentea* may be determined by the efficiency of the mechanisms of Ni detoxification, thereby reducing the need for the activation of antioxidant enzymes. In another Ni hyperaccumulator *Odontarrhena inflata* (*Alyssum inflatum*), after the exposure to 350 μM Ni, the activities of CAT and APX decreased in the shoots [81], whereas the activities of CAT, APX, SOD and POD increased in the roots at 100–400 μM Ni [157]. These findings point to organ-specific response to Ni, which may be related to different levels of Ni accumulation in roots and shoots. Moreover, the activities of these enzymes changed to varying degrees in plants from metallicolous and non-metallicolous populations [157]. It was later shown that upon plant exposure to Ni (300 μM), the antioxidant enzyme activities, at least those of APX and CAT, but possibly also that of POD, were more upregulated in the hyperaccumulator *Odontarrhena inflata* than in the hon-accumulator *Aurinia saxatilis*, which could explain the absence of Ni-induced H_2_O_2_ accumulation in the former [138]. Taking into account different experimental conditions and thus impossibility of direct comparison of the data, it cannot be excluded that at least in *Odontarrhena inflata*, an enhanced enzymatic antioxidant capacity might represent an essential component of the Ni hypertolerance syndrome.

There is currently no sufficient evidence to suggest that the activation of antioxidant enzymes, particularly that of CAT, POD, and SOD, is due to direct metal effects. Furthermore, all of these enzymes are metalloenzymes, whose activity may decrease due to the displacement of the essential metal. The increase in stress enzyme activity is likely to be induced by oxidative stress and is associated with the rise in ROS levels. The tolerance of particular enzymes and the activation of enzymatic systems responsible for stress metabolism may be considered as a possible mechanism of plant tolerance to metal excess.

During the evolution, plants have developed several mechanisms to alleviate oxidative stress. Besides antioxidant enzymes, ROS-scavenging machinery involves non-enzymatic antioxidants, such as ascorbate, glutathione, polyamines, carotenoids, phenols, and flavonoids, including anthocyanins. Enhanced accumulation of ascorbate and/or glutathione through the ascorbate–glutathione cycle is often observed in response to Ni. However, in some cases, treatment with Ni induced a decrease in ascorbate content (Table 4). Comparison of three species from the Asteraceae family, Cichorioideae subfamily, revealed different responses to Ni excess even among closely related species. Only one of the tested species, *Leontodon hispidus*, reacted by a decrease in ascorbate and thiols in its roots and shoots, which was accompanied by an increase in ROS, especially in the parenchyma of the vascular tissue and in the root cortex [197].

Glutathion is present in nearly all cell compartments, is a strong reducing agent and is easily oxidized, participating in many processes, including metal/metalloid binding, ROS inactivation, and regulation of redox homeostatic processes [204]. The ratio of oxidized and reduced forms of glutathione is an indicator of redox balance, whose maintenance at a certain level is crucial for plant survival [205]. Treatment of plants with Ni can cause both an increase and a decrease in the level of glutathione (Table 4). Glutathione is a precursor of phytochelatins, which play an important role in plant metal tolerance and detoxification by chelating metals [204]. Although Ni has a higher affinity for N-containing ligands than for S-containing ligands and the role of phytochelatins in Ni detoxification appears to be insignificant, Ni-stressed plants exhibit phytochelatin biosynthesis [154], which may affect cellular glutathione levels.

The Ni-induced changes in the contents of putrescine (PUT), spermidine (SPD) and spermine (SPM) in the roots and leaves of *Amaranthus paniculatus* are associated with antioxidative, membrane-protective, metal-chelating and antisenescent properties of these polyamines. When plants were exposed to moderate Ni concentration, free SPD and SPM contributed to alleviation of Ni toxicity. Moreover, polyamine catabolic enzyme was characterized by a marked sensitivity to Ni accumulation in plant tissues [86]. The increase in SPD, SPM and, most notably, PUT (by 4.5 times compared to control) in *Brassica napus* at 250 μM Ni [141] is consistent with the increase in the contents of these polyamines in *Solanum melongena* grown on Ni-contaminated soil (100 mg kg^−1^) [166]. Prominent increase in the level of PUT was also shown for the leaves of *Hydrocharis dubia*, though the contents of SPD and SPM decreased in the presence of Ni [87]. The increase in the contents of polyamines may promote Ni detoxification through the formation of Ni complexes with polyamines [141]. A similar role in Ni binding is played by such ligands as histidine, nicotianamine, organic acids and, to a lesser degree, phytochelatins [27,31,204,206,207,208].

Plant secondary metabolites such as phenolics and flavonoids are also known as essential bioactive phytochemicals with marked radical scavenging and metal-chelating properties [60,209]. Nickel-induced increase in the contents of phenolics and/or flavonoids was shown for a wide range of plant species (Table 4). A decrease in the contents of soluble phenols was rarely observed, such as, for example, in *Fagopyrum esculentum* at 5 mM foliar-applied Ni, which was probably due to a very high metal concentration [210]. The decrease in the contents of phenols and flavonoids was also shown for *Vinca rosea* at 50–100 mg kg^−1^ Ni in soil [172] and that of flavonoids also in *Brassica napus* at the same Ni content in soil [142] (Table 4). The contents of soluble phenols positively correlated with the contents of hydrogen peroxide, which pointed to their antioxidant potential [48].

Anthocyanins are among the most widespread flavonoids in plants. In the cell, they are predominantly accumulated in the vacuole, quickly react with oxidative stress products and are important components of the antioxidant system involving polyphenols [60]. The contents of anthocyanins increased in various plant species exposed to Ni [84,89,97,145,162,199]; although in the leaves of *Brassica napus*, a Ni-induced decrease in their contents was observed [142] (Table 4). The biosynthesis of flavonoids is catalyzed by a number of enzymes, including chalcone synthetase (CHS), chalcone isomerase (CHI), flavanone 3-hydroxylase (F3H), flavonoid 3’-hydroxylase (F3’H), dihydroflavonol 4-reductase (DFR), and flavonol synthase (FLS) [211]. In *Arabidopsis thaliana*, TRANSPARENT TESTA8 (TT8) is an essential basic helix-loop-helix (bHLH) transcription factor involved in anthocyanin biosynthesis which positively regulates the genes encoding the enzymes of flavonoid biosynthesis (*CHS*, *CHI*, *F3H*, *F3’H*, *DFR*, and *FLS*). As a transcription factor, TT8, together with the above mentioned genes, was upregulated by Ni treatment, suggesting that they are components of transcriptional regulation cascades which are activated in response to Ni stress. When TT8 was overexpressed in *Arabidopsis thaliana*, these plants accumulated more polyphenols compared to wild type plants and were able to tolerate extreme Ni stress showing high survival and germination rates [211]. It is believed that the biosynthesis of anthocyanins can be activated by the accumulation of such photolytic metabolites as superoxide anions, hydrogen peroxide, and singlet oxygen, suggesting that their biosynthesis is a non-specific mechanism of plant adaptation to high metal levels [60]. Acting as chelators of metal ions in plants, polyphenolic compounds may decrease the presence of free radicals and minimize oxidative stress in plants treated with metals at high concentrations [60,78]. Besides anthocyanins, the contents of other antioxidant pigments, i.e., β-carotene, β-cyanin and β-xanthin, also increased upon plant exposure to Ni [97].

Besides ROS generation, a major consequence of Ni toxic effects is the formation of cytotoxic glycolytic byproduct, *α*, *β*-dicarbonyl aldehyde compound, named methylglyoxal (Table 2). In plants, this highly reactive compound is produced in large amounts in response to abiotic stress and can induce ultrastructural damage, mutations, inactivation of DNA and proteins. Methylglyoxal exerts detrimental effects by generating ROS, forming advanced glycation end products and inactivating antioxidant systems, and thus is a biomarker for plant stress tolerance [212,213].

Methylglyoxal detoxification system is primarily driven by the action of two enzymes, glyoxalase I and glyoxalase II (Table 1), while glutathione acts as a cofactor [214]. In some species, Ni is a cofactor of glyoxalase I [215], whose activity increases upon exposure to Ni at low concentrations [88,111,113,114,115,116,117], whereas at high Ni concentrations, the activities of both glyoxalases may decrease [110] (Table 1). The mechanisms of metal effects on both antioxidant and glyoxalase systems, which have strong interactions in conferring abiotic stress tolerance in plants [214], are currently being actively studied.

## 4. The Effects of Ni on the Structure and Functioning of Membranes

The plasma membrane of root cells is the first barrier for metal entry and also a target for their toxic effects [208,216]. Metal-induced generation of ROS can lead to permanent damage to membranes through the oxidation of, and cross-linking with, protein thiols, inhibition of membrane proteins, and altering the composition of membrane lipids due to lipid peroxidation, particularly through interaction with polyunsaturated fatty acids (FAs) [217]. Overall, this leads to the destruction of cell membrane integrity, fluidity and functioning (Figure 1). Increased activity of lipoxygenase under Ni-induced stress (Table 1) enhances lipid peroxidation [72,97,108,109,110,111,112,113,114], which is followed by the increase in the concentration of malondialdehyde (MDA), a biomarker of oxidative stress in a wide range of species (Table 2). The formation of various odd-carbon metabolites, including MDA, results from the biodegradation of polyunsaturated FAs under oxidative stress [218]. However, due to the high antioxidant capacity and enhanced biosynthesis of antioxidant enzymes preventing the oxidative damage, Ni-induced reduction in MDA content has been shown in some works [140,165,183,195]. The effect of Ni on MDA content depends not only on the plant species and metal concentration, but can also be population-specific as a result of interpopulation differences in metal tolerance, as was shown, for example, for *Silene paradoxa* [196].

Metal-induced stress and lipid peroxidation are observed at higher metal levels in the medium in hyperaccumulators compared to excluders. Eight-day treatment with 100 μM Ni did not induce any significant increase in total lipid peroxidation in the shoots of the Ni hyperaccumulator *Noccaea goesingensis*, whereas in the non-accumulator *Arabidopsis thaliana*, lipid peroxidation increased by 800%, which reflects different Ni tolerance of these species [219]. However, in the Zn hyperaccumulator, *Arabidopsis halleri*, which does not hyperaccumulate Ni and is not Ni tolerant, the enhancement of MDA level in the shoots at 5 and 50 μM Ni compared to control was much stronger than that in the closely-related excluder *Arabidopsis lyrata.* At the same time, MDA level in the roots of both species increased similarly at 50 μM Ni [220].

Besides MDA, a significant increase in the contents of early produced oxylipins, most pronounced for 9-hydroxyoctadecatrienoic acid and 13-hydroxyoctadecatrienoic acid, was shown in *Cucumis sativus* in response to Ni. It was suggested that they might act as signaling molecules mediating plant response to metal stress [132].

Changes in the composition and structure of membranes can lead to changes in their fluidity and permeability, which in turn affects ion transport and the activity of membrane-associated enzymes and thus plays an important role in plant metal tolerance [221]. Membrane permeability is largely determined by the unsaturation of FAs that make up membrane lipids. The ability of plants to adjust membrane fluidity by changing the level of FA unsaturation is an important strategy for plant stress adaptation. Thus, changes in FA composition can be used as a marker for the action of various stress factors [222]. It is generally accepted that the unsaturation of membrane lipids increases in response to salinity and pathogens, but decreases in response to metals [222]. The most prominent Ni-induced changes in the FA composition were observed in the shoots of the Zn hyperaccumulator *Arabidopsis halleri* and in the roots of the excluder *Arabidopsis lyrata* (Table 5), although in both species, Ni accumulated predominantly in roots [220], and their tolerance to Ni was comparable [40]. In the shoots of *Arabidopsis halleri*, the contents of saturated FAs increased and the contents of unsaturated FAs decreased, whereas an opposite pattern was observed in the roots of *Arabidopsis lyrata* (Table 5), which was largely determined by the changes in the activity of FA desaturases [220]. The observed Ni-induced changes in the FA composition in the shoots of *Arabidopsis halleri* may result in a decrease in membrane fluidity, which leads to a decrease in ROS penetration into the membrane, thus maintaining its stability [220]. Exposure of *Cucumis sativus* seedlings to 10 µM Ni induced an increase in the level of phospholipid unsaturation in leaves, primarily due to a significant increase in the relative contents of oleic and linoleic acids, which was considered a stress reaction [132]. At the same time, exposure of *Triticum aestivum* seedlings to 50 and 100 µM Ni altered the total FA composition, leading to a decrease in their unsaturation. In both roots and shoots, there was a significant decrease in the relative content of linolenic acid, whereas in shoots, a parallel increase in the relative content of palmitic acid and a decrease in the relative content of palmitoleic acid were also observed [168]. Fatty acid profiles in the fronds of Ni-treated *Lemna minor* were also characterized by an increase in the relative contents of saturated FAs and a decrease in the relative contents of unsaturated FAs, mainly associated with the conversion from linolenic and linoleic acids to palmitic acid [183]. It is not yet clear whether the observed reduction in unsaturation level is a defense mechanism or rather a consequence of the metal-induced oxidative damage. The degree of FA unsaturation changes differently depending on the nature of the stress factor, which does not promote co-tolerance to various stressors [222], as well as depending on plant species [132,168,220]. To understand the reasons for the identified contradictions, further research is needed, including the studies of Ni-induced changes in the FA profiles in Ni hyperaccumulators.

It was shown that Ni treatment alters not only the FA composition but also the ratio of lipids of different classes. For example, Ni treatment induced changes in the sterol and phospholipid composition of the plasma membrane of *Oryza sativa* shoot cells [65]. In the leaves of *Triticum aestivum*, Ni induced a significant increase in the relative content of phosphatidylethanolamine (PE) but not that of phosphatidylcholine (PC) [223], whereas in the leaves of *Cucumis sativus*, the relative content of these two most abundant phospholipid classes remained unchanged [132]. Since PC and PE phospholipids differ in the shape of their molecules and thus have opposite effects on membrane stability, changes in the PC/PE ratio may significantly affect membrane fluidity and, consequently, permeability. In the leaves of Ni-treated *Cucumis sativus* seedlings, the PC/PE ratio did not change significantly [132], whereas in the leaves of *Triticum aestivum*, Ni induced a decrease in the PC/PE ratio suggesting an increase in membrane permeability [223]. Nouairi et al. [224] suggested that the change in the PC/PE ratio may be related to plant sensitivity to metal effects. However, further studies are needed to prove that these changes are related to plant Ni tolerance. The relative contents of phosphatidic acid and phosphatidylglycerol increased and that of phosphatidylinositol decreased in Ni-treated plants, although the observed changes were restricted to only one of the tested leaves, either the 1st or the 2nd, depending on the phospholipid class [132]. Cooke and Burden [225] considered metal-induced changes in membrane composition as one of the mechanisms regulating the activity of membrane ATPases, which create the proton gradient that is necessary for transmembrane transport in plants. Changes in the activity of membrane ATPases under the influence of Ni [62,63,65,76] may partially explain the change in membrane permeability for various substances and ions [226], which affects the ion balance in the cytoplasm.

Peroxidation of unsaturated FAs in membrane lipids as a result of metal-induced accumulation of ROS and increased peroxidase and lipoxygenase activity leads to numerous cascade reactions that damage membranes, leading to a loss of their integrity, and, as a consequence, increase in their permeability to ions [222,227]. When a cell dies and loses the integrity of its membrane, electrolytes, such as ions of K^+^, which is the most mobile element transported across the plant cell membrane, leak out of the cell. Nickel-induced increase in electrolyte leakage was observed in many species (Table 2). Thus, maintaining membrane integrity under the influence of metals is a key factor determining plant metal tolerance [228]. Changes in the structure of plant cell membranes may be one of the causes of Ni-induced disturbance of mineral nutrition in plants.

## 5. The Effects of Ni on Mineral Nutrition

The toxic effects of metals on plants can be associated with disruptions in the uptake and transport of nutrients, leading to the changes in their contents in plant organs. Metals influence the uptake of other ions through various mechanisms, the relative contribution of which varies depending on metal concentration, physicochemical properties of its ions, as well as plant species and stage of development.

In most cases, Ni inhibits the absorption and/or transport of both cations (K^+^, Ca^2+^, Mg^2+^, Mn^2+^, Zn^2+^, Cu^2+^, Fe^3+^) (Table 6), and anions (NO_3_^−^) [90,94,95,102]. As all these nutrients are involved in a number of physiological processes, their deficiency in plants may disrupt plant metabolism. In addition, some metals, such as Fe, Cu, Zn and Mn, are integral components of prosthetic groups of a number of metalloenzymes, such as SOD and CAT. Impaired biosynthesis of metalloenzymes may result from metal-induced mineral deficiency.

Metals influence the uptake of other ions through different mechanisms, the relative importance of which varies in different cases. Nickel-induced reduction in the uptake and/or translocation of macro- and microelements may result from the proximity of their ionic radii (e.g., Ni^2+^, Mg^2+^, Fe^2+^, Zn^2+^ have ionic radii of 78, 78, 82 and 83 p.m., respectively [243]) [1,3,47]. It has also been reported that Ni has similar characteristics (mass to charge ratio) to those of other nutrients such as Ca, Mg, Mn, Fe, Cu and Zn [229].

Metal transport into the cytosol is mediated by different proteins which are to a varying degree non-selective. This leads to competition between metal ions during their transport across the plasma membrane [31,244,245]. In non-graminaceous species, ferric iron (Fe^3+^) is converted to ferrous (Fe^2+^) iron by ferric chelate reductase, and Fe^2+^ is taken up into root cells, for example, by non-selective IRT1 transporter (iron-regulated transporter 1) from ZIP (ZRT/IRT-like proteins) family [31,244,246,247,248]. Being located on the plasma membrane, IRT1 can participate in the transport of Fe, Zn, Co, Ni, Mn, and Cd into the cytoplasm [249,250,251,252,253,254,255,256]. The expression of the *IRT1* gene is regulated by various transcription factors, such as bHLH Ivc, bHLH Ib (basic helix–loop–helix), and FIT (Fe-deficiency-induced transcription factor), depending on the Fe content in the medium [217,257]. Nickel downregulated the expression of *bHLH100-like*, *FRO1/2*, and *IRT1* in roots [161]. Therefore, in the presence of excess Ni, Fe content is often decreased (Table 6). At the same time, after treatment with Ni, the expression levels of the Zn transporter *ZIP10* and the Fe transporter *IRT1* were increased in the hyperaccumulator *Noccaea caerulescens* [233], and the expression of *IRT1*, *FRO2*, and *FIT* was enhanced in the roots of the non-accumulator *Arabidopsis thaliana* [254]. This phenomenon can be attributed to Ni-induced Fe deficiency, potentially triggering the expression of the *IRT1* gene associated with the Strategy I (reducing) of Fe homeostasis [233,253,254]. Nickel exposure stimulated the expression of genes encoding Zn and Fe transporters in *Odontarrhena chalcidica* [258], which confirms the involvement of Zn transporters in the transport of Ni in Ni hyperaccumulators. IREG2 (iron-regulated protein) is involved in Ni transport into the vacuole [252,259], with high constitutive level of *NcIREG2* expression also being detected in the leaves of *Noccaea caerulescens* [260]. Nickel root-to-shoot translocation in the Ni hyperaccumulator *Leucocroton havanensis* can be mediated by IREG1, which may also be involved in metal sequestration in cell walls [259].

The expression of these and other genes encoding metal transporters can vary depending on metal concentration, plant species and population, plant age, and the stage of development of organs and tissues [261,262,263]. This may largely explain the differences in the effect of Ni on Zn accumulation in different species and at different metal concentrations. For example, in the presence of Ni, a decrease in the Zn content was observed in the roots of *Glycine max* [92], in the leaves of *Helianthus annuus* [229], shoots of *Lolium perenne* [232], roots and shoots of *Hordeum vulgare* [230], *Zea mays* [173], and in a Zn-efficient cultivar of *Triticum aestivum* [264]. The Zn content did not change in *Phaseolus vulgaris* [235] and *Bornmuellera emarginata* [8], whereas in a Zn-inefficient cultivar of *Triticum aestivum* [264] and in the leaves of *Hibiscus sabdariffa* [199], it increased. The inhibitory effect of Zn on Ni accumulation was shown in both excluders and hyperaccumulators [264,265,266,267,268]. The non-specificity of transporters is one of the reasons why the accumulation of metals under their combined treatments, which is most often observed under natural conditions, differs from their accumulation under the separate treatments. In addition to competition for transporters, competition between elements is also possible during Ni entry through non-selective cation channels [269,270,271]. Increased expression of the *NtCBP4* gene (a homolog of *AtCNGC1*), encoding NtCBP4 located on the plasma membrane, promoted enhanced Ni tolerance, which is associated with a decrease in Ni accumulation and confirms the possible involvement of CNGC (cyclic nucleotide gated channel) in Ni transport [272]. However, Ni-specific transporters or channels have not been recognized to date. The involvement of non-specific transporters and channels in Ni transport and search for ones with high affinity for Ni in Ni hyperaccumulating species is one of the promising areas for future research.

Metal-induced disruption of cellular metabolism, resulting in altered membrane enzyme activity and structural membrane reorganization, also affects the transport of essential nutrient elements. For example, Ni-induced reduction in Fe uptake by roots may be associated with decreased activity of ferric-chelate reductase, which was shown for *Amaranthus paniculatus* [79] and *Cucumis sativus* [80] (Table 1).

Nitrogen is one of the macronutrients essential for plant metabolism and is an integral part of basic N-containing compounds such as nucleic acids and proteins. Ammonium (NH_4_^+^) and nitrate (NO_3_^−^) are the most common inorganic forms of N, making up most of the available N taken up by plants [94,248]. The uptake of NO_3_^−^ usually decreased in response to Ni treatment [90,94]. On the contrary, the absorption of NH_4_^+^ may increase upon Ni treatment, which, in turn, may exert toxic effects on various physiological processes [94,99].

The ionic balance under the influence of Ni changes differently in different plant organs, and, possibly, even tissues [119,162,230,239] (Table 6). For example, during the tillering phase, the contents of Fe, Mn and Zn decreased in the leaves of *Triticum aestivum*, whereas only the content of Mn decreased in the roots [239]. At 10 and 100 μM Ni, the contents of Fe and Cu increased in the roots of *Hordeum vulgare*, but decreased in the shoots, whereas the contents of Mn and Zn lowered in both organs [230].

The effect of Ni on the accumulation of other elements largely depends on its concentration in the nutrient solution or content in soil [92,230,232,233,264,273]. For example, at low Ni level in the soil, the Fe content in the shoots of *Lolium perenne* increased, whereas at high Ni level, it decreased [232] (Table 6). Similarly, when the Ni content in the soil increased from 50 to 200 mg kg^−1^, the contents of Cu and Mg in the grains and the contents of Mg and Ca in the stems of *Triticum aestivum* decreased [239]. Nickel concentration-dependent accumulation of Ca, Mg, Cu, and Mn in grains, shells, and shoots was also demonstrated for *T. aestivum* grown in sierozems [273]. The content of macronutrients in the roots and shoots of this species depended not only on the Ni concentration in the medium, but also on the concentration of sulphur [274].

Analyzing the data presented in Table 6, it can be concluded that at high Ni concentrations (about 10^−4^–10^−3^ M), the contents of macro- and microelements in plant organs usually decrease due to the inhibition of both their absorption and transport [178,234]. At the same time, at low Ni concentrations in the medium, in some cases, there was an increase in the contents of nutrients or their contents in plant organs did not change [120,199,230,235,239] (Table 6). On the one hand, the increase in the contents of essential elements under the influence of Ni can be accompanied by stimulation of plant growth. For example, this was shown for *Hordeum vulgare* grown at 1 μM Ni, which accumulated higher amounts of Cu, Fe, Mn, and Zn [230]. On the other hand, the Ni-induced increase in the contents of mineral elements may be a consequence of the “concentration effect”. This means that when the growth of Ni-exposed plants is inhibited, but the rate of metal uptake is not changed yet compared to control, the metal content per unit mass increases [235].

Metals affect not only the ion uptake but also their transport across plant tissues, which results in differential changes in the contents of various mineral elements in plant organs (Table 6). For example, after 14 days of incubation of *Hordeum vulgare*, the root-to-shoot translocation of Cu and Fe was repressed, while that of Mn and Zn was not decreased with increasing Ni concentration in the nutrient solution. As a result, the contents of Cu and Fe increased in roots and decreased in shoots, whereas the contents of Mn and Zn diminished in both organs [230]. The Ni-induced increase in Fe content in roots and decrease in its content in shoots have also been shown for other plant species [162,242].

When different species are treated with metal salts at the same concentrations, their effects on plants may vary. For example, when *Triticum aestivum* and *Triticum durum* were treated with 67 μM Ni, the contents of Ca and Mg in the leaves of *Triticum aestivum* increased and the content of Zn decreased, whereas in *Triticum durum*, the contents of these elements did not change [239] (Table 6). Under identical conditions, Ni treatment inhibited both the uptake and root-to-shoot translocation of Ca, Mg, Zn, and Cu, but only the translocation of Fe and Mn in *Trifolium repens*, while in *Zea mays*, Ni repressed the absorption of Cu, Ca, and Mg, and the translocation of Zn, Cu, Fe, Mn, Ca, and Mg. In *Lolium perenne*, the translocation of Cu, Fe, Mn, Ca, and Mg was decreased, but uptake of these elements, except that of Mg, was not affected by Ni [275].

Nickel-tolerant and Ni-sensitive plants may differ in the pattern of changes in mineral composition in response to Ni. When two *Triticum aestivum* cultivars were grown on Ni-contaminated soil (50–200 mg kg^−1^ soil), the Fe content in grain decreased in both cultivars, while the contents of Cu, Ca, and Mg decreased only in the non-tolerant cultivar. Furthermore, the latter also exhibited a deficiency of Mn and Mg in leaves, which resulted in leaf chlorosis [239]. Nutrient- and cultivar-specific patterns of nutrient accumulation under Ni stress were also found by other researchers in *Helianthus annuus* [229] and *Triticum aestivum* [276]. Overall, specific changes in the mineral nutrition of metal-tolerant excluder plants require further studies.

Few studies have been devoted to the Ni-induced changes in mineral composition of hyperaccumulator plants [81,233,277,278]. In the Ni hyperaccumulator *Odontarrhena muralis*, the Fe contents in roots and stems, and the Mg contents in roots and leaves increased with Ni concentration in the medium [277]. Exposure of *Odontarrhena inflata* to high Ni concentrations resulted in the inhibition of root-to-shoot translocation of Fe and concentration-dependent progressive Fe accumulation in the pericycle, endodermis, and cortex in the root differentiation zone. The disruption of root-to-shoot Fe translocation was suggested to be the major cause of Ni toxicity symptoms in this species [81]. In Ni-treated *Noccaea caerulescens*, the total uptake of Fe increased, whereas that of Mn and Zn decreased [233]. However, excess Ni did not induce Fe deficiency in the serpentine population of *Noccaea caerulescens*, Monte Prinzera, as it did in Ni-sensitive species. Instead, Ni competed with Fe during translocation rather than uptake [278].

The influence of one metal on the accumulation of another can vary not only between species but also between plants from different populations of the same species. This has been studied in detail for Ni and Zn in hyperaccumulators. Nickel did not significantly affect Zn uptake or translocation in *Odontarrhena corsica* and inhibited only the translocation of Zn in plants of different populations of *Noccaea caerulescens*. At the same time, Zn inhibited both the translocation and the uptake of Ni in *Odontarrhena corsica*, and only the uptake, but not the translocation of Ni in *Noccaea caerulescens* [268]. Zinc uptake was not influenced by Ni, but Ni uptake was severely suppressed by Zn in the Ni hyperaccumulating *Odontarrhena chalcidica* [258]. Similarly, Ni exposure had no discernible effect on Zn accumulation in *Bornmuellera emarginata* [8], which aligns with the observations for the three populations of *Noccaea tymphaea* (formerly *Thlaspi pindicum*), in which no significant decrease in Zn accumulation was reported, and in plants of one population, there was even an increase in the Zn content. The presence of Zn, in contrast, led to a decrease in Ni accumulation in plants of all studied populations [266]. It is likely that Ni is transport is carried out via low-affinity transporters of other divalent micronutrients, whereas Zn is transported via high-affinity transporters, making its accumulation less susceptible to variations in Ni concentration in the nutrient solution [265]. However, for *Odontarrhena corsica*, Mohtadi and Schat [44] later obtained data that seem to indicate against Zn and Ni uptake via a common transporter, except for the case when it would have high affinity for Ni and low affinity for Zn. On the other hand, this would contradict the fact that Zn inhibited Ni uptake, but not vice versa [268]. In this regard, it is important to mention that Ni uptake mechanisms in Odontarrhena seem to be polyphyletic [42], and are highly variable [271]. Thus, as the populations of *Odontarrhena corsica* used in the works by Mohseni et al. [271] and Kozhevnikova et al. [268] are of a very distant geographic origin, this could explain different patterns observed for metal uptake. It is also plausible that Ni hyperaccumulator and non-hyperaccumulator serpentinophytes differ in their Ni root-to-shoot translocation capacities rather than Ni uptake capacities [271]. In general, the changes in the mineral profile under the influence of Ni are often both species- and population-specific and may to a certain degree lie behind different metal tolerance capacities of different species and populations.

To summarize the abovementioned, it can be concluded that there are various mechanisms of Ni effects on the uptake, transport, and distribution of nutrients within plants, which are dependent on numerous internal and external factors, and require further research and clarification. Metal-induced disruption of mineral nutrition may be one of the indirect mechanisms of Ni toxic effects on different metabolic processes (Figure 1), ultimately leading to growth inhibition, disruption of morphogenesis, and reduced plant yield.

## 6. The Effects of Ni on Transpiration and Water Balance

Maintaining water balance is determined by the ratio of water absorption and transpiration rates. Many researchers have noted a decrease in stomatal conductance and/or transpiration, as well as a reduction in water content in plants under the influence of Ni [64,68,90,92,116,139,144,161,162,163,172,178,179,185,190,198,236,241,279,280]. A decrease in leaf water potential, stomatal conductance, transpiration rate, and total water content was observed in *Triticum aestivum* on the fourth day of treatment with 10 mM Ni. These changes were most pronounced in the uppermost leaf, in which the greatest amount of Ni was accumulated [279]. At high Ni concentrations in the medium, a decrease in stomatal conductance and transpiration rate was observed in *Cajanus cajan* [68], *Capsicum annuum* [144], *Citrullus lanatus* [146], *Eucalyptus urophylla* [150], *Glycine max* [92,117,151,152], *Gossypium hirsutum* [135], *Ipomoea batatas* [153], *Solanum lycopersicum* [72,113,162,163,164], *Solanum melongena* [166], *Vigna radiata* [73,241], *Vinca rosea* [172], and *Zea mays* [176]. The decrease in stomatal conductance was also detected in *Brassica juncea* [66,67,88,116], *Catharanthus roseus* [70], *Chenopodium quinoa* [137], *Populus nigra* [281], and the decrease in transpiration rate was also shown for *Amaranthus paniculatus* [86], *Brassica oleracea* [190], *Brassica juncea* [115], *Cicer arietinum* [89], and *Triticum aestivum* [100]. The Ni-induced disturbance of water regime in plants was manifested in a decrease in water and turgor potentials, as well as relative water content (RWC), and was noted in different non-hyperaccumulator species [69,97,110,111,114,115,116,117,136,145,148,149,152,153,159,166,171,175,178,279,282,283,284,285,286,287] and some hyperaccumulators [8,288]. The decrease in RWC might be related to disturbances in root growth, transpiration, stomatal movement, and root hydraulic conductivity [1]. However, the changes in RWC, transpiration rate and stomatal conductance depend on Ni concentration in the medium and may increase at low Ni concentrations, which is consistent with the growth stimulation observed in *Ipomoea batatas* [153] and *Vigna unguiculata* [102]. The Ni-induced increase in stomatal conductance and transpiration in the Ni hyperaccumulator *Odontarrhena chalcidica* is a rarely observed phenomenon, which was not found in the other two closely related species [288]. The enhancement of transpiration rate was also detected in the hyperaccumulators *Noccaea caerulescens* and *Arabidopsis halleri* treated with Zn [289,290]. Significant increase in transpiration rate, but not in stomatal conductance, was shown for the non-hyperaccumulator *Helianthus annuus* at 10–40 mg L^−1^ Ni [285].

The mechanisms of Ni effects on transpiration rate may be associated with a decrease in leaf size or in the size of intercellular spaces, changes in the stomatal density, hydropassive closure of stomata due to the loss of turgor or ABA-dependent hydroactive metal-induced closure of stomata, depending on the Ni concentration and duration of exposure [291].

Metal-induced growth inhibition leads to a decrease in the size of leaves, which are the major transpiring organs. Such a decrease in the leaf blade area (by 40%) was observed in *Cajanus cajan* grown in sand culture irrigated with nutrient solution amended with 1 mM NiCl_2_, which led to a decrease in transpiration rate [68]. The decrease in leaf blade area was also shown for *Brassica oleracea* grown on agar medium in the presence of 5–20 g m^−3^ NiSO_4_ [280], hydroponically grown *Brassica juncea* at 150 µM Ni [69], *Gossypium hirsutum* at 50 and 100 µM Ni [135], *Ipomoea batatas* at 30 or 60 mg L^−1^ NiCl_2_ [153], *Phaseolus vulgaris* at 100–500 µM Ni [292], *Pisum sativum* [159], *Solanum lycopersicum* [161] and young *Zea mays* at 100 µM Ni [175], in first trifoliate leaves of *Vigna radiata* plants incubated in nutrient solution containing 10–1000 µM NiCl_2_ [284] or grown in soil amended with 100, 200 mg kg^−1^ NiCl_2_ [241], in *Oryza sativa* grown at 80, 100 mg kg^−1^ Ni in soil [97], *Catharanthus roseus* at 50–150 mg kg^−1^ Ni in soil [70], *Ocimum basilicum* at 100, 150 mg kg^−1^ Ni [156], as well as in *Brassica juncea* [66] and *Glycine max* [152] at 200 mg kg^−1^ Ni in soil. In Ni-treated *Brassica juncea*, *Gossypium hirsutum*, *Ipomoea batatas*, *Ocimum basilicum*, *Vinca rosea* and *Zea mays*, there was a reduction in the number of leaves [135,153,156,172,293,294], whereas in *Amaranthus paniculatus* and *Salix viminalis* there was a decrease in total leaf area [201,295]. The inhibition of transpiration resulted from Ni-induced reduction in the number of stomata per unit leaf area in *Brassica oleracea* [280], *Eucalyptus urophylla* [150], *Glycine max* [296], *Pisum sativum* [159] and *Solanum lycopersicum* [164]. The decrease in stomatal density is often accompanied by an increase in the equatorial diameter of the stomata and a decrease in stomatal index. These parameters are variables intrinsically linked to the quantity, size and functionality of the stomata and are important indicators used to assess stomatal behavior under stress conditions [150,164,296]. However, metal-induced increase in stomatal density may result not from enhanced stomata formation, but rather from the reduction in cell size due to metal-induced water deficit and inhibition of cell elongation [291], or from stronger inhibition of leaf growth compared to that of differentiation of stomatal guard cells [297].

Metal exposure can lead to elevation of the abscisic acid (ABA) level [279], which, along with the decrease in RWC and cell turgidity, stimulates stomatal closure. As a mechanism for water loss prevention, Ni-induced stomatal closure was shown, for example, in *Phaseolus vulgaris* [298] and *Solanum lycopersicum* [237]. The experiments conducted on epidermal preparations of *Helianthus annuus* and *Zea mays*, floating on the surface of Ni, Pb, Cd and thallium (Tl) solutions [299,300] showed that these metals can directly induce stomatal closure. Moreover, damaged and permanently closed stomata were found in Ni-treated *Brassica oleracea* [280], whereas in the leaves of *Solanum lycopersicum*, scanning electron microscope micrographs showed distortion of stomata and reduction in their length [237]. By influencing ABA metabolism, metals stimulate the expression of *ltp* genes in the epidermis, responsible for the biosynthesis of non-specific lipid-transfer proteins, which causes an increase in the number of monomers entering the site of cutin biosynthesis and, consequently, an increase in the thickness of the cuticle, which leads to a reduction in transpiration [301]. Ultramorphological analysis of *Solanum lycopersicum* leaves indicated a Ni-induced increase in the thickness of the wax layer of the cuticle [237]. Impaired respiration and oxidative phosphorylation can also reduce the water-holding capacity of cells and disrupt the water regime of plants.

Metal treatments often induce an increase in the contents of substances involved in turgor maintenance and a decrease in cell wall plasticity [47,297,302,303], which leads to a decrease in water potential [285]. Enhancement of callose deposition in the cell walls of root cells, which is often observed upon treatment with Ni and other metals, may reduce the rate of water movement through the apoplast [291,304]. The decrease in water potential and cell wall elasticity may be one of the mechanisms of growth inhibition. Reduction in root length, root damage and metal-induced changes in root architecture, in turn, limit the flow of water into the plant [291]. The increase in the degree of vacuolization of root rhizodermal and cortical cells in *Psidium guajava* [236] and enhanced water retention therein promoted protecting them from significant perturbations in metabolite concentrations in the cytosol [291].

Thus, metals can reduce or block water transport from roots to shoots through multiple mechanisms, from the effects on stomatal opening and closure to the effects on water permeability of root cells [291,302,305,306]. In addition to disrupting the ratio of water absorption to transpiration [291,302], metal-induced water deficiency can be associated with ROS production and membrane lipid peroxidation [307]. The latter was observed in the presence of Ni in both hyperaccumulators and excluders [220]. The decrease in water content can cause plasmolysis and lead to plant wilting. Overall, metal-induced changes in water regime are one of the main causes of their toxic effects.

Nickel-induced water deficiency often triggered a significant increase in the content of proline [152,180,193,195,283,308] (Table 4), which resulted from induced activities of γ-glutamyl kinase, pyrroline-5-carboxylate synthetase, and pyrroline-5-carboxylate reductase, and inhibited activity of proline oxidase (Table 1), playing an imperative role in controlling the level of proline [88,89]. The increased proline content in plants under Ni toxicity may also result from the hydrolysis of proteins due to oxidative stress as well as the inhibition of proline degradation [152]. In contrast, in the Ni hyperaccumulator *Bornmuellera emarginata*, an intriguing Ni-induced decrease in proline content was observed [8] (Table 4). Free proline acts as an osmoprotectant, regulating water balance, as a signalling molecule, and is involved in stabilizing macromolecules, ROS scavenging, and protecting cells from stress-induced oxidative damage [111,309,310]. The ability of proline to chelate metals was also confirmed [310,311]. The content of proline often increases with Ni concentration in the medium [71,97,111,115,130,139,144,153,165,308]. Accumulation of proline has been frequently used as a biochemical marker for water stress in plants.

Glycine betaine is another key player in plant abiotic stress tolerance. Its content in plants is also often increased in response to Ni toxicity. It plays multiple roles in osmotic adjustment, maintaining membrane integrity and protecting protein structures from damage induced by abiotic stress [78,97,100,106,113,115,116,159]. In Ni-treated *Oryza sativa*, the decrease in the relative water content in leaves was accompanied by a parallel rise in the accumulation of osmotic cytosolutes, such as proline and glycine betaine, as well as total free amino acids, soluble proteins, and sugars [97]. The rise in the contents of free amino acids and/or soluble sugars (carbohydrates) upon Ni treatment was shown for many plant species [82,89,100,103,144,148,153,241]. The accumulation of osmotic cytosolutes under metal-induced stress conditions promotes the regulation of cellular water balance and preserves membrane integrity through scavenging ROS and chelating excessive metal ions. These osmolytes significantly decrease the solute and water potential and ensure plant survival under Ni stress.

Boyd and Martens [312] suggested that hyperaccumulators may be characterized by increased tolerance to water deficit. This assumption was confirmed by the absence of any decrease in water contents in the roots and shoots of *Odontarrhena montana* (*Alyssum montanum*) treated with 1000 mg kg^−1^ Ni [313]. In *Odontarrhena chalcidica* and *Odontarrhena muralis* (*Alyssum murale*), the tissue hydration state also remained stable under Ni treatment, whereas the leaves of *Odontarrhena moravensis* exposed to 0.25 mM NiSO_4_ even showed an increased hydration state [288]. Nickel accumulation in leaf epidermal cells in the hyperaccumulator *Hybanthus floribundus* was suggested to promote reduced cuticular transpiration [314] or increased osmolality in the cell [315]. The increase in Ni accumulation in the shoots of the Ni hyperaccumulator *Stackhousia tryonii* with the decrease in the humidity level is consistent with the data indicating that Ni may play an osmoprotective role along with proline, glycine betaine or mannitol [316]. Accroding to the hypothesis stating that metal hyperaccumulation promotes plant protection from drought stress [17,312], Ni that is accumulated in the vacuoles of water storage cells of leaf epidermis in different Ni hyperaccumulator species [31,316,317,318] may act as an osmotic in these compartments [316].

Elevated organic acid content in the shoots of *Noccaea caerulescens* and other hyperaccumulators summarized in the reviews [31,207] may also contribute to the decrease in osmotic potential [319]. A positive correlation between the total free amino acids and leaf osmotic potential was found in *Brassica napus* [282]. However, Whiting et al. [320] showed that Ni accumulation in *Odontarrhena muralis* (*Alyssum murale*) did not significantly affect the osmolality of leaf-sap extracts, RWC in shoots, or transpiration rate. Ni also had no effect on the rate of water loss in *Odontarrhena muralis* and *Noccaea caerulescens* either in the absence of water deficit or under moderate (−0.4 MPa) water stress induced by aqueous polyethylene glycol [320]. It is evident that Ni content in plant tissues cannot change as rapidly as water content therein. Therefore, Ni accumulation in shoots can hardly be considered as an important osmoregulatory mechanism. Although some hyperaccumulators are more tolerant to water deficit, this is probably not determined by their ability to accumulate metals in shoots. Thus, the drought tolerance hypothesis of hyperaccumulation remains controversial and requires further research.

## 7. The Effects of Ni on Photosynthesis

**Figure 2:**
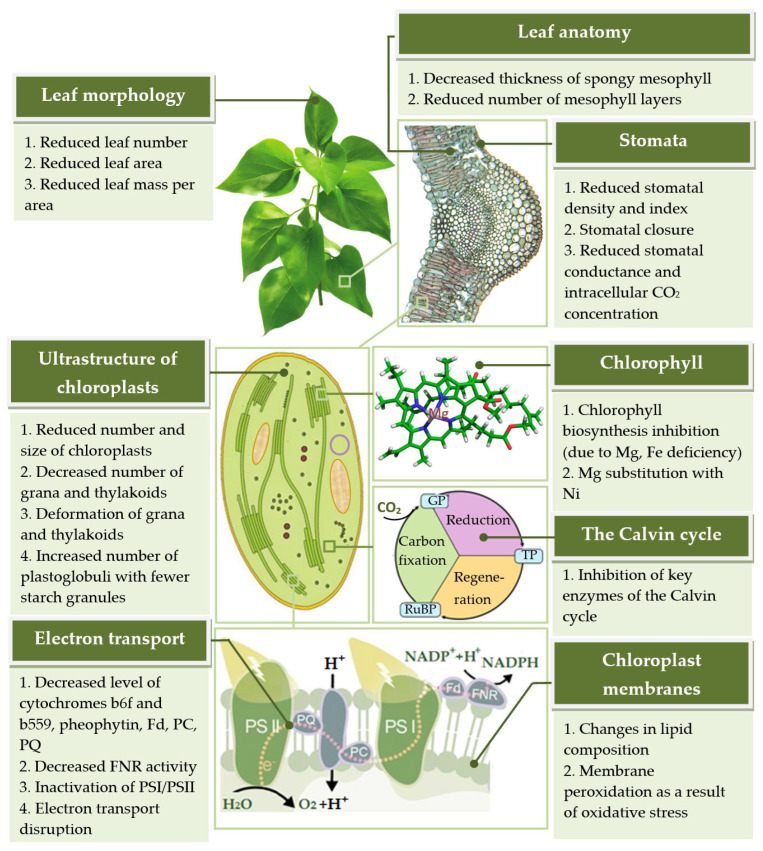
The toxic effects of Ni on the efficiency of photosynthesis. Fd—ferredoxin; FNR—ferredoxin-NADP(H) oxidoreductase; GP—glycerate-3-phosphate; PC—plastocyanin; PSI—photosystem I; PSII—photosystem II; PQ—plastoquinone; RuBP—ribulose bisphosphate; TP—triose phosphate.

The effects of Ni on photosynthesis has been studied in detail, which made it possible to identify both the direct influence of Ni on individual reactions of photosynthesis, and the indirect influence due to the disruption of other processes, such as, for example, mineral nutrition. The reduction in overall intensity of photosynthesis may be related to Ni-induced decrease in leaf area and leaf number, disruption of the ultrastructure of chloroplasts, inhibition of the biosynthesis of chlorophylls, plastoquinone and carotenoids, disruption of electron transport, inhibition of enzymes of the Calvin cycle, and reduced leaf internal CO_2_ concentration due to stomatal closure related to low RWC and cell turgidity. Some authors have reported significant decreases in the stomatal (e.g., net photosynthetic rate (*P*_n_), intracellular CO_2_ concentration (*C*_i_), stomatal conductance (*g*_s_), and transpiration rate (*E*)) and non-stomatal (e.g., PSII activity (*F*v/*F*m), photochemical quenching (qP), and electron transport rate (ETR) or maximum light-driven electron transport rate (*J*_max_)) attributes of photosynthesis due to Ni toxicity [66,73,113,281] (Table 7, Figure 1 and Figure 2).

Nickel-induced stress causes significant structural and functional alterations to chloroplasts, thus impairing photosynthetic efficiency [322] (Figure 1 and Figure 2). Reduced number and size of chloroplasts and their ultrastructural disorganization, along with the changes in membrane lipid composition, were reported for Ni-treated plants [5,94,146,280,323]. For example, decreased size and number of chloroplasts as well as disruption of their ultrastructure (decrease in the number of grana and thylakoids and their deformation, formation of plastoglobuli, changes in the lipid composition of membranes) were found in *Brassica oleracea* grown on agar medium amended with 10–20 g m^−3^ NiSO_4_. These changes might be related to Ni-induced decrease in water content in cells or oxidative stress, which resulted in lipid peroxidation [280]. Ultrastructural changes in the chloroplasts in *Brassica oleracea* were also detected under the excess of organic Ni(II) complexes [324]. The chloroplasts of Ni-treated (200 μM) seedlings of *Oryza sativa* were characterized by swelled shape, destroyed thylakoid system, increased number of plastoglobuli with fewer starch granules as compared with control [94]. Amoeboid-shaped chloroplasts with severely damaged thylakoid membranes and abundant starch granules were found in Ni-stressed leaves of *Solanum lycopersicum* [237]. Nickel-induced induction of transition from chloroplasts to chloro-amyloplasts and amylo-chloroplasts or even amyloplasts with some stroma and a rudimentary thylakoid system was observed in *Spirodela* and *Lemna* [323].

A typical consequence of the toxic effects of Ni is a decrease in the contents of photosynthetic pigments: chlorophylls [70,71,74,79,81,82,95,97,103,108,110,111,115,130,137,142,143,145,146,149,152,154,155,165,167,175,178,180,186,191,192,195,198,202,283,286,287,292,293,308,323,325,326,327] and carotenoids [70,71,79,82,97,103,115,130,142,145,146,155,163,171,186,198,202,283,286,292,294,325], which are responsible for the photochemistry, absorption and transfer of solar energy to the thylakoid membranes. Some works showed a stronger decrease in the content of chlorophyll *b* compared to that of chlorophyll *a* [137,142,144,146,149,150,154,159,163,164,166,172,176,185,202,237,238,286,328]. However, in many other studies, an opposite pattern was observed [82,145,171,190,284,323], which may partly be related to the duration of incubation [328], as well as the concentration of Ni [71,130,149,191,283] and Fe in the medium [79]. Despite the fact that Ni-induced changes in the contents of individual photosynthetic pigments may vary, the content of carotenoids was usually more tolerant to Ni excess compared to the content of chlorophylls. Thus, the changes in chlorophyll fluorescence can be considered a suitable and sensitive indicator of metal toxic effects [48,327]. A comprehensive analysis of a large number of works revealed a positive correlation between chlorophyll content and changes in shoot dry weight [48].

Metal-induced decrease in the content of functional chlorophyll may be determined by several mechanisms (Figure 2). One of them is the substitution of Mg^2+^ in the chlorophyll molecule with other metals including Ni^2+^ in vivo. At the same time, Ni-containing chlorophyll is so stable that it does not bleach, even after weeks of exposure to direct sunlight. This substitution prevents photosynthetic light-harvesting in the affected chlorophyll molecules, resulting in a breakdown of photosynthesis [329,330]. The decrease in chlorophyll content may also be related to metal-induced inhibition of the enzymes of chlorophyll biosynthesis such as δ-aminolevulinic acid synthase, δ-aminolevulinic acid dehydratase, protochlorophyllide reductase, and porphobilinogenase [280,331,332,333,334], stimulation of chlorophyll degrading enzyme chlorophyllase demonstrated in vitro [335], Ni-induced Fe and Mg deficiency [161,232,235,238] (Table 6), as well as production of H_2_O_2_ resulting in chloroplast membrane peroxidation, which may be one of the major reasons for decreased chlorophyll content under Ni stress [115,336]. Indeed, Ni treatment of *Solanum lycopersicum* significantly reduced the contents of intermediates of the chlorophyll biosynthesis pathway, such as glutamate 1-semialdehyde, δ-amino levulinic acid, prototoporphyrin IX, Mg–prototoporphyrin IX, and protochlorophyllide [113].

The changes in chlorophyll content may differ between species at the same metal concentration in the environment, which reflects their different tolerance. Therefore, chlorophyll content is often considered an important biomarker for a variety of abiotic stresses, including metals. The decrease in chlorophyll content under the influence of Ni is often visually expressed as chlorosis, with yellowing between veins, and is sometimes followed by necrosis [68,71,87,92,108,144,165,171,192,201,230,232,235,238,239,241,279,280,283,337,338]. In Ni hyperaccumulators, the contents of photosynthetic pigments often did not change or decreased slightly even at high metal concentrations [8,288]. Therefore, signs of chlorosis appear in these species at significantly higher Ni concentrations compared to excluders.

In chloroplasts isolated from *Hordeum vulgare*, Ni was shown to inactivate the activity of photosystem II (PSII) at a lower concentration than required for the same degree of inhibition of photosystem I (PSI)-mediated electron flow [339]. Nickel-induced decreases in Φ_PSII_, electron transport rate, and photochemical quenching (qP) were shown for many plant species (Table 7, Figure 1 and Figure 2). This affected initial (*F*_0_) and maximal (*Fm*) fluorescence yield and demonstrated worse absorption, photon capture, and maintenance of QA oxidation, as well as had a negative effect on the flow of electrons through PSII. Nickel excess also induced Ca release from the oxygen-evolving complex of PSII [340]. Nickel-induced impairment of electron transport, associated primarily with PSII [106,292,341,342,343] may be a consequence of structural and functional changes in thylakoid membranes, as well as a result of a decrease in ferredoxin-NADP^+^ oxidoreductase activity and impaired biosynthesis of plastoquinone [343]. Nickel reduced the efficiency of the electron flow from pheophytin via plastoquinone *Q*_A_ and Fe to plastoquinone *Q*_B_, changing the structure of carriers, such as *Q*_B_, or proteins of the reaction center present in the thylakoids [342,343]. Nickel-mediated concentration-dependent decrease in pheophytin content was shown in *Raphanus sativus* seedlings [191]. In addition, under the influence of Ni, the levels of cytochromes b_6_f and b_559_, as well as those of ferredoxin and plastocyanin in chloroplast thylakoids, can decrease, which leads to a decrease in the efficiency of electron transport in *Ocimum basilicum* [341].

In Ni-treated plants, the maximum photochemical efficiency of PSII (*F*v/*F*m) was often significantly reduced, pointing to the inhibition of light absorption and energy accumulation in the antenna complex and the development of favourable conditions for ROS overproduction, which can inflict considerable damage to thylakoid structures and pigments [151]. Nickel toxicity caused a discernible reduction in *F*v/*F*m and qP with a simultaneous increase in non-photochemical quenching (NPQ) values in two cultivars of *Oryza sativa* grown at 80 or 100 mg kg^−1^ Ni in soil [97]. Similar changes in chlorophyll fluorescence parameters were previously documented in different plant species under Ni-induced stress [66,67,78,86,113,144,161,164] (Table 7). The decline in *F*v/*F*m [78,86,113,150,151,162,164,281] may be connected with chlorophyll degradation, while reduced qP values indicate structural and functional impairment of PS II reaction centers [97]. The rise in NPQ under Ni toxicity reflects the enhanced dissipation of excess excitation energy as heat, which functions as a protective mechanism against PSII photodamage caused by disrupted electron transport and oxidative stress [78,97]. At the same time, in wild and cultivated *Carthamus* species, Ni toxicity affected the acceptor side of PSI and its components to a greater degree than the donor side of PSII [145]. Moreover, Ni treatment also decreased the maximum quantum yield of primary photochemistry and variable fluorescence in *Triticum aestivum* [238] and diminished chlorophyll *a* fluorescence induction kinetics in *Zea mays* [328]. Detailed studies conducted on Ni-treated *Lolium perenne* seedlings indicated that Ni reduced the activities of PSII and PSI. This may result from an increase in nonradiative dissipation of PSII antenna chlorophylls, a decrease in PSII antenna size or/and a decrease in the number of photosynthetic apparatuses with fully closed PSII reaction centers. The electron transport chain efficiency was reduced due to the loss of PSII activity and the impairment of PSI functioning [327].

The effect of Ni on photosynthetic efficiency depends on leaf age. In *Populus nigra*, Ni-induced changes were more pronounced in developing leaves than in mature leaves, partly due to higher Ni accumulation therein [281]. The pronounced Ni-induced decline in mesophyll conductance resulting in a dramatic decrease in CO_2_ concentration at the carboxylation site, especially in developing leaves of *Populus nigra*, was regarded as a major limitation to photosynthesis. The strong decline in electron transport rate and maximum carboxylation velocity with increasing Ni levels also indicated major biochemical limitations to the net photosynthetic rate. Those impairments were stronger in developing leaves than in mature ones. The significant Ni-induced increase in initial fluorescence, especially in developing leaves, pointed to severely altered efficiency of energy transfer from the PSII light-harvesting complex to the PSII reaction center. A pronounced negative effect of Ni on the water-splitting site of PSII was also demonstrated in developing leaves [281]. The decrease in net photosynthesis, intercellular CO_2_ concentration and water use efficiency was also shown in Ni-stressed *Brassica juncea* [116] and other plant species (Table 7).

Metals also exert toxic effects on dark reactions of photosynthetic processes by inhibiting the activity of key enzymes of the Calvin cycle (Figure 1 and Figure 2), which was observed in the leaves of *Cajanus cajan* incubated in the presence of NiCl_2_ (1 mM) [68] and *Brassica juncea* grown at 200 mg Ni kg^−1^ soil for 15 days [66] or 30 days [67] (Table 1). It is plausible that inhibition of the Calvin cycle reactions, being the primary mechanism of metal effect, leads to the accumulation of adenosine triphosphate (ATP) and nicotinamide adenine dinucleotide phosphate (NADPH), formed as a result of light reactions, which, in turn, create a high pH gradient across the thylakoid membrane, which inhibits the fuctioning of PSII [343]. Deficiency of either N or S also may cause a significant reduction in the photosynthetic efficiency in plants [66].

In the Ni hyperaccumulator *Odontarrhena muralis* cultivated in soil mixed with sewage sludge, even at high Ni contents in the medium (1568 mg kg^−1^) and leaves (up to 12,730 mg kg^−1^) the functioning of the photosynthetic machinery was not affected [277]. However, when grown hydroponically at 0.25 and 1 mM Ni, the non-serpentine population of *Odontarrhena muralis* displayed a decrease in photosynthetic performance at the lowest Ni concentration due to a combination of both stomatal and non-stomatal limitations. Intriguingly, in contrast to *Odontarrhena muralis*, in two other Ni hyperaccumulators from serpentine soils, namely *Odontarrhena chalcidica* and, to a lesser extent, *Odontarrhena moravensis*, Ni treatments increased not only the photochemical efficiency of PSII and the CO_2_ assimilation rate, but also CO_2_ diffusion from the atmosphere to the carboxylation sites (Table 7). Together with stable chlorophyll and carotenoid levels in these species, these findings clearly indicate a stimulatory, hormetic-like effect of Ni on both the biophysics and biochemistry of photosynthesis in these Ni-hyperaccumulating species [288]. Roccotiello et al. [321] reported negligible effects of Ni on chlorophyll fluorescence parameters and photosynthetic efficiency in *Alyssoides utriculata* at Ni contents in shoots slightly above the hyperaccumulation threshold limit (1000 μg g^−1^) (Table 7). Similar results supporting the lack of negative effects of high soil Ni contents on photosynthetic performance in *Alyssoides utriculata* were obtained by Rosatto et al. [313], who also observed a similar response in the Ni-hyperaccumulator *Noccaea caerulescens*. Taken together, these data indicate greater photosynthetic tolerance to Ni in hyperaccumulators compared to excluders due to the existence of effective mechanisms for avoiding severe damage to photosynthetic apparatus based on metal chelation, sequestration, and detoxification [31,204,206,207]. In non-accumulating species, a hormetic-like response pattern and associated increase in the contents of chlorophyll and total pheophytin, as well as increase in photosynthetic rate and electron transport rate were sometimes observed at low Ni concentrations [102,120].

Thus, Ni can affect photosynthesis, as well as other processes, directly and indirectly. The direct effect is associated, for example, with the inhibition of enzymes involved in chlorophyll biosynthesis and the Calvin cycle, as well as with disruption of electron transport due to a decrease in the plastoquinone pool and the activity of ferredoxin NADPH^+^ oxidoreductase. The indirect effect is mediated by metal-induced water stress, Mg and Fe deficiencies, stomatal closure, which reduces the amount of available CO_2_, as well as the modification of several leaf morphological traits, such as leaf blade area and mass per area (Figure 2). Changes in the contents of photoassimilates in the leaves of Ni-treated plants can result in further changes in the rate of photosynthesis and, as a consequence, in the sink/source balance and energy metabolism within a plant [201].

## 8. The Effects of Ni on Respiration and ATP Content

The effect of Ni on respiration and ATP biosynthesis has not received much attention and remains poorly studied. An increase in the number of mitochondria and peroxisomes was observed in the root cells of Ni-treated *Solanum lycopersicum*. Furthermore, in Ni-exposed plants, mitochondria had degenerated cristae [237]. In *Pisum sativum* grown hydroponically at low Ni concentrations (10^−7^–10^−6^ M), the intensity of respiration in roots and shoots increased, whereas at higher Ni concentrations, it was inhibited [6]. A significant decrease in the respiration rate was observed in 25-day-old maize seedlings exposed to 170 μM Ni. Short-term exposure (2 and 4 h) had no effect on the respiration rate of excised roots of 4-day-old seedlings, and a decrease in the respiration rate was observed only after 18 h of exposure [344]. Furthermore, since Ni affects the metabolism of organic acids [207], it can also affect respiration indirectly. The Ni-induced inhibition of respiration may be one of the reasons for its indirect effect on a wide variety of physiological processes (Figure 1) and, therefore, requires further studies. All of the above-mentioned Ni-induced changes in cellular metabolism lead to significant disruption of plant growth and development and decline in their productivity.

## 9. The Effects of Ni on Plant Growth and Morphogenesis

**Figure 3:**
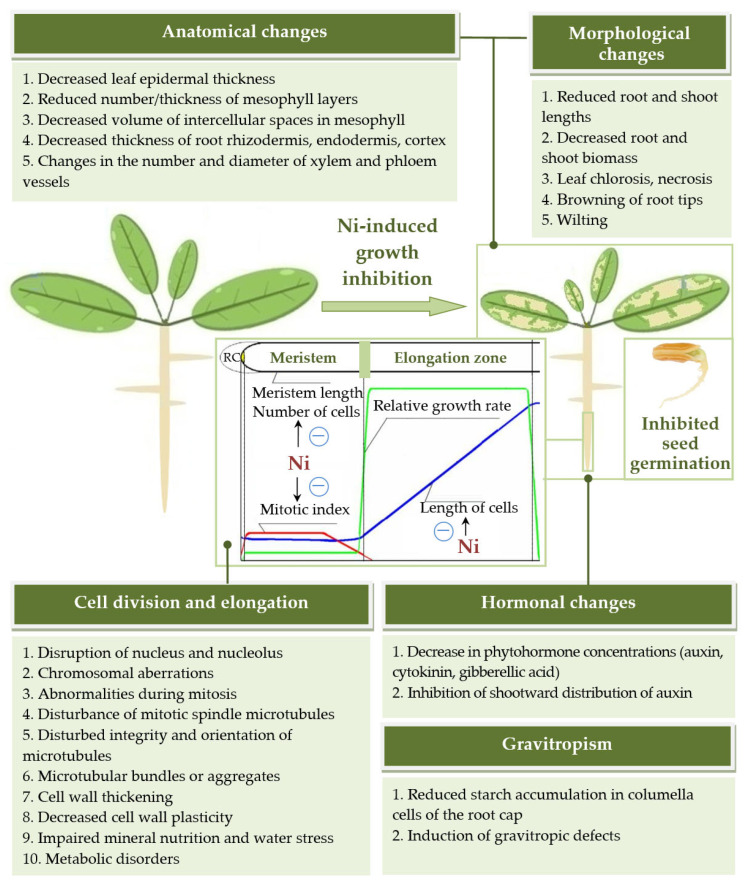
Toxic effects of Ni on plant growth and morphogenesis. RC—root cap.

**Figure 4:**
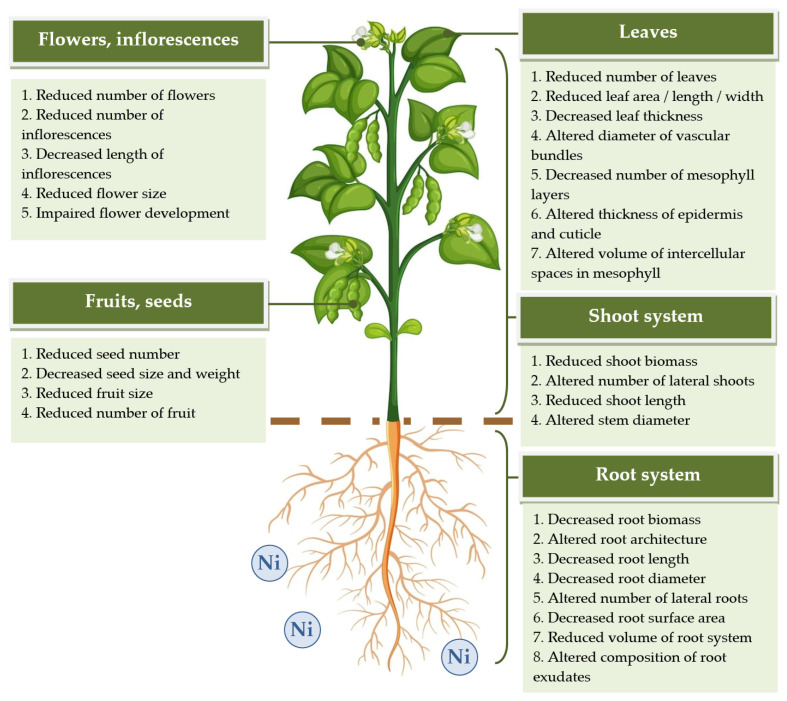
Toxic effects of Ni at the whole plant level.

Impairment of plant morphogenesis is one of the visible symptoms of the effects of various stress factors, which is often used to assess their phytotoxicity. The toxic effects of metals result in growth inhibition, which is widely used to test for their presence in the environment [297,345,346,347,348,349,350,351,352,353,354]. The Ni-induced growth inhibition can be assessed via different plant growth parameters and is usually manifested as a decrese in root and/or shoot lengths as well as fresh and/or dry biomass [71,78,95,100,147,158,190,286,293,355,356,357] (Table 8), which is considered one of the key indicators of toxicity. At high Ni concentrations in the medium, such visible symptoms of toxicity as wilting, browning of root tips and broken off roots were also observed [283] (Table 8, Figure 3 and Figure 4). The number of nodules, their fresh and dry mass, the content of leghemoglobin, the contents of N and carbohydrate in the nodules decreased in Ni-treated *Vigna radiata* [74], whereas in Ni-treated *Oryza sativa*, there was a reduction in culm length, panicle length, number of tillers per plant, and number of productive tillers per plant [286].

Different salts of the same metal at the same cationic concentration may exhibit different toxicities to plants. When Ni was added to the soil, its toxicity for *Zea mays* seedlings increased depending on the selected anionic partner in the following order: NiSO_4_ < Ni(CH_3_COO)_2_ < Ni(II)-citrate < NiCl_2_ < Ni(II)-EDTA (ethylenediaminetetraacetate) [355]. Therefore, not only different growth conditions, but also the usage of different Ni salts may complicate comparing the data obtained in different works.

Niethammer [363] was among the first researchers to notice the influence of Ni on seed germination (Figure 3). He found that at the lower concentration (0.1% solution), Ni had a stimulating effect, whereas at the higher ones (0.5 to 1.0%), it inhibited germination. Further studies also showed stimulatory effects of Ni at low concentrations on the germination of *Calendula tripterocarpa* seeds [143] and *Oryza sativa* caryopses [105]. When supplied in excess, Ni inhibited seed germination in *Brassica juncea* [140,293], *Cajanus cajan* [182], *Calendula tripterocarpa* [143], *Raphanus sativus* [191], *Spinacia oleracea* [359], *Vigna radiata* [106] and germination of caryopses in many Poaceae species [78,195,364,365]. Nickel toxic effects on seed germination and seedling growth is related to the disruption of metabolic processes, decrease in cell wall elasticity and inhibition of cell division [1,47]. Germination energy, germination percentage, germination and vigor indices decreased, and mean germination time significantly increased in Ni-treated *Glycine max* [117]. In part, the dual effect of Ni on seed germination may be determined by the stimulation of hydrolytic enzymes mobilizing the storage nutrients from the endosperm to the developing embryo at the lower concentrations of Ni and the decrease in their activity at the higher Ni concentrations [366]. Plant treatment with Ni also affected the seed weight and number, which determines productivity in many crops. At 50–150 mg Ni kg^−1^ soil, the number of seeds per pod, seed yield per plant and 100-seed mass decreased in *Vigna radiata* [73,74]. The decrease in the weight of 100 grains was observed in *Triticum aestivum* at the same Ni content in soil [180], while the decline in seed yield in *Glycine max* was shown at 200 mg Ni kg^−1^ soil [152]. The reduction in the number of grains per panicle, filled grain percentage and 1000 grain weight was observed in Ni-treated *Oryza sativa* [286].

Most of the substances entering the plant are absorbed by the root system. Therefore, the primary plant response to these substances is usually observed in the root system (Figure 3 and Figure 4). The root is characterized by a relatively high growth rate, which is associated with high levels of metabolic activity, making it sensitive to various stress factors, including metals [367]. Hence, root growth has long been used to assess the toxic effects of metals and test for their presence in the environment [349,353].

Root growth is usually more sensitive to Ni than shoot growth [69,115,129,136,145,171,190,362,368,369,370]. This correlates with predominant accumulation of metals in the roots of excluders and their entry into the plant mainly through the root system.

The tolerance index, that is the ratio of the length of the root (or shoot) of a metal-treated plant to the length of the root (or shoot) of a control plant, expressed as a percentage, is used as a value characterizing plant metal tolerance and the degree of metal toxicity [369,370]. The toxic effects of metals are also often characterized using LC_50_, that is metal concentration in the medium inducing a 50% root growth inhibition [346,368,371,372]. This method is based on the results of root growth test. Wong and Bradshaw [368], using the LC_50_ data for *Lolium perenne*, ranged metals according to their toxic effects in the following descending order: Cu > Ni > Mn > Pb > Cd > Zn > Al > Hg > Cr > Fe. This points to a much higher Ni toxicity for this species compared to many other metals. For other plant species, the degrees of toxicity of different metals may differ [61,83,368,371,372,373,374,375] (Table 9), which may be associated with interspecific differences in plant metal tolerance. In some cases, it is also indicative to use a root test to determine the lethal concentration of metal at which root growth is completely arrested (EC_100_) [40].

Kopittke et al. [376] conducted a meta-analysis of the literature data and ranged metals from most to least toxic based on their median toxic concentrations in growth medium: Pb (0.30 μM) ≈ Hg (0.47 μM) > Cu (2.0 μM) > Cd (5.0 μM) ≈ arsenate [As(V)] (9.0 μM) > Co (17 μM) ≈ Ni (19 μM) ≈ Zn (25 μM) > Mn (46 μM). However, the concentrations of trace metals that are toxic to plants vary widely among different metals, plant species or genotypes, and experimental conditions [376]. Moreover, the degree of metal toxicity may differ within species depending on plant organ. For example, metal toxic effects on the total biomass and leaf biomass of *Hydrilla verticillata* increased in the order Pb < Ni < Cd, whereas the order of metal toxicity for the stems was reverse [83]. The presence of divalent competing cations such as Ca and Mg in the growth medium, the pH of the soil or solution, as well as the contents of clay and organic matter, redox potential and other soil properties may affect the availability of trace metals for plant uptake, and, therefore, the manifestation of their toxic effects [47,217].

Metal effect on plant growth depends on their concentration in the medium. At low Ni concentrations, plant growth may be stimulated. Growth stimulation in *Vigna radiata* at 1 μM Ni was accompanied by an increase in leaf area of first trifoliate leaves, RWC and chlorophyll content [284], whereas the root length of *Allium cepa* increased at 1–10 μM Ni [377]. At 10 μM Ni, the biomass of *Hordeum vulgare* significantly increased [230], whereas in *Triticum aestivum*, only a slight enhancement in shoot fresh weight was observed [185]. In one of the two cultivars of *Cucumis sativus*, a significant rise in shoot and root dry matter yield was observed at 50 μM Ni, although the concentration of Ni required for their optimal growth and yield varied among the cultivars [118]. An increase in plant height, number of leaves, RWC, root and shoot fresh and dry weights, root length, root volume, surface area, number of root forks, root tips, average root diameter, as well as photosynthesis and transpiration rates, stomatal conductance, chlorophyll and carotenoid contents was observed in *Ipomoea batatas* at 15 mg L^−1^ NiCl_2_ [153]. Low levels of Ni (10 mg L^−1^) also improved shoot growth in two *Helianthus annuus* cultivars (Hysun-33 and SF-187) grown in sand culture [285]. Fresh and dry weight, chlorophyll content and herbage yield in *Mentha arvensis* rose at 20 mg Ni kg^−1^ soil [71]. Increasing Ni content in soil from 25 to 50 mg kg^−1^ significantly enlarged plant height, number of branches, as well as fresh and dry weights of calyces in *Hibiscus sabdariffa* [199]. One of the possible mechanisms determining the stimulation of plant growth and development by Ni at low concentrations is the beneficial role of Ni in nitrogen-related metabolism and stress metabolism, since Ni is required for the activities of enzymes such as urease [122,123,124,125], RNAase [378], and glyoxalase-I [215], and the activity of various enzymes of N metabolism in plants is stimulated by Ni [98,102,120] (Table 1). Soil amendment with Ni at low concentrations resulted in the enhanced photosynthetic, urease, and nitrogenase activities, increased number of nodules and nodule dry weight, and consequently, root and shoot dry weights and grain yield, which can be used in fertilization practices [120].

Growth stimulation in hyperaccumulators is usually observed at much higher Ni concentrations than in excluders. There are many reports in which a Ni-stimulatory effect was observed in some Odontarrhena [43,277,320,379,380,381] and Noccaea [360,380] hyperaccumulators, although in other studies this stimulation was not observed for species from these genera [233,382,383,384]. The root and shoot growth of a facultative serpentinophyte *Odontarrhena sibirica*, the majority of whose populations were shown to lack Ni hyperaccumulation capacity, was stimulated at 50–150 and 50 μM Ni, respectively, whereas the root and shoot growth of the Ni hyperaccumulator *Odontarrhena chalcidica* was stimulated at 50–500 and 50–1000 μM Ni, correspondingly [385]. In six Central-Eastern Mediterranean Odontarrhena species, a stimulatory effect on growth estimated by root length increment was shown under the treatment with 50 to 500 μM Ni, depending on the species/population [43], which was quite high considering the low toxicity threshold of Ni, which is usually below 5 μM for non-tolerant crop species [386]. Interestingly, the most tolerant Odontarrhena species/populations required the highest Ni concentrations in the culture medium for optimal growth [43]. In hydroponically grown plants of Monte Prinzera population of *Noccaea caerulescens* from ultramafic soil in Italy, characterized by high Ni tolerance, an increase in biomass accumulation was observed at 100 μM Ni, whereas for the less Ni-tolerant populations La Calamine and Saint Félix de Palliéres from calamine soils and Lellingen population from non-metalliferous soils this effect was not observed [360]. However, *Odontarrhena chalcidica* from a non-serpentine site (Chalkidiki peninsula, Greece) showed similar Ni-induced growth stimulation and the same level of Ni tolerance as the conspecific serpentine populations [43]. Interestingly, the number of inflorescences per plant, the length of inflorescences, and the number of open flowers per inflorescence were all significantly greater in Ni-treated *Odontarrhena inflata* compared to Mg-treated or control plants. It was suggested that Ni-induced stimulation of flowering in *Odontarrhena inflata* could result from more efficient N metabolism in Ni-treated plants, promoting greater fitness for the species on serpentine soils [387]. However, Ni accumulation in the generative organs of Noccaea [388] and Odontarrhena [389] species can lead to possible negative effects on plant fertility as well as pollinator species.

Hyperaccumulators growing on Ni-enriched serpentine soils may require elevated soil Ni for optimal root growth. Statwick et al. [390] suggested that elements such as Ni, Se, and, possibly, Cd can benefit hyperaccumulators of these elements and enhance their growth despite the cost of uptake, proposing the term “elemental stimulation” for this phenomenon. In accordance with the “elemental stimulation” hypothesis, Boyd et al. [391] reported that the roots of the seedlings of the Ni hyperaccumulator *Streptanthus polygaloides* exhibited greater elongation when growing on serpentine soil compared to non-serpentine soil. When the seedlings of the hyperaccumulator *Streptanthus polygaloides* and non-hyperaccumulator *Streptanthus insignis* were grown in vertical agar-filled Petri dishes and placed on either side of a central filter paper strip soaked in either NiCl_2_ solution or deionized water, primary roots of the hyperaccumulator grew in the direction of Ni-soaked paper, and the number and lengths of lateral roots were significantly increased toward Ni-soaked filter paper only in the hyperaccumulator, which may enhance Ni uptake [11]. The term “nickelophilic root foraging” was suggested to be applied to this behavior. In general, elements are usually distributed quite heterogeneously both in natural and contaminated soils, suggesting that root foraging may be beneficial for enhancing metal uptake and accumulation by hyperaccumulator species [11]. Tognacchini et al. [392] observed root foraging towards Ni-enriched soil in the ultramafic accession of *Noccaea caerulescens* and avoidance of Ni in the metal-tolerant non-accumulator species, *Stellaria media*. The roots of the Ni hyperaccumulator *Berkheya coddii* did not forage towards the Ni-rich patches (soil spiked with 62 or 125 mg kg^−1^ Ni), but the presence of Ni in soil changed root morphology, as the plants produced thicker roots with reduced branching [393], which may be connected with Ni toxic effects, as root biomass decreased compared to control.

Higher concentrations of Ni have been shown to reduce vegetative growth, such as plant height and overall biomass production in many species [71,362,394] (Figure 4). When studying metal toxic effects, it is important to determine the minimum metal concentration at which root growth inhibition starts. For example, toxicity symptoms in *Glycine max* began to appear at 0.1 µM, when shoot Ni concentration reached 28.9 mg kg^−1^ [92]. The studies on several crop species showed that Ni content in plant tissues below 10 mg kg^−1^ did not lead to a decrease in their yield [394]. Moreover, for most plant species, Ni is required in minute quantities (0.01–10 μg g^−1^ dry weight) to complete their life cycle [13,52].

Unlike excluders, in hyperaccumulators, growth is inhibited at significantly higher Ni levels in the medium, indicating their higher tolerance and more efficient Ni detoxification mechanisms [40,360]. The results of a comparative analysis conducted under identical conditions showed that root growth in the hyperaccumulator *Noccaea caerulescens* was completely arrested at 650–1200 μM Ni, depending on the population, whereas in the closely related excluder *Thlaspi arvense* and in the Zn/Cd hyperaccumulator *Arabidopsis halleri* it was completely inhibited at 100 μM Ni, and in the excluder *Arabidopsis lyrata*, complete growth cessation was observed at 150 μM Ni in the nutrient solution [40]. The results of the root test showed that Ni tolerance in *Noccaea caerulescens* is species-wide, with differences in tolerance being smaller among populations from ultramafic soils than among populations from calamine and non-metalliferous soils. The most tolerant populations of *Noccaea caerulescens* tested were Cira and Puente Basadre, originating from ultramafic soils in Spain, in which complete arrest of root growth was observed at 1200 μM Ni [40].

Root branching occurs through the formation of lateral roots, which arise due to cell division in the pericycle above the elongation zone. The duration of lateral root development from the first cell divisions in pericycle to the emergence of lateral roots is often tolerant to metal effect [349]. Nickel did not inhibit the initiation of lateral root primordia in *Triticum aestivum* [395] and *Zea mays* [362], but the number of lateral roots decreased in the latter, possibly, as a result of Ni accumulation in the lateral root primordia [362]. Due to the inhibition of root growth, the region where primordia were initiated ‘moved’ closer to the root tip [395,396]. In contrast to these species, Ni markedly induced lateral root formation and the formation of brush-like lateral roots at the root tips of *Solanum lycopersicum* [161] and increased the density of lateral roots in *Arabidopsis thaliana* [131,358], although the number of primordia in the latter significantly decreased at 50 μM Ni [397]. This difference suggested that different response mechanisms may be involved in modulating the growth of root system in response to Ni toxicity in dicotyledonous and monocotyledonous species [161]. Like root growth, root branching in hyperaccumulators is more tolerant to the action of Ni, and in some populations of *Noccaea caerulescens*, it did not stop completely even at 1150 μM Ni [40].

Root architecture is altered as a result of Ni effects on root growth and branching (Figure 4). If metal concentrations are not excessively high, the number of lateral roots decreases to a smaller degree than the length of the main root, and the root system becomes more compact [297,348]. A detailed analysis of the changes in the root architecture of *Capsicum annuum*, *Ipomoea batatas*, *Solanum lycopersicum* and *Citrullus lanatus* with the use of WinRHIZO software (version 2003a, Regent Instruments, Canada) indicated a decrease in root length, root volume, surface area, the number of root forks, root tips, root crossings, average diameter, and projected area in Ni-treated plants [144,146,153,163]. It is evident that metal-induced impairment of root architecture may in turn restrict the ability of plants to absorb minerals and water, leading to overall plant growth reduction.

Root growth rate is determined by cell division and elongation (Figure 3). If a substance selectively inhibits cell division in the root, its effect on growth is always evident only some time after the start of incubation and increases significantly over time. Rapid inhibition of root growth indicates the effect of metals on cell elongation. If a substance does not selectively affect cell division or elongation, root growth inhibition will vary slightly over time [353,398]. Therefore, the analysis of root growth curves and changes in growth inhibitory effect over time allows us to suggest a possible mechanism for the toxic effect of a particular chemical compound. However, in order to reliably identify the selectivity of the effect of a substance or compound on growth, it is necessary to conduct a comprehensive integrative analysis of its effect on various processes, assessing root growth depending on the duration of plant treatment, the length of the meristem, the number of cells in the meristem, the mitotic index (MI) (the proportion of meristematic cells that proliferate), the lifespan of the cells in the meristem (from the moment of their formation to their exit from the meristem), the duration of the cell cycle, the relative rates of cell division and elongation, the length of fully elongated cells, cell viability, etc. [398,399,400].

Back in 1928, Hammett noted a decrease in the MI in the roots of onion and corn under the influence of Pb(NO_3_)_2_ and explained this by the binding of Pb to sulfhydryl groups of proteins and glutathione [401]. The Ni-induced decrease in the MI and inhibition of cell division was described for many plant species [352,353,395,396,402,403,404,405,406,407]. The decrease in the MI was also observed in the first three cell layers of the columella of the root cap in *Zea mays* [351]. The lowering of the MI in the root apex may result from the changes in the duration of the cell cycle, arrest of one or more mitotic phases, blocking of G2 phase in the cell cycle, preventing the cell from entering mitosis, as well as disturbances in the process of mitosis due to the inhibition of DNA biosynthesis, direct metal interaction with DNA, or general disturbance in the cellular metabolism [352,353,395,408]. The inhibition of cell division in *Plantago lanceolata* at higher Ni concentrations was synchronised with an elevated number of cells in early anaphase and a reduced the number of cells in prophase and metaphase [406]. The inhibition of cell division in the meristem is accompanied by a decrease in its length, which was shown for the roots of Ni-treated *Arabidopsis thaliana* [409] and *Zea mays* [352].

The negative effect of Ni on mitotic activity may result from Ni-induced damage of nucleolar structure, chromosomal aberrations, abnormalities during mitosis and disturbance of mitotic spindle microtubules [237,353,370,406,410,411,412,413,414,415] (Figure 1 and Figure 3). The transmission electron microscopy study of root tip cells of *Solanum lycopersicum* illustrated that Ni induced a distortion in cell nucleus and nucleolus, the nuclear chromatin being condensed beside the nuclear envelope [237]. Disarrayed nuclear material and impaired nucleoli were found in Ni-treated *Citrullus lanatus* [146]. Nickel induced multiple mitotic disorders, including c-mitosis, indicating an inhibited spindle formation, as well as the appearance of micronuclei, anaphase bridges, chromosome stickiness, laggards, extrusion of nuclear material into the cytoplasm and other types of chromosomal aberrations [370,406,407,411,412,413]. The percentage of aberrations generally increased in a concentration- and time-dependent manner [406,415]. Notably, the MI was higher in the root meristematic cells of the seedlings of *Plantago lanceolata* from the serpentine population compared to the non-serpentine population, which is consistent with the higher Ni tolerance of the former [406]. Nickel is known to alter cytosine methylation patterns, causing either hypo- or hypermethylation of DNA. Hypomethylation may increase the susceptibility of chromosomes to breakage, whereas hypermethylation may cause chromosomal instability by inhibiting cell cycle–dependent checkpoint controls, which leads to deleterious effects on the cell [416,417]. The alkaline comet assay showed the integrity of the nuclei in leaf cells of *Noccaea caerulescens* grown in a Ni-enriched medium, whereas in the Ni-exposed non-tolerant species *Arabidopsis thaliana*, the nuclei were severely damaged. Remarkably, the DNA of *Noccaea caerulescens* grown in situ was considerably hyper-methylated compared to *Arabidopsis thaliana* exposed to Ni, whereas the *MET1*, *DRM2*, and *HDA8* genes involved in epigenetic DNA and histone modification were upregulated. Such epigenetic modifications may constitute a defense strategy that prevents genome instability and direct damage to the DNA structure caused by Ni [418].

In order to test metal cytotoxicity and genotoxicity for plants, *Allium sativum* and *Allium cepa* are often used because they have a small number of relatively large chromosomes that can be easily observed under microscope [407,411,412,413]. Nickel was shown to cause severe mitotic disturbances. The lowest metal concentrations that caused the mitotic disorders as estimated by Allium test can be ranged in the following order: Mn^2+^, Mg^2+^ (10^−2^ M) < Al^3+^, Cr^3+^ (10^−4^–10^−3^ M) < Cu^2+^, Co^2+^, Zn^2+^, Pb^2+^, Ni^2+^ (10^−5^ M) < Cd^2+^ and Hg^2+^ (10^−7^–10^−5^ M) [411]. A comparative analysis of the influence of Cd, Pb, Ni, Al, Cu and Zn salts at the concentrations of 10^−6^–10^−3^ M on cytogenetic parameters of *Allium cepa* root apical meristem cells revealed that NiSO_4_ had the strongest antimitotic activity, increasing the percentage of chromosome lagging, multipolar anaphases, C-mitoses by 69.6 times, compared to the control [412]. The ability of other metal salts to induce such abnormalities decreased in the range: CdCl_2_ > Al(NO_3_)_3_ > Pb(CH_3_COO)_2_ > CuSO_4_ > ZnSO_4_. Though, depending on whether EC_50_, sublethal or lethal concentrations are used, this series may greatly differ [413]. The comet assay revealed dose-dependent DNA damage and chromatin pulverization in the root tips of *Allium cepa* and *Vicia faba* exposed to Ni. In root cells, Ni induced several structural changes that were characteristic of apoptotic-like programmed cell death, such as condensed chromatin, cytoplasmic vacuolization, shrunken nuclei, and DNA fragmentation with the formation of apoptotic-like bodies [419]. Genotoxicity may arise from both direct reactions of metals with DNA or proteins associated with the DNA repair system, and metal-induced oxidative stress [217]. However, it is not fully clear yet, to what extent the direct interaction of Ni with DNA is manifested *in planta* and in what quantities Ni enters the nucleus at low concentrations in the medium.

The Ni-induced decrease in the length of fully-elongated cells indicates that it affects cell elongation [352] (Figure 3). The inhibition of cell elongation in Ni-treated plants may be determined by various mechanisms. Hydrogen peroxide formed as a result of Ni-induced oxidative stress may act as a substrate for peroxidases which play a role in cell wall stiffening, which could ultimately constrain cell elongation [153]. The presence of polysaccharides with different functional groups in plant cell wall provides binding sites for divalent and trivalent metals. Nickel ions bind preferentially to carboxyl groups of polygalacturonic acids and hydroxycinnamic acids, the proportion depending on the pH of the root apoplast and the plant species [420]. However, the Ni content in the apoplast is often lower than that in the protoplasts [325,362,421,422,423]. Metal binding to deesterified pectins of the cell wall can ultimately increase cell wall rigidity and result in cell rupture, inhibiting cell elongation [131]. In the roots of *Solanum lycopersicum*, Ni-induced cell wall thickening was suggested to act as a mechanism for metal detoxification [237]. Along with other cell wall modifications [424], it may reduce their plasticity. However, transcriptome data demonstrated that many genes involved in cell wall modifications were significantly repressed by high Ni level in *Arabidopsis thaliana* [131].

A detailed analysis of root growth inhibition in *Arabidopsis thaliana* revealed that Ni treatment reduced starch accumulation in columella cells, decreased the number of meristematic cells and the meristem length, and suppressed cell elongation without significantly disrupting the integrity of the stem cell niche. The analysis of auxin-responsive reporters revealed that Ni inhibited shootward distribution of auxin [131], which plays an important role in the regulation of root cell elongation and tropic growth [425]. It was demonstrated that the auxin transporter PIN2 was very sensitive to Ni, and its levels in roots were rapidly reduced in Ni-treated plants [131]. Nickel upregulated the expression of *PIN5* and *PIN10* genes, but downregulated the expression of *PIN4*, *PIN9* and *AUX1* genes in roots, suggesting that excess Ni interferes with root auxin transport and distribution [161]. Studying GUS-tagged *Arabidopsis* lines revealed that Ni-induced increase in the number of emerged lateral roots and concomitant decrease in the number of lateral root primordia were accompanied by elevated levels of auxin, cytokinin, and ethylene in or close to emerging lateral roots, whereas Ni-induced decrease in the length of primary roots was suggested to be associated with decreased auxin level and increased cytokinin and ethylene levels [397]. In Ni-treated *Solanum lycopersicum*, the concentrations of phytohormones in the roots, including auxin, cytokinin and gibberellic acid, decreased, thereby slowing down plant growth [161]. In any case, these data point to a Ni-induced disturbance of hormonal balance, which modulates root architecture (Figure 3). However, the molecular mechanisms behind these processes need to be further elucidated.

Damage to the cytoskeleton, which is involved in such fundamental processes as cellular division and elongation, polymer cross-linking and membrane anchorage have been observed in various plant species exposed to Ni (Figure 1 and Figure 3). At 5–20 μM Ni, the microfilaments of meristematic and elongating cells were affected in *Arabidopsis thaliana* [409]. Ni-induced ROS accumulation in roots also disrupted the integrity and orientation of cortical microtubules in the cells of the elongation zone. This could, in part, underlie root growth inhibition and compromised anisotropy of Ni-exposed roots [131]. Treatment of *Allium cepa* roots with 100 μM NiSO_4_ induced the appearance of thick microtubular bundles or aggregates in the perinuclear region of interphase cells and the disruption of the mitotic spindle [414]. Metal-induced disturbance of mineral nutrition and water regime can also have a negative effect on cell division and elongation (Figure 3).

Under natural growth conditions, plants are often exposed to the combined effects of metals, the study of which is a promising area of research. Under combined treatment of *Hibiscus sabdariffa* with Ni and Co (25 + 20 mg kg^−1^ soil), a more pronounced stimulation of growth was observed compared to separate treatments [199]. Mitigation of the toxic effects of Ni was demonstrated under the combined treatment of *Triticum aestivum* [238], *Oryza sativa* [426] and *Cucurbita pepo* [147] with Ni and Ca, as well as combined treatment of *Brassica juncea* [115], *Gossypium hirsutum* [135], *Oryza sativa* [111] and *Zea mays* [175,189] with Ni and Si. On the contrary, the combination of Ni and Cd in different doses proved to be more hazardous for the growth and productivity of *Vigna radiata* than the presence of a single metal in soil [241]. A more detailed study of the effects of combined treatments with different metals will make it possible to determine whether they act independently of each other or whether there is a synergistic or antagonistic effect in the action of different ions.

A decrease in the toxic effects of Ni was observed after addition of biochar [82,287], seed priming with citrulline [97] and titanium dioxide nanoparticles [176], exogenous plant treatment with ZnO nanoparticles [146], CeO_2_ nanoparticles [89], melatonin [162,163,427], 4-hydroxymelatonin [166], acetylcholine [100], metal chelating biopolymer chitosan (as a source of N) [143,152], ascorbic acid [428], citric acid [155], γ-aminobutyric acid [286], salicylic acid [70,108,109,116,157,158,294], jasmonic acid [106,110,157,158], putrescine [141], spermidine [113], proline [139,361], glycinebetaine [112,142,361], hydrogen peroxide [66], NaHS (a H_2_S donor) [94,148], and sodium nitroprusside (an NO donor) [78,108,109,113,114,130], as well as 24-epibrassinolide [69,74,150,151,164,296], 28-homobrassinolide [91], gibberellic acid [117], kinetin [356,429], ethephon (as a source of ethylene) [67,88] and other plant growth regulators [430]. However, the mechanisms underlying the alleviating effects of plant growth regulators as well as Ni effects on plant hormonal status remain insufficiently studied [431]. Inoculation of *Eruca sativa* with *Pseudomonas putida* [308], *Ocimum basilicum* with *Pseudomonas fluorescens* and *Pseudomonas putida* [156], *Vinca rosea* with *Bacillus megaterium* [172], *Solanum lycopersicum* with endophytic bacteria *Micrococcus luteus* and *Enterobacter cloacae* [72], as well as *Pongamia pinnata* with symbiotic nitrogen-fixing rhizobia *Rhizobium pisi* PZHK2 and *Ochrobacterium pseudogrignonense* PZHK4 [432] also resulted in alleviation of Ni phytotoxicity. In many cases, such effect was determined by a decrease in Ni accumulation in plants.

Metals can induce various changes in plant anatomy and morphology [424] (Figure 3), which influence different physiological processes, such as photosynthesis and transport of different substances (Figure 1 and Figure 2). In *Glycine max*, a decrease in the thickness of abaxial and adaxial leaf epidermis, as well as the diameter of root xylem and phloem vessels, started at 0.1 μM Ni. Changes in the structure of root vessels may lead to decreased turgor in shoot tissues, cause stomatal closure on the adaxial and abaxial leaf surface. The decrease in leaf epidermal thickness directly affects plant tolerance to mechanical, chemical and biological damage [92]. Treatment of *Triticum aestivum* with 1 mM NiSO_4_ led to a decrease in the thickness of leaf mesophyll, the size of the vascular bundles, the diameter of the xylem vessels in the central and lateral vascular bundles, and in the width of leaf epidermal cells [433]. When *Brassica oleracea* was grown on agar medium in the presence of 10 or 20 g m^−3^ NiSO_4_, a decrease in the volume of intercellular spaces in leaf mesophyll as well as the volume of spongy and palisade mesophyll cells was observed compared to control plants [280]. A reduction in the thickness of spongy parenchyma, adaxial and abaxial leaf epidermis, root rhizodermis, endodermis, and cortex, as well as the diameter of root vascular cylinder and metaxylem vessels was observed in Ni-stressed *Eucalyptus urophylla* [150], *Glycine max* [296] and *Solanum lycopersicum* [164].

Thus, various mechanisms underlie the Ni-induced growth inhibition: possible direct binding to DNA, metal-induced chromosome aberrations, prolongation of the mitotic cycle, disruption of microtubule formation, etc. (Figure 1, Figure 3 and Figure 4). Inhibition of cell division and elongation has been described for a wide variety of substances [398]. In further studies, it is important to establish whether there are species-specific differences in the sensitivity of these processes to the action of Ni and other metals.

## 10. Conclusions and Outlook

Nickel is an ultra-micronutrient essential for plant growth and development. However, at supra-optimal concentrations, Ni exerts multiple toxic effects on various physiological processes. It negatively impacts the activity of various enzymes, the composition and properties of membranes, mineral nutrition and water balance in plants, as well as respiration and photosynthesis, leading to disruption of plant growth and morphogenesis, reduced yield and productivity (Figure 1, Figure 2, Figure 3 and Figure 4). Despite significant progress in the understanding of the mechanisms of Ni toxic effects achieved in recent decades, the mechanisms of its influence on plant hormonal status and respiration, the combined effects of Ni and other metals, being particularly important in the case of polymetallic pollution of the environment, as well as the interspecific and intraspecific manifestations of Ni toxic effects, including ones at various stages of plant development, being directly related to plant tolerance and the efficiency of metal detoxification, remain insufficiently studied. Given the dual role of Ni as an ultra-micronutrient and a widespread potentially toxic element, as well as the large number of Ni hyperaccumulator species, a comparative analysis of Ni toxicity in plants with different accumulation strategies is a promising direction for further research. Studying the mechanisms of Ni toxic effects is of fundamental importance in plant physiology, toxicology, ionomics, genetics and ecology. It is also crucial for the development of new technologies in agriculture as well as technologies aimed at remediation and revegetation of metal-contaminated soils (phytoremediation) and metal extraction from the shoots and its subsequent use for obtaining metal-containing chemicals (phytomining).

## Figures and Tables

**Table 1:** The effects of Ni on enzyme activity (for antioxidant enzymes, see Section 3).

Enzyme	Process	Ni Content/ Concentration	Enzyme Activity	Plant Species	Refs.
Ribulose-1,5-bisphosphate carboxylase/oxygenase EC 4.1.1.39	Carbon fixation	200 mg kg^−1^ Ni in soil	**⇓**	*Brassica juncea*	[66,67]
		0.5, 1 mM	**⇓**	*Cajanus cajan*	[68]
Glyceraldehyde 3-phosphate dehydrogenase EC 1.2.1.12	Conversion of glyceraldehyde-3- phosphate to 1,3- bisphosphoglycerate	0.5, 1 mM	**⇓**	*Cajanus cajan*	[68]
3-Phosphoglycerate kinase EC 2.7.2.3	Transfer of a phosphate group from 1,3-bisphospho- glycerate to ADP	0.5, 1 mM	**⇓**	*Cajanus cajan*	[68]
Aldolase EC 4.1.2.13	Conversion of fructose- 1,6-bisphosphate to glyceraldehyde 3- phosphate and dihydroxyacetone phosphate	0.5, 1 mM	**⇓**	*Cajanus cajan*	[68]
Fructose 1,6-bisphosphatase EC 3.1.3.11	Conversion of fructose-1,6-bisphosphate into fructose-6-phosphate	0.5, 1 mM	**⇓**	*Cajanus cajan*	[68]
NAD-dependent and NADP-dependent glyceraldehyde-3-phosphate dehydrogenases EC 1.2.1.12, EC 1.2.1.9	Key enzymes in central metabolism, utilizing different coenzymes to perform distinct metabolic functions	0.5, 1 mM	**⇓**	*Cajanus cajan*	[68]
Carbonic anhydrase EC 4.2.1.1	Ion exchange, acid–base balance, carboxylation/ decarboxylation reactions	0.15 mM	**⇓**	*Brassica juncea*	[69]
		100, 150 mg kg^−1^ Ni in soil	**⇓**	*Catharanthus roseus*	[70]
		40–100 mg kg^−1^ Ni in soil	**⇓**	*Mentha arvensis*	[71]
		0.05 mM	**⇓**	*Solanum* *lycopersicum*	[72]
		50, 100, 150 mg kg^−1^ Ni in soil	**⇓**	*Vigna radiata*	[73,74]
Plasma membrane H^+^-ATPase EC 3.6.3.14	Creating vital electrochemical gradients	0.01, 0.1 mM	**⇓**	*Cucumis sativus*	[63]
		0.01 mM	0		[75]
		0.5 mM	**⇑**	*Oryza sativa*	[65]
Vacuolar H^+^-ATPase EC 3.6.3.14	ATP-dependent proton transport, ATP hydrolysis	0.01, 0.1 mM	**⇓**	*Cucumis sativus*	[76]
Vacuolar H^+^-pyrophosphatase EC 3.6.1.1	Transport of protons into the vacuole, breaking down pyrophosphate	0.01 mM	**⇑**	*Cucumis sativus*	[76]
Ca^2+^-ATPase EC 7.2.2.10	Active transport of calcium ions	100 ppm	**⇓**	*Vigna unguiculata*	[77]
		0.1 mM	**⇑**	*Zea mays*	[78]
Mg^2+^-ATPase EC 3.6.3.2	Active transport of magnesium ions	0.1 mM	**⇑**	*Zea mays*	[78]
Ferric-chelate reductase EC 1.16.1.7	Reduction of ferric iron to ferrous iron	0.025–0.1 mM	**⇓**	*Amaranthus pani* *culatus*	[79]
		0.02 mM	**⇓**	*Cucumis sativus*	[80]
		0.1, 0.3 mM	0	*Odontarrhena inflata*	[81]
Cu(II) reductase EC 1.7.2.1.	Reduction of copper from its oxidized to the cuprous state	0.1 mM	**⇑**	*Odontarrhena inflata*	[81]
Polyphenol oxidase EC 1.10.3.1, 1.10.3.2, or 1.14.18.1	Oxidation of phenols to quinones	400 mg kg^−1^ Ni in soil	**⇑**	*Anethum graveolens*	[82]
		0.005, 0.01, 0.02, 0.04 mM	**⇓**	*Hydrilla verticillata*	[83]
		330 mg kg^−1^ Ni in soil	**⇑**	*Lavandula* *angustifolia*	[84]
Phenylalanine ammonia- lyase EC 4.3.1.24	Conversion of L-phenylalanine into trans-cinnamic acid and ammonia	0.05–0.8 mM	**⇑**	*Luffa cylindric* *a*	[85]
Polyamine oxidase EC 1.5.3.3.	FAD amino-oxidase, producing H_2_O_2_	0.05, 0.1, 0.15 mM	**⇓**	*Amaranthus* *paniculatus*	[86]
		0.5, 1, 2, 3, 4 mM	**⇓**	*Hydrocharis dubia*	[87]
1-aminocyclopropane-1- carboxylic acid synthase EC 4.4.1.14	Biosynthesis of a precursor for ethylene	200 mg kg^−1^ Ni in soil	**⇑**	*Brassica juncea*	[67]
Pyrroline-5-carboxylate synthetase EC 2.7.2.11/1.2.2.41.	Biosynthesis of proline and ornithine from glutamate	200 mg kg^−1^ Ni in soil	**⇑**	*Brassica juncea*	[88]
γ-glutamyl kinase EC 2.7.2.11	The first enzyme of the proline biosynthetic pathway	200 mg kg^−1^ Ni in soil	**⇑**	*Brassica juncea*	[88]
Pyrroline-5-carboxylate reductase EC 1.5.1.2	Converts pyrroline- 5-carboxylate to proline	100 mg kg^−1^ Ni in soil	**⇑**	*Cicer arietinum*	[89]
Proline oxidase EC 1.5.99.8	Proline catabolism	200 mg kg^−1^ Ni in soil	**⇓**	*Brassica juncea*	[88]
Proline dehydrogenase EC 1.5.5.2	Proline catabolism	100 mg kg^−1^ Ni in soil	**⇑**	*Cicer arietinum*	[89]
Nitrate reductase EC 1.7.1.-	NO_3_^−^ reduction to nitrite (NO_2_^−^)	1 mM	**⇓**	*Be* *ta vulgaris*	[90]
		0.05, 0.1 mM	**⇓**	*Brassica juncea*	[91]
		200 mg kg^−1^ Ni in soil	**⇓**		[66,67]
		100, 150 mg kg^−1^ Ni in soil	**⇓**	*Catharanthus roseus*	[70]
		0.0001 mM 0.0005, 0.01, 0.02 mM	**⇑** **⇓**	*Glycine max*	[92]
		30, 60, 90 mg kg^−1^ Ni in soil	**⇓**	*Lens culinaris*	[93]
		0.05, 0.1, 0.2 mM	**⇓**	*Oryza sativa*	[94,95]
		0.2, 0.4 mM	**⇓**		[96]
		80, 100 mg kg^−1^ Ni in soil	**⇓**		[97]
		0.0006 mM	**⇑**	*Solanum* *lycopersicum*	[98]
		0.05 mM	**⇓**		[72]
		0.1 mM	**⇓**	*Triticum aestivum*	[99]
		0.5 mM	**⇓**		[100]
		0.05, 0.1 mM	**⇓**	*Verbascum olympicum*	[101]
		50, 100, 150 mg kg^−1^ Ni in soil	**⇓**	*Vigna radiata*	[73,74]
		0.5–3 mg kg^−1^ Ni in soil	**⇑**	*Vigna unguiculata*	[102]
		0.04 mM	**⇓**	*Zea mays*	[103]
Nitrite reductase EC 1.7.-.-	NO_2_^−^ reduction to NH_4_^+^	0.05, 0.1, 0.2 mM	**⇓**	*Oryza sativa*	[94,95]
		0.0006 mM	**⇑**	*Solanum* *lycopersicum*	[98]
		0.1 mM	**⇓**	*Triticum aestivum*	[99]
		0.04 mM	**⇑**	*Zea mays*	[103]
Glutamine synthetase EC 6.3.1.2	ATP-dependent amination of glutamate producing glutamine	1 mM	**⇓**	*Beta vulgaris*	[90]
		0.2 mM	**⇓**	*Oryza sativa*	[94,95]
		0.2, 0.4 mM	**⇓**		[96]
		0.0006 mM	**⇑**	*Solanum* *lycopersicum*	[98]
		0.05, 0.1 mM	**⇓**	*Triticum aestivum*	[99]
		0.05, 0.1 mM	**⇓**	*Verbascum olympicum*	[101]
Glutamate synthase EC 1.4.1.14	Glutamine convertion to glutamate	0.1, 0.2 mM	**⇓**	*Oryza sativa*	[94,95]
		0.0006 mM	**⇑**	*Solanum* *lycopersicum*	[98]
		0.1 mM	**⇓**	*Triticum aestivum*	[99]
Aminating glutamate dehydrogenase EC 1.4.1.-	Amination of 2-oxoglutarate to produce glutamate	0.4 mM	**⇑**	*Oryza sativa*	[96]
		0.05, 0.1 mM	**⇓**	*Triticum aestivum*	[99]
Deaminating glutamate dehydrogenase EC 1.4.1.-	Glutamate deamination to 2-oxoglutarate and ammonia	0.4 mM	**⇓**	*Oryza sativa*	[96]
		0.05, 0.1 mM	**⇓**	*Triticum aestivum*	[99]
Glutamate oxaloacetate transaminase EC 2.6.1.1	Conversion between aspartate/glutamate and oxaloacetate/ α-ketoglutarate	0.05, 0.1, 0.2 mM	**⇓**	*Oryza sativa*	[94,95]
Alanine aminotransferase EC 2.6.1.2	Reversible conversion of L-alanine and α-ketoglutarate into L-glutamate and pyruvate	0.2 mM	**⇓**	*Glycine max*	[104]
		0.1, 0.2 mM	**⇓**	*Oryza sativa*	[94,95]
		0.4 mM	**⇑**		[96]
		0.05, 0.1 mM	**⇑**	*Triticum aestivum*	[99]
Aspartate aminotransferase EC 2.6.1.1	Biosynthesis of aspartate from glutamate and oxaloacetate	0.2 mM	**⇓**	*Glycine max*	[104]
		0.2, 0.4 mM	**⇑**	*Oryza sativa*	[96]
ATP-sulfurylase EC 2.7.7.4	Activation of inorganic sulfate	200 mg kg^−1^ Ni in soil	**⇑**	*Brassica juncea*	[66]
IAA oxidase EC 1.2.3.7	IAA oxidation	<0.05 mM >0.05 mM	**⇑** **⇓**	*Oryza sativa*	[105]
NADPH oxidase EC 1.6.3.1	Key enzyme for the generation of ROS	2 mM	**⇑**	*Glycine max*	[106]
		1 mM	**⇑**	*Triticum durum*	[107]
Lipoxygenase EC 1.13.11.-	Oxygenation of polyunsaturated fatty acids (initiation of lipid peroxidation)	0.5 mM	**⇑**	*Brassica napus*	[108]
		0.5 mM	**⇑**	*Eleusine coracana*	[109]
		4 mM	**⇑**	*Glycine max*	[110]
		0.25, 0.5 mM	**⇑**	*Oryza sativa*	[111]
		80, 100 mg kg^−1^ Ni in soil	**⇑**		[97]
		0.023 mM	**⇑**	*Pennisetum* *typhoideum*	[112]
		0.05 mM	**⇑**	*Solanum* *lycopersicum*	[72]
		1 mM	**⇑**		[113]
		100 mg kg^−1^ Ni in soil	**⇑**	*Solanum melongena*	[114]
Ascorbate oxidase EC 1.10.3.3.	Oxidation of L-ascorbic acid to dehydroascorbic acid	<0.05 mM >0.05 mM	**⇑** **⇓**	*Oryza sativa*	[105]
Glyoxalase I EC 4.4.1.5	Conversion of methylglyoxal and reduced glutathione to S-D-lactoylglutathione	0.05, 0.1, 0.15 mM	**⇑**	*Brassica juncea*	[115]
		0.1, 0.15 mM	**⇑**		[116]
		200 mg kg^−1^ Ni in soil	**⇑**		[88]
		0.1 mM	**⇑**	*Glycine max*	[117]
		4 mM	**⇓**		[110]
		0.25, 0.5 mM	**⇑**	*Oryza sativa*	[111]
		1 mM	**⇑**	*Solanum* *lycopersicum*	[113]
		100 mg kg^−1^ Ni in soil	**⇑**	*Solanum melongena*	[114]
Glyoxalase II EC 3.1.2.6	Transformation of S-D-lactoylglutathione into D-lactic acid and glutathione	0.05, 0.1, 0.15 mM	**⇓**	*Brassica juncea*	[115]
		0.1, 0.15 mM	**⇓**		[116]
		200 mg kg^−1^ Ni in soil	**⇑**		[88]
		0.1 mM	**⇑**	*Glycine max*	[117]
		4 mM	**⇓**		[110]
		0.25, 0.5 mM	**⇑**	*Oryza sativa*	[111]
		1 mM	**⇑**	*Solanum* *lycopersicum*	[113]
Urease EC 3.5.1.5	The conversion of urea into carbon dioxide and ammonia	0.05, 0.1, 0.2 mM	**⇑**	*Cucumis sativus*	[118]
		0.0001, 0.0005, 0.01, 0.02 mM	**⇑**	*Glycine max*	[92]
		0.00085 mM	**⇑**		[119]
		0.25–9 mg kg^−1^ Ni in soil	**⇑**		[120]
		1–3 mg kg^−1^ Ni in soil	**⇑**	*Vigna unguiculata*	[102]

**⇓**—Activity of the enzyme decreased; **⇑**—activity of the enzyme increased; 0—activity of the enzyme did not change. The plants were grown in hydroponics (Ni concentration is given in mM or mg L^−1^) or in soil (mg kg^−1^ Ni in soil). Hyperaccumulator species are marked grey. For Ni effects on the activity of antioxidant enzymes, see Section 3.

**Table 2:** The effects of Ni on the contents of malondialdehyde, reactive oxygen species and methylglyoxal, as well as electrolyte leakage.

Plant Species	Ni Content/ Concentration	Plant Organs	MDA/ TBARS	O_2_^•−^	OH^•^	H_2_O_2_	EL	MG	Refs.
*Amaranthus* *paniculatus*	0.05, 0.1 mM	leaves	**⇑**						[79]
	0.1, 0.15 mM	roots leaves	**⇑** **⇑**						[86]
*Anethum graveolens*	400 mg kg^−1^ Ni in soil	leaves	**⇑**			**⇑**			[82]
*Atropa belladonna*	0.2 mM	leaves	**⇑**						[139]
*Brassica juncea*	0.05, 0.1, 0.15 mM	leaves	**⇑**			**⇑**	**⇑**	**⇑**	[115]
	0.05, 0.1, 0.15 mM	plants	**⇑** ^(1)^			**⇑**	**⇑**	**⇑**	[116]
	0.15 mM	leaves	**⇑**				**⇑**		[69]
	0.1, 0.2, 0.4 mM	seedlings	**⇓**	**⇑**		**⇓** ^(3)^			[140]
	200 mg kg^−1^ Ni in soil	leaves	**⇑**			**⇑**	**⇑**	**⇑**	[66,67,88]
*Brassica napus*	0.5 mM	leaves	**⇑**			**⇑**			[108]
	0.25, 0.5 mM	leaves	**⇑**						[141]
	50, 100 mg kg^−1^ Ni in soil	plants	**⇑**			**⇑**			[142]
*Calendula tripterocarpa*	100, 150 mg kg^−1^ Ni in soil	roots leaves	**⇑** **⇑**						[143]
*Capsicum annuum*	10, 20, 30, 50, 75, 100 mg L^−1^	roots leaves	**⇑** **⇑**	**⇑** **⇑**		**⇑** **⇑**			[144]
*Carthamus tinctorius*	0.5, 0.75, 1 mM	leaves				**⇑**	**⇑**		[145]
*Catharanthus roseus*	50, 100, 150 mg kg^−1^ Ni in soil	leaves					**⇑**		[70]
*Carthamus* *oxyacantha*	0.5, 0.75, 1 mM	leaves				**⇑** ^(5)^	**⇑**		[145]
*Chenopodium quinoa*	0.1, 0.2, 0.3, 0.4 mM	leaves	**⇑**						[137]
*Cicer arietinum*	100 mg kg^−1^ Ni in soil	leaves	**⇑**			**⇑**			[89]
*Citrullus lanatus*	70 mg kg^−1^ Ni in soil	leaves	**⇑**	**⇑**		**⇑**	**⇑**		[146]
*Cucumis sativus*	0.01 mM	leaves	**⇑**	**⇑**		**⇑**	**⇑**		[132]
*Cucurbita pepo*	0.273 mM	roots leaves	**⇑** **⇑**			**⇑** **⇑**	**⇑** **⇑**		[147,148]
*Eleusine coracana*	0.5 mM	roots shoots		**⇑** **⇑**		**⇑** **⇑**			[109]
	0.25, 0.5, 1 mM	roots shoots	**⇑** ^(6)^ **⇑** ^(6)^			**⇑** ^(6)^ **⇑** ^(6)^	**⇑** **⇑**		[149]
*Eucalyptus urophylla*	0.6 mM	plants	**⇑**	**⇑**		**⇑**	**⇑**		[150]
*Glycine max*	0.1 mM	leaves	**⇑**			**⇑**	**⇑**	**⇑**	[117]
	0.2 mM	plants	**⇑**	**⇑**		**⇑**	**⇑**		[151]
	2 mM	seedlings	**⇑**			**⇑**			[106]
	4 mM	leaves	**⇑**			**⇑**	**⇑**	**⇑**	[110]
	200 mg kg^−1^ Ni in soil	leaves	**⇑**						[152]
*Gossypium hirsutum*	0.05, 0.1 mM	roots leaves	**⇑** **⇑**			**⇑** **⇑**	**⇑** **⇑**		[135]
*Hydrilla verticillata*	0.005, 0.01, 0.02, 0.04 mM	leaves stems	**⇑** ^(4)^ **⇑**						[83]
*Hydrocharis dubia*	1, 2, 3, 4 mM	leaves	**⇑**	**⇑**					[87]
*Ipomoea batatas*	15, 30, 60 mg L^−1^	leaves	**⇑**			**⇑**			[153]
*Lavandula* *angustifolia*	220, 330 mg kg^−1^ Ni in soil	leaves	**⇑**			**⇑**			[84]
*Medicago* *sativa*	50, 150, 250, 500 mg kg^−1^ Ni in soil	roots leaves	**⇑** **⇑**						[154]
*Mentha piperita*	0.1, 0.25, 0.5 mM	roots leaves	**⇑** **⇑**			**⇑** **⇑**	**⇑** **⇑**		[155]
*Ocimum basilicum*	75, 100, 150 mg kg^−1^ Ni in soil	leaves					**⇑**		[156]
*Odontarrhena inflata*	0.1, 0.2, 0.4 mM	roots shoots				**⇑** **⇑**			[157,158]
*Oryza sativa*	0.05, 0.2 mM	roots shoots	**⇑** **⇑**			**⇑** **⇑**	**⇑** **⇑**		[130]
	0.1, 0.2 mM	roots shoots	**⇑** ^(2)^ **⇑**			**⇑** **⇑**			[129]
	0.2, 0.4 mM	roots shoots	**⇑** **⇑**	**⇑** **⇑**		**⇑** **⇑**			[136]
	0.25, 0.5 mM	leaves	**⇑**			**⇑**		**⇑**	[111]
	0.5 mM	roots					**⇑**		[64]
	80, 100 mg kg^−1^ Ni in soil	leaves	**⇑**	**⇑**	**⇑**	**⇑**	**⇑**	**⇑**	[97]
*Pennisetum* *typhoideum*	0.023 mM	radicles	**⇑**	**⇑**	**⇑**	**⇑**			[112]
*Pisum sativum*	0.1 mM	leaves	**⇑**				**⇑**		[159]
*Sesuvium* *portulacastrum*	0.05, 0.1 mM	shoots	**⇑**						[160]
*Solanum lycopersicum*	0.03, 0.05 mM	roots leaves	**⇑** 0			**⇑** **⇑**			[161]
	0.05 mM	roots leaves	**⇑** **⇑**	**⇑** **⇑**		**⇑** **⇑**	**⇑** **⇑**		[162]
	0.05 mM	roots leaves	**⇑** **⇑**	**⇑** **⇑**		**⇑** **⇑**	**⇑** **⇑**		[163]
	0.05 mM	plants	**⇑**	**⇑**	**⇑**	**⇑**			[72]
	0.4 mM	plants	**⇑**	**⇑**		**⇑**	**⇑**		[164]
	1 mM	plants	**⇑**			**⇑**		**⇑**	[113]
*Solanum melongena*	0.2, 0.3, 0.4 mM	leaves	**⇓**						[165]
	100 mg kg^−1^ Ni in soil	leaves	**⇑**			**⇑**	**⇑**	**⇑**	[114,166]
*Solanum nigrum*	0.1 mM	roots shoots	0 **⇑**	**⇑** 0					[167]
*Triticum aestivum*	0.05, 0.1 mM	roots shoots	0 **⇑**			**⇑** **⇑**	**⇑** **⇑**		[168]
	0.1 mM	leaves		**⇑**		**⇑**			[169]
	0.1, 0.2 mM	roots leaves				**⇑** **⇑**			[136]
	0.5 mM	plants	**⇑**			**⇑**	**⇑**		[100]
*Triticum durum*	1 mM	roots	**⇑**	**⇑**		**⇑**			[107]
*Vicia sativa*	0.005–1 mM	leaves				**⇑**			[170]
*Vigna radiata*	0.2 mM	leaves	**⇑**			**⇑**			[171]
	150 mg kg^−1^ Ni in soil	leaves					**⇑**		[73]
*Vinca rosea*	50, 100, 200 mg kg^−1^ Ni in soil	leaves	**⇑**			**⇑**			[172]
*Zea mays*	0.084, 0.168 mM	leaves	**⇑**						[173]
	0.25 mM	shoots	**⇑**						[174]
	0.1 mM	roots leaves	**⇑** **⇑**			**⇑** **⇑**			[175]
	0.1 mM	roots leaves	**⇑** **⇑**	**⇑** **⇑**		**⇑** **⇑**			[176]
	0.1 mM	leaves	**⇑**			**⇑**			[140]

**⇓**—Decrease; **⇑**—increase; 0—no change. If the cells are empty, no measurements were taken. Malondialdehyde (MDA), superoxide radicals (O_2_^•−^), hydroxyl radicals (OH^•^), hydrogen peroxide (H_2_O_2_), methylglyoxal (MG), electrolyte leakage (EL). ^(1)^ Only at 0.1, 0.15 mM Ni; ^(2)^ only at 0.2 mM Ni; ^(3)^ only at 0.2 and 0.4 mM Ni; ^(4)^ only at 0.005 and 0.01 mM Ni; ^(5)^ only at 0.5 mM Ni; ^(6)^ only at 1 mM. The plants were grown in hydroponics (Ni concentration given in mM or mg L^−1^) or in soil (mg kg^−1^ Ni in soil). Hyperaccumulator species are marked grey.

**Table 3:** The effects of Ni the activity of antioxidant enzymes.

Enzyme	Process	Ni Content/Concentration	Enzyme Activity	Plant Species	Refs.
Glutathione reductase EC 1.8.1.7	NADPH-dependent reduction of oxidized glutathione (GSSG) to reduced form (GSH)	0.01–1 mM	**⇑**	*Alyssum maritimum* (*Lobularia maritima*) *Alyssum argenteum*	[179]
		0.02 mM	**⇓**	*Brassica campestris*	[181]
		0.05, 0.1, 0.15 mM	**⇑**	*Brassica juncea*	[115,116]
		0.15 mM	**⇑**		[69]
		200 mg kg^−1^ Ni in soil	**⇑**		[66,67,88]
		0.5, 1, 1.5 mM	**⇑**	*Cajanus cajan*	[182]
		20–100 mg L^−1^	**⇑**	*Capsicum annuum*	[144]
		0.5, 1 mM	**⇑**	*Eleusine coracana*	[149]
		4 mM	**⇓**	*Glycine max*	[110]
		0.1 mM	**⇑**	*Lemna minor*	[183]
		0.2, 0.4 mM	**⇑**	*Oryza sativa*	[134]
		0.25, 0.5 mM	**⇑**		[111]
		0.1 mM	**⇑**	*Pisum sativum*	[159]
		0.518 mM	**⇑**	*Sesbania drummondii*	[184]
		0.05 mM	**⇓**	*Solanum lycopersicum*	[162,163]
		0.05 mM	**⇑**		[72]
		1 mM	**⇑**		[113]
		100 mg kg^−1^ Ni in soil	**⇑**	*Solanum melongena*	[114]
		0.5 mM	**⇑**	*Triticum aestivum*	[100]
		0.01 mM 0.2 mM	**⇑** **⇓**	*Vigna radiata*	[171]
		0.168 mM	**⇑**	*Zea mays*	[173]
		0.25 mM	**⇑**		[174]
Glutathione S-transferase EC 2.5.1.18	Conjugation of electrophilic substrates to reduced glutathione	0.05, 0.1, 0.15 mM	**⇑**	*Brassica juncea*	[115,116]
		20–100 mg L^−1^	**⇑**	*Capsicum annuum*	[144]
		4 mM	**⇓**	*Glycine max*	[110]
		50, 150, 250, 500 mg kg^−1^ Ni in soil	**⇑**	*Medicago sativa*	[154]
		0.25, 0.5 mM	**⇓**	*Oryza sativa*	[111]
		80, 100 mg kg^−1^ Ni in soil	**⇑**		[97]
		100 mg kg^−1^ Ni in soil	**⇑**	*Solanum melongena*	[114]
		0.2 mM	**⇑**	*Triticum aestivum*	[185]
		100, 150 mg kg^−1^ Ni in soil	**⇑**		[180]
		0.1 mM	**⇑**	*Zea mays*	[78]
Glutathione peroxidase EC 1.11.1.9	Reduction of hydrogen peroxide (H_2_O_2_) and organic peroxides to water and alcohols, respectively, using glutathione as a reducing agent	200 mg kg^−1^ Ni in soil	**⇑**	*Brassica juncea*	[88]
		4 mM	**⇓**	*Glycine max*	[110]
		220, 330 mg kg^−1^ Ni in soil	**⇑**	*Lavandula angustifolia*	[84]
		0.2 mM	**⇑**	*Lemna minor*	[183]
		0.25, 0.5 mM	**⇑**	*Oryza sativa*	[111]
		0.1 mM	**⇑**	*Triticum aestivum*	[169]
		50, 100, 150 mg kg^−1^ Ni in soil	**⇑**		[180]
		0.1 mM	**⇑**	*Zea mays*	[78]
Ascorbate peroxidase EC 1.11.1.11	Reduction of hydrogen peroxide (H_2_O_2_) using ascorbate as an electron donor	0.15, 0.3 mM	**⇑**	*Aurinia saxatilis*	[138]
		0.05, 0.1, 0.15 mM	**⇑**	*Brassica juncea*	[115,116]
		0.1, 0.2 mM	**⇓**		[140]
		0.2, 0.3, 0.4 mM	**⇑**		[186]
		200 mg kg^−1^ Ni in soil	**⇑**		[66,67,88]
		0.5 mM	**⇓**	*Brassica napus*	[108]
		0.5, 0.75, 1 mM	**⇑**	*Carthamus oxyacantha*	[145]
		0.5, 0.75, 1 mM	**⇑**	*Carthamus tinctorius*	[145]
		20–100 mg L^−1^	**⇑**	*Capsicum annuum*	[144]
		0.1, 0.2, 0.3, 0.4 mM	**⇑**	*Chenopodium quinoa*	[137]
		100 mg kg^−1^ Ni in soil	**⇑**	*Cicer arietinum*	[89]
		70 mg kg^−1^ Ni in soil	**⇑**	*Citrullus lanatus*	[146]
		0.5 mM	**⇓**	*Eleusine coracana*	[109]
		0.6 mM	**⇑**	*Eucalyptus urophylla*	[150]
		0.1 mM	**⇑**	*Glycine max*	[117]
		0.2 mM	**⇑**		[151]
		2 mM	**⇑**		[106]
		4 mM	**⇓**		[110]
		200 mg kg^−1^ Ni in soil	**⇑**		[152]
		0.05, 0.1 mM	**⇑**	*Gossypium hirsutum*	[135]
		7.5–60 mg L^−1^	**⇑**	*Ipomoea batatas*	[153]
		330 mg kg^−1^ Ni in soil	**⇑**	*Lavandula angustifolia*	[84]
		0.1, 0.2 mM	**⇓**	*Lemna minor*	[183]
		0.1, 0.25, 0.5 mM	**⇑**	*Mentha piperita*	[155]
		0.35 mM	**⇓**	*Odontarrhena* *inflata*	[81]
		0.1, 0.2, 0.4 mM	**⇑**		[157,158]
		0.3 mM	**⇑**		[138]
		0.2, 0.4 mM	**⇑**	*Oryza sativa*	[134]
		0.25, 0.5 mM	**⇑**		[111]
		80, 100 mg kg^−1^ Ni in soil	**⇑**		[97]
		0.023 mM	**⇑**	*Pennisetum typhoideum*	[112]
		0.1 mM	**⇑**	*Pisum sativum*	[159]
		0.518 mM	**⇑**	*Sesbania drummondii*	[184]
		0.05 mM	**⇓**	*Solanum lycopersicum*	[162,163]
		0.05 mM	**⇑**		[72]
		0.4 mM	**⇑**		[164]
		1 mM	**⇑**		[113]
		100 mg kg^−1^ Ni in soil	**⇑**	*Solanum melongena*	[114]
		0.1 mM	**⇑**	*Triticum aestivum*	[169]
		0.5 mM	**⇑**		[100]
		0.05, 0.1 mM	**⇑**	*Verbascum olympicum*	[101]
		0.01, 0.1, 0.2 mM	**⇑**	*Vigna radiata*	[171]
		50, 100, 150 mg kg^−1^ Ni in soil	**⇑**	*Vinca rosea*	[172]
		1–3 mg kg^−1^ Ni in soil	**⇓**	*Vigna unguiculata*	[102]
		0.084, 0.168 mM	**⇑**	*Zea mays*	[173]
		0.1 mM	**⇑**		[176]
		0.25 mM	**⇑**		[174]
		0.25 mM	**⇑**		[187]
Monodehydroascorbate reductase EC 1.6.5.4	Reduction of the monodehydroascorbate radical to ascorbate	0.05, 0.1, 0.15 mM	**⇓**	*Brassica juncea*	[115,116]
		4 mM	**⇓**	*Glycine max*	[110]
		0.2, 0.4 mM	**⇑**	*Oryza sativa*	[134]
		0.25, 0.5 mM	**⇑**		[111]
		0.05 mM	**⇑**	*Solanum lycopersicum*	[162]
		1 mM	**⇑**		[113]
		0.25 mM	**⇑**	*Zea mays*	[187]
Dehydroascorbate reductase EC 1.8.5.1	Reduction of dehydroascorbate to ascorbate using reduced glutathione as the electron donor	0.02 mM	**⇓**	*Brassica campestris*	[181]
		0.05, 0.1, 0.15 mM	**⇓**	*Brassica juncea*	[115,116]
		4 mM	**⇓**	*Glycine max*	[110]
		0.2, 0.4 mM	**⇑**	*Oryza sativa*	[134]
		0.25, 0.5 mM	**⇑**		[111]
		0.05 mM	**⇑**	*Solanum lycopersicum*	[162]
		1 mM	**⇑**		[113]
		0.5 mM	**⇑**	*Triticum aestivum*	[100]
		0.1 mM	**⇑**	*Zea mays*	[78]
Superoxide dismutase EC 1.15.1.1.	Dismutation of superoxide radicals (O_2_^∙−^) into molecular oxygen (O_2_) and hydrogen peroxide (H_2_O_2_)	0.01 mM 0.1 mM	**⇓** **⇑**	*Alyssum maritimum* (*Lobularia maritima*)	[179]
		0.02 mM	**⇓**	*Brassica campestris*	[181]
		0.05, 0.1, 0.15 mM	**⇑**	*Brassica juncea*	[115,116]
		0.1, 0.2 mM	**⇑**		[140]
		0.15 mM	**⇑**		[69]
		0.2, 0.3, 0.4 mM	**⇑**		[186]
		200 mg kg^−1^ Ni in soil	**⇑**		[67,88]
		50, 100 mg kg^−1^ Ni in soil	**⇑**	*Brassica napus*	[142]
		7.5–60 mg L^−1^	**⇑**	*Ipomoea batatas*	[153]
		0.5, 1, 1.5 mM	**⇑**	*Cajanus cajan*	[182]
		20–100 mg L^−1^	**⇑**	*Capsicum annuum*	[144]
		0.5, 0.75, 1 mM	**⇑**	*Carthamus oxyacantha*	[145]
		0.5, 0.75, 1 mM	**⇑**	*Carthamus tinctorius*	[145]
		50, 100, 150 mg kg^−1^ Ni in soil	**⇑**	*Catharanthus roseus*	[70]
		0.1, 0.2, 0.3, 0.4 mM	**⇑**	*Chenopodium quinoa*	[137]
		100 mg kg^−1^ Ni in soil	**⇑**	*Cicer arietinum*	[89]
		70 mg kg^−1^ Ni in soil	**⇑**	*Citrullus lanatus*	[146]
		0.273 mM	**⇑**	*Cucurbita pepo*	[147]
		0.5 mM	**⇓**	*Eleusine coracana*	[109]
		0.5, 1 mM	**⇑**		[149]
		0.6 mM	**⇑**	*Eucalyptus urophylla*	[150]
		0.0005, 0.01, 0.02 mM	**⇑**	*Glycine max*	[92]
		0.1 mM	**⇑**		[117]
		0.2 mM	**⇑**		[151]
		2 mM	**⇑**		[106]
		200 mg kg^−1^ Ni in soil	**⇑**		[152]
		0.05, 0.1 mM	**⇑**	*Gossypium hirsutum*	[135]
		0.005, 0.01, 0.02, 0.04 mM	**⇑**	*Hydrilla verticillata*	[83]
		1, 2, 3, 4 mM	**⇓**	*Hydrocharis dubia*	[87]
		600 mg kg^−1^ Ni in soil	**⇑**	*Lactuca sativa*	[188]
		220, 330 mg kg^−1^ Ni in soil	**⇑**	*Lavandula angustifolia*	[84]
		0.1 mM	**⇑**	*Lemna minor*	[183]
		0.05–0.8 mM	**⇑**	*Luffa cylindrica*	[85]
		20–100 mg kg^−1^ Ni in soil	**⇑**	*Mentha arvensis*	[71]
		0.1, 0.25, 0.5 mM	**⇑**	*Mentha piperita*	[155]
		0.1, 0.2, 0.4 mM	**⇑**	*Odontarrhena inflata*	[157,158]
		0.05, 0.1, 0.2 mM	**⇓**	*Oryza sativa*	[129]
		0.05, 0.2 mM	**⇓**		[130]
		0.2, 0.4 mM	**⇑**		[134]
		0.25, 0.5 mM	**⇑**		[111]
		80, 100 mg kg^−1^ Ni in soil	**⇑**		[97]
		0.023 mM	**⇑**	*Pennisetum typhoideum*	[112]
		0.1 mM	**⇑**	*Pisum sativum*	[159]
		0.518 mM	**⇑**	*Sesbania drummondii*	[184]
		0.03, 0.05 mM	**⇑**	*Solanum lycopersicum*	[161]
		0.05 mM	**⇓**		[162,163]
		0.05 mM	**⇑**		[72]
		0.4 mM	**⇑**		[164]
		1 mM	**⇑**		[113]
		0.3, 0.4 mM	**⇑**	*Solanum melongena*	[165]
		100 mg kg^−1^ Ni in soil	**⇑**		[114,166]
		0.1 mM	**⇓**	*Triticum aestivum*	[169]
		0.2 mM	**⇓**		[185]
		0.5 mM	**⇑**		[100]
		0.05, 0.1 mM	**⇑**	*Verbascum olympicum*	[101]
		0.01, 0.1, 0.2 mM	**⇑**	*Vigna radiata*	[171]
		50, 100, 150 mg kg^−1^ Ni in soil	**⇑**		[73,74]
		2, 3 mg kg^−1^ Ni in soil	**⇑**	*Vigna unguiculata*	[102]
		50, 100, 150 mg kg^−1^ Ni in soil	**⇑**	*Vinca rosea*	[172]
		0.084, 0.168 mM	**⇑**	*Zea mays*	[173]
		0.1 mM	**⇑**		[175]
		0.1 mM	**⇑**		[78]
		0.1 mM	**⇑**		[176]
		0.1 mM	**⇑**		[189]
		0.25 mM	**⇑**		[174]
Catalase EC 1.11.1.6.	Conversion of hydrogen peroxide (H_2_O_2_) into water (H_2_O) and oxygen (O_2_)	0.3 mM	**⇑**	*Aurinia saxatilis*	[138]
		0.02 mM	**⇓**	*Brassica campestris*	[181]
		0.05, 0.1 mM	**⇑**	*Brassica juncea*	[91]
		0.05, 0.1, 0.15 mM	**⇓**		[115,116]
		0.1, 0.2 mM	**⇓**		[140]
		0.1, 0.2, 0.3, 0.4 mM	**⇓**		[186]
		0.5 mM	**⇓**	*Brassica napus*	[108]
		50, 100 mg kg^−1^ Ni in soil	**⇑**		[142]
		0.5 mM	**⇓**	*Brassica oleracea*	[190]
		0.5, 1, 1.5 mM	**⇓**	*Cajanus cajan*	[182]
		20–100 mg L^−1^	**⇑**	*Capsicum annuum*	[144]
		50, 100, 150 mg kg^−1^ Ni in soil	**⇑**	*Catharanthus roseus*	[70]
		0.1, 0.2, 0.3, 0.4 mM	**⇑**	*Chenopodium quinoa*	[137]
		100 mg kg^−1^ Ni in soil	**⇑**	*Cicer arietinum*	[89]
		70 mg kg^−1^ Ni in soil	**⇑**	*Citrullus lanatus*	[146]
		0.273 mM	**⇓**	*Cucurbita pepo*	[147]
		0.5 mM	**⇓**	*Eleusine coracana*	[109]
		0.5, 1 mM	**⇑**		[149]
		0.6 mM	**⇑**	*Eucalyptus urophylla*	[150]
		0.00005–0.02 mM	**⇑**	*Glycine max*	[92]
		0.1 mM	**⇑**		[117]
		0.2 mM	**⇑**		[151]
		2 mM	**⇑**		[106]
		4 mM	**⇓**		[110]
		200 mg kg^−1^ Ni in soil	**⇑**		[152]
		0.1 mM	**⇓**	*Gossypium hirsutum*	[135]
		0.005, 0.01 mM	**⇑**	*Hydrilla verticillata*	[83]
		0.5, 1, 2, 3, 4 mM	**⇓**	*Hydrocharis dubia*	[87]
		15–60 mg L^−1^	**⇑**	*Ipomoea batatas*	[153]
		600 mg kg^−1^ Ni in soil	**⇑**	*Lactuca sativa*	[188]
		0.1, 0.2 mM	**⇓**	*Lemna minor*	[183]
		0.05–0.8 mM	**⇑**	*Luffa cylindrica*	[85]
		20–100 mg kg^−1^ Ni in soil	**⇑**	*Mentha arvensis*	[71]
		0.1, 0.25, 0.5 mM	**⇑**	*Mentha piperita*	[155]
		<0.05 mM >0.05 mM	**⇑** **⇓**	*Oryza sativa*	[105]
		0.05, 0.2 mM	**⇑**		[130]
		0.1, 0.2 mM	**⇑**		[129]
		0.5 mM	**⇓**		[111]
		80, 100 mg kg^−1^ Ni in soil	**⇑**		[97]
		0.1, 0.2, 0.4 mM	**⇑**	*Odontarrhena inflata*	[157,158]
		0.3 mM	**⇑**		[138]
		0.35 mM	**⇓**		[81]
		0.023 mM	**⇑**	*Pennisetum typhoideum*	[112]
		0.1 mM	**⇑**	*Pisum sativum*	[159]
		0.1, 0.2, 0.5, 1 mM	**⇑**	*Raphanus sativus*	[191]
		0.03, 0.05 mM	0	*Solanum lycopersicum*	[161]
		0.05 mM	**⇓**		[162,163]
		0.05 mM	**⇑**		[72]
		0.4 mM	**⇑**		[164]
		0.05, 0.1, 0.2, 0.3, 0.4 mM	**⇓**	*Solanum melongena*	[165]
		100 mg kg^−1^ Ni in soil	**⇓**		[114]
		100 mg kg^−1^ Ni in soil	**⇑**		[166]
		0.1, 0.2, 0.3, 0.4, 0.5 mM	**⇓**	*Solanum tuberosum*	[192]
		0.1 mM	**⇓**	*Triticum aestivum*	[169]
		0.2 mM	**⇓**		[185]
		0.5 mM	**⇑**		[100]
		0.05, 0.1 mM	**⇑**	*Verbascum olympicum*	[101]
		0.001, 0.005, 0.01 mM	**⇑**	*Vicia sativa*	[170]
		0.2 mM	**⇓**	*Vigna radiata*	[171]
		50, 100, 150 mg kg^−1^ Ni in soil	**⇑**		[73,74]
		0.5–3 mg kg^−1^ Ni in soil	**⇑**	*Vigna unguiculata*	[102]
		50, 100, 150 mg kg^−1^ Ni in soil	**⇓**	*Vinca rosea*	[172]
		0.084, 0.168 mM	**⇑**	*Zea mays*	[173]
		0.1 mM	**⇓**		[175]
		0.1 mM	**⇑**		[78]
		0.1 mM	**⇑**		[176]
		0.1 mM	**⇑**		[189]
		0.25 mM	**⇑**		[187]
		0.25 mM	**⇑**		[174]
Peroxidase EC 1.11.1.7.	Reduction of hydrogen peroxide (H_2_O_2_) using several reductants	400 mg kg^−1^ Ni in soil	**⇓**	*Anethum graveolens*	[82]
		0.15, 0.3 mM	**⇑**	*Aurinia saxatilis*	[138]
		0.02 mM	**⇓**	*Brassica campestris*	[181]
		0.05, 0.1 mM	**⇑**	*Brassica juncea*	[91]
		0.15 mM	**⇑**		[69]
		0.1, 0.2, 0.3, 0.4 mM	**⇑**		[186]
		0.5 mM	**⇓**	*Brassica napus*	[108]
		50, 100 mg kg^−1^ Ni in soil	**⇑**		[142]
		0.5 mM	**⇓**	*Brassica oleracea*	[190]
		0.5, 1, 1.5 mM	**⇑**	*Cajanus cajan*	[182]
		10–100 mg L^−1^	**⇑**	*Capsicum annuum*	[144]
		0.5, 0.75, 1 mM	**⇑**	*Carthamus oxyacantha*	[145]
		0.5, 0.75, 1 mM	**⇑**	*Carthamus tinctorius*	[145]
		50, 100, 150 mg kg^−1^ Ni in soil	**⇑**	*Catharanthus roseus*	[70]
		0.1, 0.2, 0.3, 0.4 mM	**⇑**	*Chenopodium quinoa*	[137]
		100 mg kg^−1^ Ni in soil	**⇑**	*Cicer arietinum*	[89]
		70 mg kg^−1^ Ni in soil	**⇑**	*Citrullus lanatus*	[146]
		0.273 mM	**⇓**	*Cucurbita pepo*	[147]
		0.6 mM	**⇑**	*Eucalyptus urophylla*	[150]
		0.01, 0.02 mM	**⇑**	*Glycine max*	[92]
		0.2 mM	**⇑**		[151]
		2 mM	**⇑**		[106]
		0.05, 0.1 mM	**⇑**	*Gossypium hirsutum*	[135]
		0.005, 0.01, 0.02, 0.04 mM	**⇑**	*Hydrilla verticillata*	[83]
		1, 3, 4 mM	**⇓**	*Hydrocharis dubia*	[87]
		7.5–60 mg L^−1^	**⇑**	*Ipomoea batatas*	[153]
		400, 600 mg kg^−1^ Ni in soil	**⇑**	*Lactuca sativa*	[188]
		0.05–0.8 mM	**⇑**	*Luffa cylindrica*	[85]
		0.12 mM	**⇑**	*Matricaria chamomilla*	[193]
		50, 150, 250, 500 mg kg^−1^ Ni in soil	**⇑**	*Medicago sativa*	[154]
		20–100 mg kg^−1^ Ni in soil	**⇑**	*Mentha arvensis*	[71]
		0.1, 0.25, 0.5 mM	**⇑**	*Mentha piperita*	[155]
		0.1, 0.2, 0.4 mM	**⇑**	*Odontarrhena inflata*	[157,158]
		0.15, 0.3 mM	**⇑**		[138]
		0.05, 0.1, 0.2 mM	**⇑**	*O* *ryza sativa*	[129]
		0.05, 0.2 mM	**⇑**		[130]
		0.2, 0.4 mM	**⇑**		[134]
		80, 100 mg kg^−1^ Ni in soil	**⇑**		[97]
		0.023 mM	**⇑**	*Pennisetum typhoideum*	[112]
		0.1 mM	**⇑**	*Pisum sativum*	[159]
		0.1, 0.2, 0.5, 1 mM	**⇑**	*Raphanus sativus*	[191]
		0.03, 0.05 mM	**⇑**	*Solanum lycopersicum*	[161]
		0.05 mM	**⇑**		[72]
		0.4 mM	**⇑**		[164]
		0.05, 0.1, 0.2, 0.3, 0.4 mM	**⇓**	*Solanum melongena*	[165]
		0.3, 0.4, 0.5 mM	**⇓**	*Solanum tuberosum*	[192]
		0.1 mM	**⇑**	*Triticum aestivum*	[169]
		0.2 mM	**⇑**		[185]
		1–40 mM	**⇑**		[178]
		0.1, 0.5, 1 mM	**⇑**	*Vicia sativa*	[170]
		50, 100, 150 mg kg^−1^ Ni in soil	**⇑**	*Vigna radiata*	[73,74]
		50, 100, 150 mg kg^−1^ Ni in soil	**⇑**	*Vinca rosea*	[172]
		0.084, 0.168 mM	**⇑**	*Zea mays*	[173]
		0.1 mM	**⇑**		[176]
		0.25 mM	**⇑**		[174]

**⇓**— Activity of the enzyme decreased; **⇑**— activity of the enzyme increased; 0—activity of the enzyme did not change. The plants were grown in hydroponics (Ni concentration is given in mM or mg L^−1^) or in soil (mg kg^−1^ Ni in soil). Hyperaccumulator species are marked grey.

**Table 4:** The effects of Ni on the contents of non-enzymatic antioxidants.

Plant Species	Ni Content/ Concentration	Plant Organ	AsA	GSH	AN	PhOH	Flav	Pro	Refs.
*Anethum graveolens*	400 mg kg^−1^ Ni in soil	leaves			0	0	**⇓**	**⇑**	[82]
*Atropa belladonna*	0.05, 0.1, 0.15, 0.2 mM	leaves						**⇑**	[139,198]
*Bornmuellera emarginata*	0.001, 0.01, 0.1 mM	shoots						0 **⇓** ^(1)^	[8]
*Brassica juncea*	0.05, 0.1 mM	roots leaves						**⇑** **⇑**	[91]
	0.05, 0.1, 0.15 mM	leaves	**⇓**	**⇑**		**⇑**	**⇓**	**⇑**	[115]
	0.05, 0.1, 0.15 mM	plants	**⇓**	**⇑**				**⇑**	[116]
	0.15 mM	roots leaves						**⇑** **⇑**	[69]
	0.1, 0.2, 0.4 mM	seedlings						**⇑**	[140]
	0.2, 0.3, 0.4 mM	leaves						**⇑**	[186]
	200 mg kg^−1^ Ni in soil	leaves		**⇑**				**⇑**	[67,88]
*Brassica napus*	0.5 mM	leaves						**⇑**	[108]
	50, 100 mg kg^−1^ Ni in soil	leaves	**⇓**		**⇓**		**⇓**		[142]
*Brassica oleracea*	0.5 mM	leaves						**⇑**	[190]
*Cajanus cajan*	0.5, 1, 1.5 mM	roots leaves	**⇓** **⇓**	**⇓** **⇓**					[182]
*Capsicum annuum*	20–100 mg L^−1^	seedlings				**⇑**	**⇑**	**⇑**	[144]
*Carthamus* *oxyacantha*	0.5, 0.75, 1 mM	leaves			**⇑**		**⇑**		[145]
*Carthamus tinctorius*	0.5, 0.75, 1 mM	leaves			**⇑**		**⇑**		[145]
*Catharanthus roseus*	50, 100, 150 mg kg^−1^ Ni in soil	leaves						**⇑**	[70]
*Cicer arietinum*	100 mg kg^−1^ Ni in soil	leaves	0	**⇑**	**⇑**	0	0	**⇑**	[89]
*Cichorium intybus*	0.03 mM	roots shoots	0 0			**⇑** **⇑**			[197]
*Cucumis sativus*	0.2 mM	fruits	**⇑**						[118]
*Cucurbita pepo*	0.273 mM	roots leaves				**⇑** **⇑**	**⇑** 0	**⇑** **⇑**	[147,148]
*Eleusine coracana*	0.5 mM	roots shoots						**⇑** **⇑**	[109]
	1 mM	roots shoots		**⇑** 0				**⇑** **⇑**	[149]
*Glycine max*	2 mM	seedlings	**⇑**					**⇑**	[106]
	4 mM	leaves	**⇓**	0				**⇑**	[110]
*Hibiscus sabdariffa*	25, 50 mg kg^−1^ Ni in soil	calyces			**⇑**				[199]
*Hieracium* *aurantiacum*	0.03 mM	roots shoots	**⇑** 0			0 **⇑**			[197]
*Hydrocharis dubia*	0.5, 1, 2, 3, 4 mM	leaves						**⇑**	[87]
*Ipomoea batatas*	7.5, 15, 30, 60 mg L^−1^	leaves		**⇑**		**⇑**	**⇑**	**⇑**	[153]
*Lavandula angustifolia*	220, 330 mg kg^−1^ Ni in soil	leaves			**⇑**	**⇑**	**⇑**	**⇑**	[84]
*Lemna minor*	0.1, 0.2 mM	fronds	**⇑**	**⇑**					[183]
*Leontodon hispidus*	0.03 mM	roots shoots	**⇓** **⇓**			0 **⇑**			[197]
*Mentha arvensis*	20–100 mg kg^−1^ Ni in soil	leaves						**⇑**	[71]
*Odontarrhena inflata*	0.1, 0.2, 0.4 mM	roots shoots						**⇑** **⇑**	[157,158]
*Oryza sativa*	0.05, 0.2 mM	roots shoots	**⇑** **⇑**	**⇑ ^(^** **^2)^** **⇑ ^(^** **^2)^**				**⇑** **⇑**	[130]
	0.1, 0.2 mM	roots shoots	**⇑** **⇑**	**⇑** **⇑**				**⇑** **⇑**	[129]
	0.2, 0.4 mM	roots shoots	**⇑** **⇑**	**⇓** **⇓**				**⇑** **⇑**	[134,200]
	0.25, 0.5 mM	leaves	**⇓**	**⇑**				**⇑**	[111]
	80, 100 mg kg^−1^ Ni in soil	leaves	**⇑** ^(3)^	**⇑**	**⇑**	**⇑**	**⇑**	**⇑**	[97]
*Pennisetum typhoideum*	0.023 mM	radicles						**⇑**	[112]
*Pisum sativum*	0.1 mM	roots leaves						**⇑** **⇑**	[159]
*Salix viminalis*	1, 1.5, 2, 2.5, 3 mM	leaves				**⇑**			[201]
*Sesbania drummondii*	0.518 mM	seedlings		**⇑**					[184]
*Sesuvium portulacastrum*	0.05, 0.1 mM	shoots						**⇑**	[160]
*Solanum lycopersicum*	0.05 mM	roots leaves	**⇓** **⇑**	**⇓** **⇓**	**⇑**	**⇑**		**⇑**	[162]
	0.05 mM	roots leaves	**⇑** **⇑**	**⇑** **⇑**					[163]
	0.05 mM	plants				**⇑**		**⇑**	[72]
	1 mM	plants	**⇓**	**⇑**				**⇑**	[113]
*Solanum melongena*	0.1, 0.2, 0.3, 0.4 mM	leaves						**⇑**	[165]
	100 mg kg^−1^ Ni in soil	leaves	**⇓**	**⇑**				**⇑**	[114,166]
*Solanum nigrum*	0.1 mM	shoots						**⇑**	[167]
*Triticum aestivum*	0.2 mM	shoots						**⇑**	[185]
	0.1, 0.2 mM	roots leaves						**⇑** **⇑**	[136]
	0.5 mM	plants	**⇓**	**⇑**		**⇑**	**⇑**	**⇑**	[100]
*Vicia sativa*	0.001–1 mM	leaves						**⇑**	[170]
*Vigna mungo*	0.01, 0.05, 0.1 mM	leaves						**⇑**	[202]
*Vigna radiata*	0.2 mM	leaves	**⇑**					**⇑**	[171]
	150 mg kg^−1^ Ni in soil	leaves						**⇑**	[73,74]
*Vinca rosea*	50, 100, 200 mg kg^−1^ Ni in soil	leaves				**⇓**	**⇓**	**⇑**	[172]
*Zea mays*	0.1 mM	roots leaves	**⇑** **⇑**					**⇑** **⇑**	[175]
	0.1 mM	roots shoots	**⇑** **⇓**	0 **⇓**		0 0			[203]
	0.1 mM	leaves				**⇑**	**⇑**		[78]
	0.1 mM	roots leaves		**⇑** **⇑**					[176]

**⇓**—Decrease; **⇑**—increase; 0—no changes. The plants were grown in hydroponics (Ni concentration given in mM or mg L^−1^) or in soil (mg kg^−1^ Ni in soil). If the cells are empty, no measurements were taken. Ascorbate (AsA), anthocyanins (AN), glutathione (GSH), phenols (PhOH), flavonoids (Flav), proline (Pro). ^(1)^ Only at 0.01 mM; ^(2)^ only at 0.2 mM; ^(3)^ only at 100 mg kg^−1^ Ni in soil. Hyperaccumulator species are marked grey.

**Table 5:** The effects of Ni on fatty acid profiles in plants.

Fatty Acid	*Arabidopsis halleri*				*Arabidopsis lyrata*				*Triticum aestivum*			
	5 µM Ni		50 µM Ni		5 µM Ni		50 µM Ni		50 µM Ni		100 µM Ni	
	Roots	Shoots	Roots	Shoots	Roots	Shoots	Roots	Shoots	Roots	Shoots	Roots	Shoots
Myristic (14:0)	0	0	0	0	0	0	**⇓**	0				
Pentadecylic (15:0)	0	0	0	0	0	0	**⇓**	0				
Palmitic (16:0)	0	**⇑**	0	**⇑**	0	0	**⇓**	0	0	**⇑**	0	**⇑**
7-Hexadecenoic (7–16:1)	0	0	0	0	0	0	**⇓**	n.d.				
Palmitoleic (9–16:1)	0	0	0	0	0	0	**⇑**	0	0	**⇓**	0	**⇓**
Hexadecadienoic (7,10–16:2)	n.d.	0	0	0	0	0	0	0				
Roughanic (7,10,13–16:3)	0	**⇓**	0	**⇓**	**⇑**	**⇑**	**⇑**	0				
Stearic (18:0)	0	0	0	0	**⇓**	**⇓**	**⇓**	**⇓**	0	**⇑**	0	0
Oleic (9–18:1)	0	**⇑**	**⇑**	**⇑**	0	0	**⇑**	**⇑**	**⇑**	0	**⇑**	0
Vaccenic (11–18:1)	0	0	**⇑**	0	0	**⇓**	0	n.d.				
Linoleic (9,12–18:2)	**⇑**	**⇑**	0	**⇑**	0	0	**⇑**	0	**⇑**	**⇑**	0	**⇑**
Hexadecadienoic (10,13–18:2)	0	0	0	0	0	0	0	0				
α-Linolenic (9,12,15–18:3)	0	**⇓**	0	**⇓**	**⇑**	0	n.d.	0	**⇓**	**⇓**	**⇓**	**⇓**
α-Parinaric acid (9,11,13,15–18:4)	0	**⇑**	0	n.d.	0	n.d.	0	n.d.				

**⇓**—Relative content decreased; **⇑**—relative content increased; 0— relative content did not change; n.d.—not detemined. The plants were grown for 1 week on Hoagland`s nutrient solution. The data were taken from the works [220] (*Arabidopsis halleri*, *Arabidopsis lyrata*) and [168] (*Triticum aestivum*).

**Table 6:** The effects of Ni on the contents of macro- and microelements in plants.

Plant Species	Ni Content/ Concentration	Plant Organ	N	P	K	Ca	Mg	Fe	Mn	Cu	Zn	Refs.
*Anethum graveolens*	400 mg kg^−1^ Ni in soil	leaves	**⇓**		**⇓**	**⇓**	**⇓**	0			0	[82]
*Bornmuellera emarginata*	1, 10, 100 µM NiSO_4_	shoots						**⇓**	**⇓**	0	0	[8]
*Brassica juncea*	50, 100, 150 µM NiCl_2_	plants	**⇓**	**⇓**	**⇓**	**⇓**	**⇓**		**⇓**			[116]
	200 mg kg^−1^ Ni in soil	leaves	**⇓**	**⇓**	**⇓**	**⇓**						[88]
*Brassica napus*	125, 250, 500 µM NiCl_2_	leaves						**⇓**		**⇓**		[141]
	50, 100 mg kg^−1^ Ni in soil	roots shoots			**⇓** **⇓**	**⇓** **⇓**						[142]
*Brassica oleracea*	500 µM NiSO_4_	roots and shoots						**⇓**				[190]
*Capsicum annuum*	10–100 mg L^−1^ NiCl_2_ × 6 H_2_O	roots leaves	**⇓** **⇓**	**⇓** **⇓**	**⇓** **⇓**							[144]
*Chenopodium* *quinoa*	100, 200, 300, 400 µM NiCl_2_ × 6 H_2_O	roots shoots			**⇓** **⇓**							[137]
*Citrullus lanatus*	70 mg kg^−1^ Ni in soil	roots shoots	**⇓** **⇓**	**⇓** **⇓**	**⇓** **⇓**		**⇓** **⇓**	**⇓** **⇓**	**⇓** **⇓**		**⇓** **⇓**	[146]
*Cucurbita pepo*	273 µM Ni	roots leaves				**⇓** **⇓**	**⇓** **⇓**	**⇓** **⇓**	**⇓** **⇓**	**⇓** 0		[148]
*Eleusine coracana*	0.5 mM NiCl_2_ × 6 H_2_O	roots shoots					**⇓** **⇓**	**⇓** **⇓**			**⇓** **⇓**	[109]
*Eucalyptus* *urophylla*	600 µM Ni	roots stems leaves		**⇓** 0 **⇓**		**⇓** 0 **⇓**			**⇓** **⇓** **⇓**			[150]
*Glycine max*	0.1–20 µM Ni	roots		**⇓**	**⇓** ^(1)^	**⇓**		**⇑** ^(2)^	**⇓**	**⇑**	**⇓**	[92]
	200 µM NiCl_2_	roots stems leaves		**⇓** **⇓** **⇓**		**⇓** **⇓** **⇓**	**⇓** **⇓** 0	**⇓** **⇓** **⇓**	**⇓** **⇓** **⇓**		**⇓** **⇓** **⇓**	[151]
	0.5–9 mg kg^−1^ Ni in soil	roots leaves grains	**⇑** **⇑** ^(7)^		**⇑** ^(6)^		0	**⇑** **⇓** **⇑** ^(6)^		**⇑** **⇓** ^(8)^	**⇓** ^(8)^	[120]
*Helianthus* *annuus*	20–40 mg L^−1^ NiSO_4_	leaves	**⇓**	**⇓**	**⇓**	**⇓**	**⇓**				**⇓**	[229]
*Hibiscus* *sabdariffa*	25, 50 mg kg^−1^ Ni in soil	leaves	**⇑**	**⇑**	**⇑**			0	**⇑**	**⇑**	**⇑**	[199]
*Hordeum* *vulgare*	6 × 10^−7^, 1 × 10^−6^ M NiSO_4_	shoots						**⇓**				[50]
	10, 100 μM NiSO_4_	roots shoots						**⇑** **⇓**	**⇓** **⇓**	**⇑** **⇓**	**⇓** **⇓**	[230]
*Hydrocharis* *dubia*	0.5, 1, 2, 3, 4 mM Ni	leaves				**⇑** ^(5)^				**⇓**	**⇑**	[87]
*Hypericum olympicum*	100 μM Ni	roots leaves		0 0	**⇓** 0	**⇓** **⇓**	0 **⇓**	0 **⇓**	0 0	0 0	**⇑** **⇓**	[231]
*Hypericum oriental*	100 μM Ni	roots leaves		**⇓** **⇓**	**⇓** **⇓**	**⇓** 0	**⇓** 0	**⇓** 0	**⇓** 0	0 **⇓**	**⇓** **⇓**	[231]
*Hypericum perforatum*	100 μM Ni	roots leaves		0 **⇓**	0 **⇓**	0 **⇓**	0 **⇓**	0 0	0 0	0 0	0 **⇓**	[231]
*Lolium* *perenne*	30, 90, 180, 270 mg kg^−1^ Ni in soil	shoots						**⇑** **⇓**	**⇓**		**⇓**	[232]
*Matricaria* *chamomilla*	120 μM NiCl_2_ × 6 H_2_O	roots leaves			**⇓** **⇓**		0 0	**⇑** **⇑**		**⇑** 0		[193]
*Noccaea* *caerulescens*	10, 50, 100 μM Ni	plants						**⇑** ^(4)^	**⇓**	0	**⇓** ^(3)^	[233]
*Odontarrhena* *inflata*	100, 300 μM NiSO_4_	roots shoots						**⇑** **⇓**				[81]
*Oryza sativa*	5 × 10^−4^ M NiCl_2_	roots and shoots			**⇓**	**⇓**	**⇓**					[234]
	80, 100 mg kg^−1^ Ni in soil	roots leaves	**⇓** **⇓**	**⇓** **⇓**	**⇓** **⇓**	**⇓** **⇓**						[97]
*Phaseolus* *vulgaris*	3.4–14 × 10^−6^ M Ni(NO_3_)_2_ × 6 H_2_O	roots leaves	**⇑** **⇑**	**⇑** **⇑**	**⇑** 0	0 0	0 0	**⇑** 0	0 0	**⇑** **⇑**	0 0	[235]
*Psidium guajava*	300, 1000, 3000 μM NiSO_4_	roots leaves			**⇓** 0	0 0						[236]
*Solanum* *lycopersicum*	50 μM Ni	roots leaves		0 **⇓**	**⇓** **⇓**	**⇓** **⇑**	**⇓** **⇓**	**⇑** **⇓**	**⇓** **⇓**		**⇑** **⇑**	[162]
	50 μM Ni	roots leaves	**⇓** **⇓**	**⇓** **⇓**	**⇓** **⇓**	**⇓** **⇓**	**⇓** **⇓**	**⇓** **⇓**	**⇓** **⇓**		**⇑** **⇑**	[163]
	30 μM Ni	roots leaves						**⇓** **⇓**	**⇓** **⇓**	**⇑** **⇓**	**⇑** **⇓**	[161]
	100 μM NiSO_4_	roots shoots fruits	**⇓** **⇓** **⇓**	**⇓** **⇓** **⇓**	**⇓** **⇓** **⇓**	**⇓** **⇓** **⇓**	**⇓** **⇓** **⇓**	**⇓** **⇓** **⇓**	**⇓** **⇓** **⇓**	**⇓** **⇓** **⇓**	**⇓** **⇓** **⇓**	[237]
	400 μM Ni	roots stems leaves		**⇓** 0 **⇓**		**⇓** **⇓** **⇓**			**⇓** **⇓** **⇓**	**⇓** **⇓** **⇓**	**⇓** **⇓** **⇓**	[164]
	1 mM NiSO_4_ × 6 H_2_O	plants	**⇓**	**⇓**	**⇓**		**⇓**					[113]
*Solanum* *melongena*	200, 300, 400 μM NiSO_4_	roots stems leaves						**⇑** **⇓** **⇓**				[165]
	100 mg kg^−1^ Ni in soil	leaves	**⇓**		**⇓**	**⇓**						[114]
*Solanum* *tuberosum*	0.4, 0.5 mM NiSO_4_	roots stems leaves			**⇓** **⇑** **⇑**			**⇑** **⇓** **⇓**			**⇑** **⇓** **⇓**	[192]
*Triticum* *aestivum*	0.5 mM Ni	plants	**⇓**		**⇓**	**⇓**						[100]
	1 mM NiCl_2_ × 6 H_2_O	roots and leaves			**⇓**	**⇓**	**⇓**	**⇓**				[238]
	5–40 × 10^−3^ M NiSO_4_×7 H_2_O	roots and shoots			**⇓**							[178]
	67 × 10^−6^ M Ni	roots leaves				0 **⇑**	0 **⇑**	0 **⇓**	**⇓** **⇓**	0 0	0 **⇓**	[239]
*Triticum* *durum*	67 × 10^−6^ M Ni	roots leaves				0 0	0 0	0 **⇓**	**⇓** **⇓**	0 0	0 0	[239]
*Noccaea montana* (*Thlaspi* *montanum*)	soil (in the natural environment)	leaves			**⇑**	**⇑**						[240]
*Vigna radiata*	100, 200 mg kg^−1^ Ni in soil	roots shoots		**⇓** **⇓**	**⇓** **⇓**	**⇓** **⇓**	**⇓** **⇓**	**⇓** **⇓**				[241]
*Zea mays*	10^−5^ M Ni	roots shoots				**⇓** 0	**⇓** **⇑**	**⇑** **⇓**	**⇓** **⇓**	**⇓** **⇓**	**⇑** **⇓**	[242]
	100 μM NiCl_2_	roots shoots		**⇓** **⇓**	**⇓** **⇓**	**⇓** **⇓**	**⇓** **⇓**	**⇓** **⇓**	**⇓** **⇓**			[176]
	40 μM Ni	roots stems leaves grains		**⇓** **⇓** **⇓** **⇓**	**⇑** **⇑** **⇑** **⇑**							[103]
	40 mg L^−1^ NiCl_2_	roots shoots	0 **⇓**	**⇓** **⇓**	**⇓** **⇓**	**⇓** **⇓**	**⇓** **⇓**	**⇓** **⇓**	**⇓** **⇓**	**⇓** 0	**⇓** **⇓**	[173]

**⇓**—Content of the element decreased; **⇑**—content of the element increased; 0—content of the element did not change. Empty cells mean lack of data. ^(1)^ Only at 10 and 20 µM Ni; ^(2)^ only at 0.5, 10, 20 µM Ni; ^(3)^ only at 10 µM Ni; ^(4)^ only at 100 µM Ni; ^(5)^ except for 2 mM Ni; ^(6)^ at 1–9 mg kg^−1^ Ni; ^(7)^ except for 9 mg kg^−1^ Ni; ^(8)^ only at 1, 3 mg kg^−1^ Ni. The plants were grown in hydroponics (Ni concentration given in mM/µM/M or mg L^−1^) or in soil (mg kg^−1^ Ni in soil). Hyperaccumulator species are marked grey.

**Table 7:** The effects of Ni on photosynthesis.

Plant Species	Ni Content/ Concentration	Chl	Car	*P*_n_	*F*_0_	*F*_m_	*F*_v_/*F*_m_	*F*_0_/*F*_v_	qP	NPQ	*J*_max_ *ETR*	*V*_cmax_	*g*_s_	*C*_i_	*g*_m_	*C*_c_	Refs.
*Alyssoides* *utriculata*	10, 100, 500 mg L^−1^				0	0	0										[321]
*Amaranthus paniculatus*	25–150 µM	**⇓**		**⇓**	**⇑**	0	**⇓**		**⇓** ^(3)^	**⇑** **⇓** ^(4)^							[86]
*Brassica* *juncea*	50, 100 µM	**⇓**		**⇓**													[91]
	100, 150 µM	**⇓**	**⇓**	**⇓**									**⇓**	**⇓**			[116]
	150 µM	**⇓**	**⇓**	**⇓**									**⇓**	**⇓**			[69]
	200 mg kg^−1^ in soil	**⇓**		**⇓**			**⇓**		**⇓**	**⇑**	**⇓**		**⇓**	**⇓**			[66,67,88]
*Eucalyptus urophylla*	600 µM	**⇓**	**⇓**	**⇓**	**⇑**	**⇑**	**⇓**						**⇓**	**⇓**			[150]
*Capsicum annuum*	20–100 mg L^−1^	**⇓**	**⇓**	**⇓**			**⇓**		**⇓**	**⇑**			**⇓**	**⇓**			[144]
*Cicer* *arietinum*	100 mg kg^−1^ in soil	**⇓**	**⇓**	**⇓**									**⇓**	**⇓**			[89]
*Citrullus* *lanatus*	70 mg kg^−1^ in soil	**⇓**	**⇓**	**⇓**									**⇓**	**⇓**			[72]
*Glycine max*	0.1 µM	**⇓**	**⇓**	**⇓**									**⇓**				[117]
	0.2 µM	**⇓**	**⇓**	**⇓**	**⇑**	**⇓**	**⇓**		**⇓**	**⇑**	**⇓**		**⇓**	**⇑**			[151]
	2 µM	**⇓**					**⇓**		**⇓**	**⇑**							[106]
	0.1–20 µM			**⇓**									**⇓**	**⇑** ^(2)^			[92]
*Gossypium hirsutum*	50, 100 µM	**⇓**		**⇓**									**⇓**				[135]
*Helianthus annuus*	20, 30, 40 mg L^−1^	**⇓**	**⇓**	**⇓**									**⇓**				[285]
*Ipomoea* *batatas*	60 mg L^−1^	**⇓**	**⇓**	**⇓**									**⇓**	**⇓**			[153]
*Ocimum* *basilicum*	150 mg kg^−1^ in soil	**⇓**	**⇓**			**⇓**	**⇓**										[156]
*Odontarrhena* *chalcidica*	250 1000 µM	0 0	0 0	**⇑** **⇑**			0 0			**⇓** **⇓**	**⇑** **⇑**		0 **⇑**	**⇓** 0	0 0		[288]
*Odontarrhena* *moravensis*	250 1000 µM	0 0	0 0	**⇑** **⇓**			0 **⇓**			**⇓** **⇑**	**⇑** **⇓**		0 **⇓**	**⇓** 0	0 **⇓**		[288]
*Odontarrhena* *muralis*	250 1000 µM	**⇓** **⇓**	0 0	**⇓** **⇓**			0 **⇓**			**⇑** **⇑**	**⇓** **⇓**		**⇓** **⇓**	0 0	**⇓** **⇓**		[288]
*Oryza sativa*	200 µM	**⇓**	**⇓**	**⇓**									**⇓**	**⇓**			[94]
	80, 100 mg kg^−1^ in soil						**⇓**		**⇓**	**⇑**							[97]
*Pisum* *sativum*	100 µM	**⇓**		**⇓**									**⇓**	**⇓**			[159]
*Populus nigra*	30, 200 µM			**⇓**	**⇑**	**⇓**	**⇓**	**⇑**			**⇓**	**⇓**	**⇓**	**⇓** ^(1)^	**⇓**	**⇓** ^(1)^	[281]
*Solanum lycopersicum*	30, 50 µM	**⇓**					**⇓**		**⇓** ^(5)^	**⇑**							[161]
	50 µM	**⇓**					**⇓**						**⇓**	**⇓**			[162]
	50 µM	**⇓**											**⇓**	**⇓**			[163]
	50 µM			**⇓**									**⇓**	**⇓**			[72]
	400 µM	**⇓**	**⇓**	**⇓**	**⇑**	**⇓**	**⇓**		**⇓**	**⇑**	**⇓**		**⇓**	**⇑**			[164]
	1000 µM	**⇓**	**⇓**	**⇓**			**⇓**		**⇓**	**⇑**	**⇓**		**⇓**	**⇓**			[113]
*Solanum melongena*	100 mg kg^−1^ in soil	**⇓**	**⇓**	**⇓**									**⇓**	**⇓**			[114,166]
*Triticum aestivum*	500 µM	**⇓**	**⇓**	**⇓**			**⇓**										[100]
*Vigna radiata*	150 mg kg^−1^ in soil	**⇓**		**⇓**			**⇓**						**⇓**	**⇓**			[73]
*Vigna* *unguiculata*	0.5–3 mg kg^−1^ in soil	**⇑**	0	**⇑** ^(6)^									**⇑** ^(7)^	**⇑** ^(8)^			[102]
*Vinca rosea*	50–200 mg kg^−1^ in soil	**⇓**		**⇓**									**⇓**				[172]
*Zea mays*	100 µM					**⇓**	**⇓**		**⇓**	**⇑**	**⇓**						[78]
	100 µM	**⇓**		**⇓**			**⇓**						**⇓**	**⇓**			[176]

**⇓**—Decrease; **⇑**—increase; 0—no changes. Empty cells mean lack of data. Chlorophyll content (Chl), carotenoid content (Car), net photosynthetic rate (*P*n (*A*), μmol CO_2_ m^−2^ s^−1^), initial (*F*_0_) and maximal (*F*_m_) fluorescence yield, maximum quantum efficiency of PSII (*F*_v_/*F*_m_), efficiency of water-splitting apparatus (*F*_0_/*F*_v_), photochemical quenching (qP), non-photochemical quenching (NPQ), electron transport rate (ETR, μmol e^−^ m^−2^ s^−1^) or maximum light-driven electron transport rate (*J*_max_, μmol m^−2^ s^−1^), maximum velocity of carboxylation (*V*_cmax_, μmol m^−2^ s^−1^), stomatal conductance (*g*_s_, mol m^−2^ s^−1^), intracellular CO_2_ concentration (*C*_i_, mmol mol^−1^), mesophyll conductance (*g*_m_, mol m^−2^ s^−1^), CO_2_ concentration at carboxylation site (*C*_c_, mmol mol^−1^). ^(1)^ only at 200 µM Ni, ^(2)^ only at 10 and 20 µM Ni, ^(3)^ only at 50–150 µM Ni, ^(4)^ increased at 50 µM Ni, decreased at 150 µM Ni, did not change at 25 and 100 µM Ni; ^(5)^ only at 50 µM Ni; ^(6)^ at 0.5–2 mg kg^−1^; ^(7)^ only at 0.5 and 1 mg kg^−1^; ^(8)^ only at 0.5 mg kg^−1^. The plants were grown in hydroponics (Ni concentration given in µM or mg L^−1^) or in soil (mg kg^−1^ Ni in soil). Hyperaccumulator species are marked grey.

**Table 8:** The effects of Ni on plant growth.

Plant Species	Ni Content/ Concentration	RL	SL	LL	RFW	RDW	SFW	SDW	LFW	LDW	RI	LA	NL	Refs.
*Amaranthus* *paniculatus*	0.05, 0.1, 0.15 mM					**⇓**				**⇓**		**⇓**		[295]
*Arabidopsis* *thaliana*	0.05, 0.075 mM	**⇓**										**⇓**		[358]
*Brassica campestris*	0.02 mM	**⇓**				**⇓**		**⇓**						[181]
*Brassica juncea*	0.05, 0.1 mM	**⇓**	**⇓**		**⇓**	**⇓**	**⇓**	**⇓**						[91]
	0.05, 0.1, 0.15 mM	**⇓**	**⇓** ^(7)^											[115]
	0.15 mM	**⇓**	**⇓**									**⇓**		[69]
	200 mg kg^−1^ Ni in soil											**⇓**		[66]
*Brassica napus*	0.5 mM					**⇓**		**⇓**						[108]
	50, 100, 150 mg L^−1^						**⇓**	**⇓**						[282]
	50, 100 mg kg^−1^ Ni in soil	**⇓**	**⇓**		**⇓** ^(13)^	**⇓**	**⇓**	**⇓**						[142]
*Cajanus cajan*	0.5, 1, 1.5 mM					**⇓**		**⇓**						[182]
*Calendula* *tripterocarpa*	100, 150 mg kg^−1^ Ni in soil				**⇓**	**⇓**	**⇓** ^(8)^	**⇓**						[143]
*Capsella* *bursa-pastoris*	0.01, 0.02, 0.03, 0.04 mM					**⇓** ^(3)^		**⇓**						[325]
*Capsicum annuum*	10, 20, 30, 50, 75, 100 mg L^−1^	**⇓**			**⇓**	**⇓**	**⇓**	**⇓**						[144]
*Carthamus* *oxyacantha*	0.5, 0.75, 1 mM					**⇓**		**⇓**						[145]
*Carthamus* *tinctorius*	0.5, 0.75, 1 mM					**⇓**		**⇓**						[145]
*Catharanthus roseus*	50, 100, 150 mg kg^−1^ Ni in soil				**⇓**	**⇓**	**⇓**	**⇓**				**⇓**		[70]
*Chenopodium* *quinoa*	0.1, 0.2, 0.3, 0.4 mM	**⇓**	**⇓**			**⇓**		**⇓**						[137]
*Cicer arietinum*	100 mg kg^−1^ Ni in soil	**⇓**	**⇓**		**⇓**	**⇓**	**⇓**	**⇓**						[89]
*Citrullus lanatus*	70 mg kg^−1^ Ni in soil	**⇓**												[146]
*Eleusine coracana*	0.5 mM	**⇓**	**⇓**			**⇓**		**⇓**						[109]
	0.1, 0.25, 0.5, 1 mM	**⇓**	**⇓ ^(^** ^11)^		**⇓**	**⇓**	**⇓**	**⇓**						[149]
*Eruca sativa*	150, 250, 500 mg kg^−1^ Ni in soil	**⇓** ^(4)^	**⇓**											[308]
*Eucalyptus* *urophylla*	0.6 mM					**⇓**				**⇓**				[150]
*Glycine max*	0.0005, 0.01, 0.02 mM					**⇓**		**⇓**						[92]
	0.1 mM	**⇓**	**⇓**											[117]
	0.2 mM					**⇓**				**⇓**				[151]
	2 mM	**⇓**	**⇓**											[106]
	200 mg kg^−1^ Ni in soil		**⇓**					**⇓**				**⇓**		[152]
*Gossypium hirsutum*	0.05, 0.1 mM	**⇓**			**⇓**	**⇓**			**⇓**	**⇓**		**⇓**	**⇓**	[135]
*Helianthus annuus*	20, 30, 40 mg L^−1^	**⇓**	**⇓** ^(2)^		**⇓**	**⇓**	**⇓**	**⇓**						[229,285]
*Hordeum vulgare*	0.001, 0.01 mM 0.1 mM					**⇑** **⇓**		**⇑** **⇓**						[230]
*Ipomoea batatas*	15 mg L^−1^ 30, 60 mg L^−1^	**⇑** **⇓**			**⇑** **⇓**	**⇑** **⇓**	**⇑** **⇓** ^(10)^	**⇑** **⇓** ^(10)^				0**⇓**	**⇑** **⇓**	[153]
*Lens culinaris*	10, 20, 30, 60, 90 mg kg^−1^ Ni in soil					0		**⇓** ^(^^14)^						[93]
*Lepidium ruderale*	0.01, 0.02, 0.03, 0.04 mM					**⇓**		**⇓** ^(3)^						[325]
*Luffa cylindrica*	0.05–0.8 mM				**⇓**									[85]
*Medicago* *sativa*	50, 150, 250, 500 mg kg^−1^ Ni in soil	**⇓**	**⇓**		**⇓**	**⇓** ^(9)^	**⇓**	**⇓** ^(9)^						[154]
*Mentha piperita*	0.1, 0.25, 0.5 mM	**⇓**	**⇓**		**⇓**	**⇓**			**⇓**	**⇓**		**⇓**	**⇓**	[155]
*Ocimum basilicum*	100, 150 mg kg^−1^ Ni in soil					**⇓**	**⇓**	**⇓**	**⇓**	**⇓**		**⇓**	**⇓**	[156]
*Odontarrhena* *inflata*	0.4 mM				**⇓**									[157]
*Oryza sativa*	0.01, 0.05, 0.1, 0.2 mM	**⇓** ^(5)^	**⇓** ^(6)^		**⇓**	**⇓**	**⇓**	**⇓** ^(6)^						[129,130]
	0.2, 0.4 mM	**⇓**	**⇓** ^(1)^		**⇓**		**⇓**							[134]
	0.25, 0.5 mM	**⇓**	**⇓**											[111]
	80, 100 mg kg^−1^ Ni in soil	**⇓**	**⇓**		**⇓**	**⇓**	**⇓**	**⇓**				**⇓**		[97]
*Phaseolus vulgaris*	0.1, 0.2, 0.5 mM											**⇓**		[292]
*Pisum sativum*	0.1 mM											**⇓**		[159]
*Psidium guajava*	1, 3 mM					**⇓**		**⇓**						[236]
*Salix viminalis*	1, 1.5, 2, 2.5, 3 mM	**⇓**	**⇓**	**⇓**								**⇓**		[201]
*Solanum lycopersicum*	0.03, 0.05 mM				**⇓** ^(12)^	0			**⇓**	**⇓** ^(12)^		**⇓**		[161]
	0.05 mM	**⇓**	**⇓**		**⇓**	**⇓**	**⇓**	**⇓**						[72,163]
	0.4 mM					**⇓**				**⇓**				[164]
*Solanum melongena*	100 mg kg^−1^ Ni in soil	**⇓**	0		0	**⇓**	**⇓**	**⇓**						[166]
*Solanum nigrum*	0.1 mM	**⇓**	**⇓**		**⇓**		**⇓**							[167]
*Spinacia oleracea*	0.1, 0.2 mM	**⇓**	**⇓**											[359]
*Thlaspi arvense*	0.025, 0.03 mM					**⇓**				**⇓**				[360]
*Triticum aestivum*	0.01 mM 0.2 mM		0 **⇓**				**⇑** **⇓**							[185]
	1–40 mM	**⇓**	**⇓**			**⇓**		**⇓**						[178]
	150 mg kg^−1^ Ni in soil	**⇓**	**⇓**											[287]
*Vigna radiata*	0.001 mM 0.01, 0.1, 1 mM					**⇑** **⇓**		**⇑** **⇓**				**⇑** **⇓**		[284]
	0.01, 0.1, 0.2 mM					**⇓**				**⇓**				[171]
	50, 100, 150 mg kg^−1^ Ni in soil	**⇓**	**⇓**		**⇓**	**⇓**	**⇓**	**⇓**				**⇓**		[73,74]
	100, 150 mg kg^−1^ Ni in soil	**⇓**	**⇓**									0	**⇓**	[361]
	200 mg kg^−1^ Ni in soil				**⇓**	0	**⇓**	0				**⇓**	0	[241]
*Vinca rosea*	50, 100, 200 mg kg^−1^ Ni in soil	**⇓**	**⇓**		**⇓**	**⇓**	**⇓**	**⇓**					0	[172]
*Zea mays*	0.035 mM										**⇓**			[351,352]
	0.015–0.035 mM	**⇓**	**⇓**								**⇓**			[362]
	0.04 mM	**⇓**	**⇓**		**⇓**	**⇓**	**⇓**	**⇓**						[103]
	0.1 mM	**⇓**	**⇓**		**⇓**	**⇓**	**⇓**	**⇓**				**⇓**		[175,176]
	40 mg L^−1^	**⇓**	**⇓**		**⇓**	**⇓**	**⇓**	**⇓**						[173]

**⇓**—Decrease; **⇑**—increase; 0—no changes. Empty cells mean lack of data. Root length (RL), shoot length (SL), leaf length (LL), root fresh weight (RFW), root dry weight (RDW), shoot fresh weight (SFW), shoot dry weight (SDW), leaf fresh weight (LFW); leaf dry weight (LDW), root increment (RI), leaf area (LA), number of leaves (NL). ^(1)^ Only at 0.4 mM; ^(2)^ only at 40 mg L^−1^; ^(3)^ except for 0.01 mM; ^(4)^ except for 150 mg kg^−1^ Ni in soil; ^(5)^ only at 0.1 and 0.2 mM; ^(6)^ except for 0.05 mM; ^(7)^ except for 0.05 mM; ^(8)^ only at 150 mg kg^−1^ Ni in soil; ^(9)^ except for 50 mg kg^−1^ Ni in soil; ^(10)^ did not change at 30 mg L^−1^; ^(11)^ only at 0.5 and 1 mM; ^(12)^ only at 0.05 mM; ^(13)^ only at 100 mg kg^−1^ Ni in soil; ^(14)^ only at 60 and 90 mg kg^−1^ Ni in soil. The plants were grown in hydroponics (Ni concentration given in mM or mg L^−1^) or in soil (mg kg^−1^ Ni in soil). Hyperaccumulator species are marked grey.

**Table 9:** The range of metal toxicity in several plant species.

Plant Species	Range of Metal Toxicity	Refs.
*Agrostis* spp.	Ni > Pb > Zn > Cu	[373]
*Glycine max*	Cd > Cu > Ni > Zn	[374]
*Hydrilla verticillata*	Cd > Ni > Pb	[83]
*Hordeum vulgare*	Hg > Pb > Cu > Cd > Cr > Ni > Zn	[61]
*Lactuca sativa*	Cd > Ni > Cu > Zn > Hg > As > Mn > Cr > Pb > Fe	[372]
*Linum usitatissimum*	As (III) > As (V) > Cu > Cd > Co > Cr (VI) > Ni > Pb > Cr (III) > Zn	[371]
*Lolium perenne*	Cu > Ni > Mn > Pb > Cd > Zn > Al > Hg > Cr > Fe	[368]
*Triticum aestivum*	Cu > Cr > Ni > Zn > Pb ≈ Cd > Al > Fe	[375]
*Vicia faba*	Cd > Ni > Zn ≈ Co	[375]

## Data Availability

No new data were created or analyzed in this study. Data sharing is not applicable to this article.
